# Continuous Flow Synthesis of Benzotriazin-4(3*H*)-ones via Visible Light Mediated Nitrogen-Centered Norrish Reaction

**DOI:** 10.1021/acs.orglett.4c00248

**Published:** 2024-03-11

**Authors:** Jorge García-Lacuna, Marcus Baumann

**Affiliations:** University College Dublin, School of Chemistry, Science Centre South, Dublin 4, Ireland

## Abstract

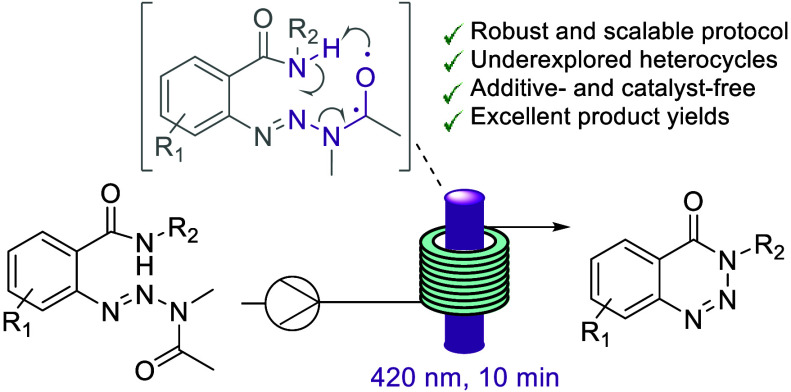

We report a new protocol for the synthesis of substituted benzotriazin-4(3*H*)-ones which are underrepresented heterocyclic scaffolds with important pharmacological properties. Our method exploits acyclic aryl triazine precursors that undergo a photocyclization reaction upon exposure to violet light (420 nm). Continuous flow reactor technology is exploited to afford excellent yields in only 10 min residence time with no additives or photocatalysts needed. The underlying reaction mechanism appears to be based on an unprecedented variation of the classical Norrish type II reaction with concomitant fragmentation and formation of N–N bonds. Scalability, process robustness, and green credentials of this intriguing transformation are highlighted.

Benzotriazin-4(3*H*)-ones are important heterocyclic scaffolds with various reported biological activities. These account for applications of this scaffold in active pharmaceutical ingredients such as anesthetics, antidepressants, and agrochemicals.^1,2^ Furthermore, benzotriazin-4(3*H*)-ones are versatile building blocks for metal-catalyzed^3^ as well as photochemical^4^ denitrogenative transformations toward related heterocycles^3a,3d,4a^ and for different cross-coupling reactions.^3e,3f,4b,4c^ Acid-mediated denitrogenative ortho-hydroxylation^5^ and heteroannulation reactions yielding benzo[*c*][1,2]dithiol-3-ones^6^ represent further useful applications of this valuable albeit underutilized heterocycle.

The most common approach for preparing benzotriazin-4(3*H*)-ones exploits the diazotization of 2-aminobenzamide or methyl anthranilate; however, the associated use of strong acids and NaNO_2_ renders this method problematic and limits its scope (Scheme 1a).^1,7^ Therefore, alternative routes to these attractive scaffolds have been developed in recent years. Modifications include a mild protocol using a polymer-supported nitrite reagent and *p*-tosic acid.^8^ Further variations were presented by Liu and collaborators using nitromethane^9^ or *tert*-butylnitrite^10^ as the nitrogen source and avoid the harsh acidic conditions required for diazonium salt formation reactions.

**Scheme 1:**
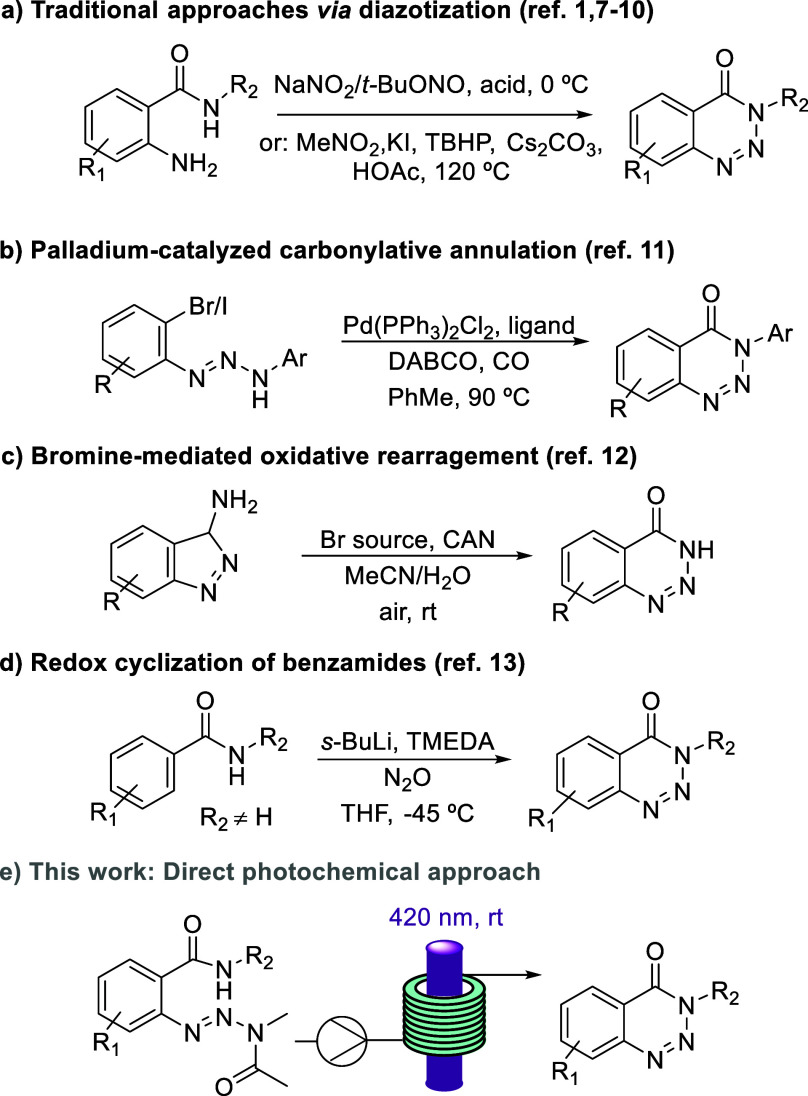
Strategies toward Benzotriazin-4(3*H*)-ones

In addition, Sankararaman and co-workers reported a Pd-catalyzed annulation reaction converting 1,3-diaryltriazenes into benzotriazin-4(3*H*)-ones in the presence of CO (Scheme 1b).^11^ Moreover, an oxidative rearrangement of 3-aminoindazoles was reported by Song and co-workers in 2018 (Scheme 1c).^12^ Lastly, a redox cyclization of benzamides using nitrous oxide after the treatment with *sec*-butyl lithium was described by Cui and co-workers (Scheme 1d).^13^ The precedented methodology for accessing the target heterocycle (Scheme 1a–d) clearly shows a lack of mild and green methods that are readily scalable and user-friendly. To contribute to this field, we wished to develop an efficient route to access benzotriazin-4(3*H*)-ones with and without substituents in the 3-position (N–H vs N–R). To achieve this, we opted to target a photochemical method in combination with continuous flow processing^14^ which would render a robust and reliable method toward these important heterocyclic scaffolds.

The photochemical flow setup consisted of a Vapourtec E-series reactor and its UV-150 photomodule equipped with a coil reactor (10 mL volume, PFA tubing) and different LEDs as light sources. One peristaltic pump was used as an adjustable back-pressure regulator (BPR). Substrate **1a** (Table 1) was used as model substrate during reaction optimization. This was based on knowledge from our previous study^15^ that showed that the triazine moiety of aryl benzoic acids is susceptible to photochemical N–N single bond fragmentation in the presence of an external secondary amine and subsequent expulsion of an acetamide leaving group. The starting benzamides were constructed via photochemical rearrangement of nitroarenes^15^ followed by amide coupling as described in the SI.

**Table 1:**
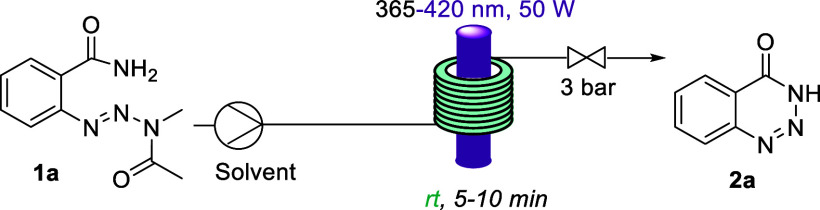
Optimization toward Benzotriazin-4(3*H*)-one **2a**

Entry^a^	Solvent (mM)	Wavelength	Yield (SM%)^b^
1	MeCN (18)	365 nm	41 (57)
2	AcOEt (35)	365 nm	30 (54)
3	MeOH (38)	365 nm	97
4	MeCN/H_2_O (2/1) (38)	365 nm	96
5	DCM (50)	365 nm	84
6	Acetone (35)	365 nm	0 (99)
7	DCM/MeOH (1/3) (50)	365 nm	95
8	DCM/MeOH (1/3) (50)	420 nm	96^c^
9^d^	DCM/MeOH (1/3) (50)	420 nm	68 (28)
10	DCM/MeOH (1/3) (50)	–	0 (99)

a Reaction conditions: Unless otherwise specified all the reactions were performed at 0.3 mmol scale in the corresponding solvent (homogeneous solution at 22–25 °C) with a residence time of 10 min (flow rate 1 mLmin-^1^) using the corresponding high-power LEDs with an input power of 50 W and a system pressure of 3 bar.

b qNMR yields were calculated using 1,3,5-trimethoxybenzene as the internal standard.

c 88% isolated yield (0.91 mmol scale).

d 5 min residence time (2 mL/min).

Initial reactions used a high-power LED emitting light at 365 nm in combination with a flow rate of 1 mL min^–1^. After a short optimization, almost quantitative yields of **2a** were obtained. The main challenge at this stage was to find a suitable solvent that fully dissolved the benzamide starting material at an appropriate concentration. Two solvent combinations gave almost quantitative yields MeCN/H_2_O (entry 4) and MeOH/DCM (entry 7). The use of MeOH alone (entry 3) was discarded, as some precipitation appeared at the end of the reactor. Product isolation after silica gel column chromatography gave a yield of 90% in both cases. Importantly, we were able to also use visible light (420 nm, entry 8) instead of UV-A light with full conversion of the substrate observed in only 10 min. *N*-Methyl acetamide was found in all cases as the only byproduct of the reaction which was removed by extraction and/or chromatography and can be isolated in near-quantitative yield. An isolated yield of 88% was obtained at 200 mg scale (0.91 mmol) for this reaction (entry 8), following an extractive workup instead of chromatography. Finally, the reaction was attempted in the dark, with no conversion observed (entry 10). Equally, thermal reactions (50 and 100 °C, no irradiation) returned starting material quantitatively, thus demonstrating the necessity of violet light to trigger this process.

With the optimized conditions in hand, the substrate scope was explored, focusing on both aryl substitution and introduction of different groups at the 3-position. As shown in Scheme 2, various substituents were tolerated on the aryl ring affording the desired products in excellent yields (**2b**–**2e**). Secondary amides were also tested as starting materials in combination with different aryl substituents, rendering the corresponding benzotriazin-4(3*H*)-one products in high yields (75–97%). Notable examples include (cyclo)alkyl groups (**2f**–**g**), heterocycles (**2h**), benzylic systems (**2i**), and aniline derivatives (**2j**). Moreover, a secondary amide derived from *rac*-phenylalanine (**2k**) was also transformed to the heterocyclic target in an 88% yield. Substrates decorated with substituents in both positions (**2l**–**p**) gave excellent results except for **2o** with a more moderate yield (54%). Interestingly, all the substrates tested afforded the reaction product, with the only deviation from the optimized conditions being the solvent used to provide full solubility of both starting material and product. The structure of product **2n** was confirmed by single crystal X-ray crystallography clearly showing the benzotriazin-4(3*H*)-one scaffold generated.

**Scheme 2:**
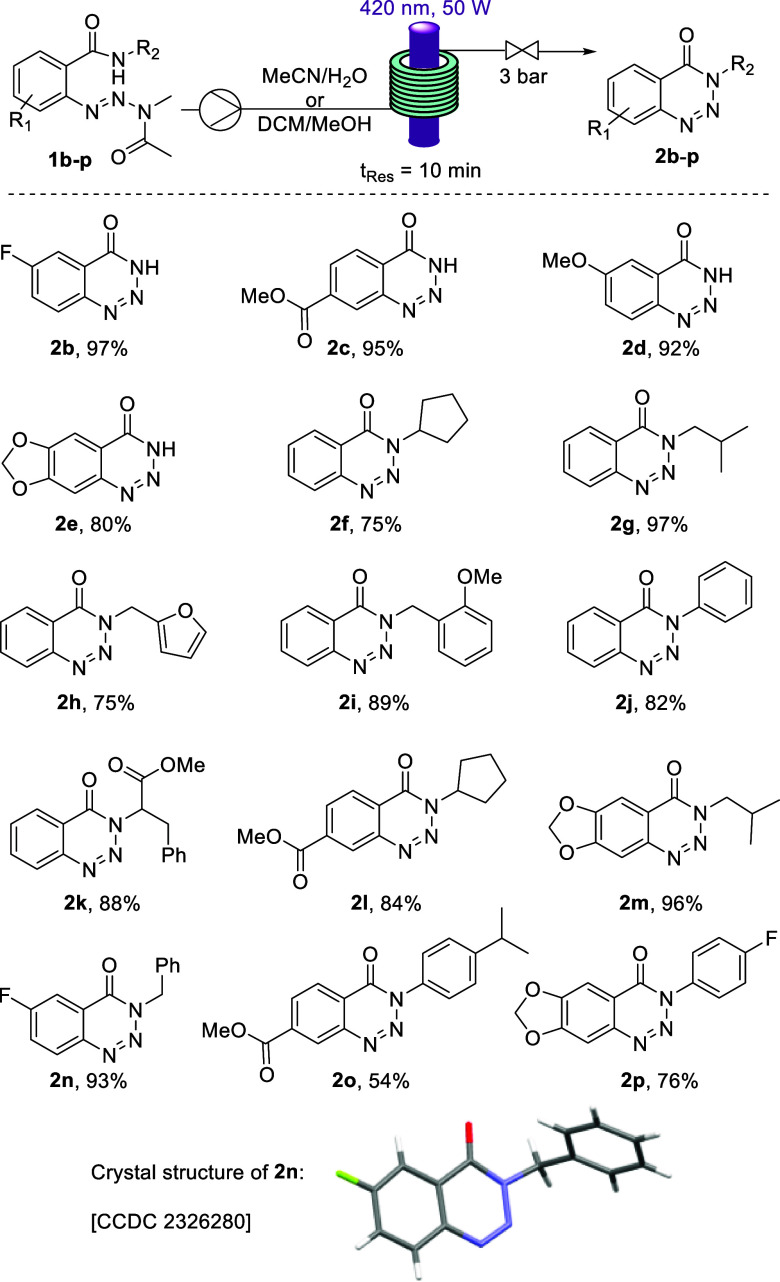
Reaction Scope of the Photochemical Transformation

The mechanistic proposal of this intriguing transformation is shown in Scheme 3. X-ray structures secured for selected substrates (i.e., **1b** and **1c**) indicate a H-bond between the amide N–H and one nitrogen atom of the triazine moiety giving a stable 6-membered ring. Upon exposure to light, it is believed that this H-bond is broken, allowing for a conformational change whereby the carbonyl group can position itself nearby the amide moiety. Photoexcitation of this carbonyl is followed by an ISC from the singlet to the more favorable triplet state. The biradical character of this excited carbonyl then enables the key [1,5]-H shift which is facilitated by the prearranged cyclic transition state in **INT-I**. This process is reminiscent of the γ-H abstraction in classical Norrish type II reactions and leads to the formation of a new N–N bond as well as the fragmentation of the acetamide moiety, which can be isolated for these reactions. To the best of our knowledge, the presented case is the first of its kind showcasing a Norrish type II process across three contiguous nitrogen atoms.

**Scheme 3:**
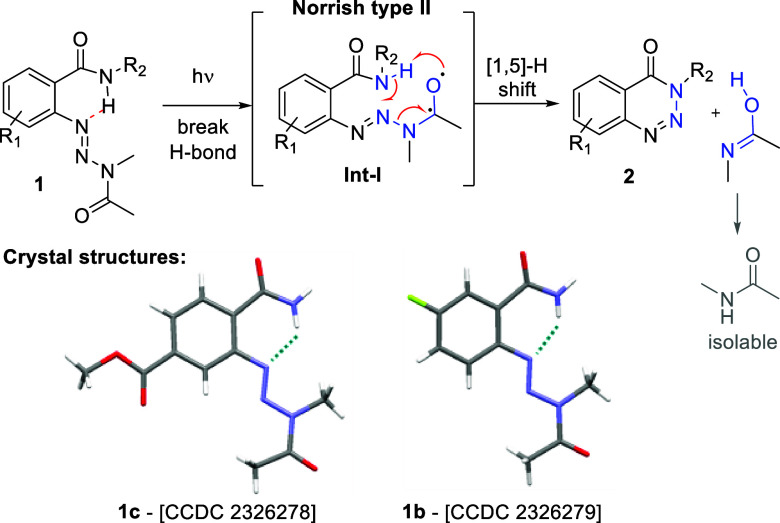
Proposed Mechanism for the Photochemical Benzotriazin-4(3*H*)-one Formation

Lastly, a series of test reactions were performed to glean some further insight into the reaction (Scheme 4). Substrate **1q**, bearing a different acetamide, also afforded benzotriazin-4(3*H*)-one **2a** in almost quantitative yield showing that a bulkier acetamide group is well tolerated. However, when using a triazine substrate lacking the acetamide (i.e., **1r**), no reaction was observed, showing that the acetamide moiety with its carbonyl group is crucial. Equally, the N–H group of the amide is crucial, as in the case of tertiary amides the desired Norrish type II process cannot operate. As seen for substrate **1s** a new product **3** is observed indicating a more complex outcome as photolysis of the acetamide and subsequent denitrification are accompanied by loss of one ethyl group (see SI for details).

**Scheme 4:**
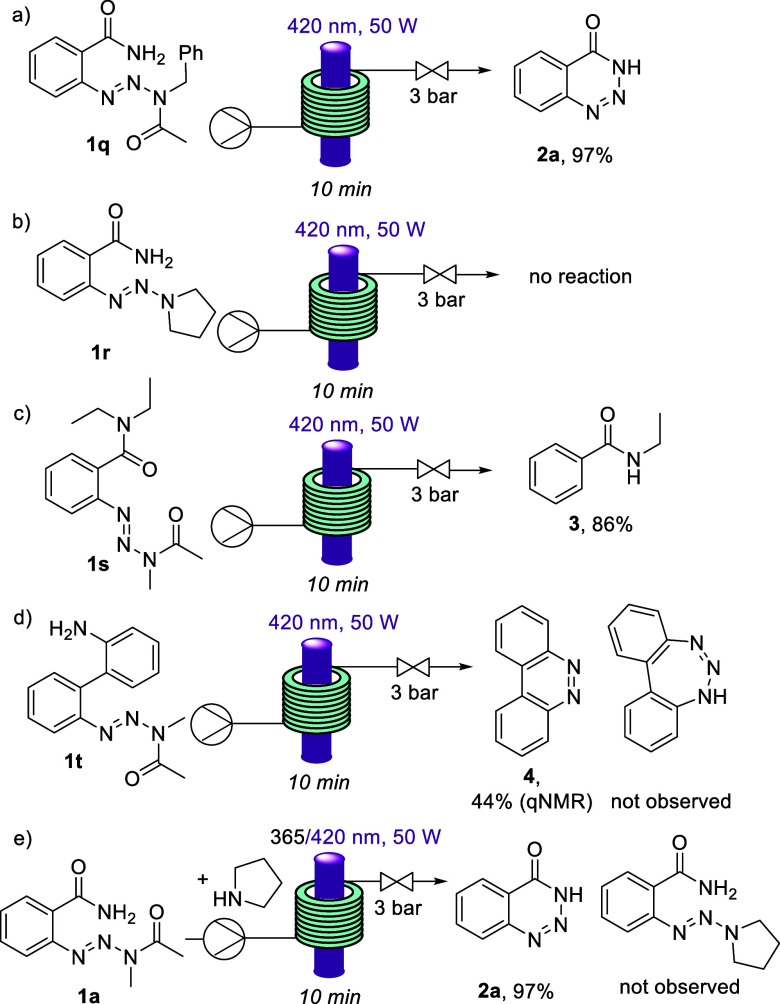
Further Control Experiments

An interesting observation was made when testing the feasibility to generate 7-membered ring products using biphenyl substrate **1t**. Upon irradiation one major product was isolated which matched structure **4** based on comparison with literature data.^16^ This indicates that aromatic 6-membered ring products are favored in this case. Clearly, more complex mechanistic pathways are involved in the formation of products **3** and **4** that extend beyond current knowledge in relation to the reactivity of aryl triazines.^17^ Finally, a competition experiment between the intermolecular triazine formation and the intramolecular reaction described in our previous report was tested; however, in all scenarios tested, the only product observed was benzotriazin-4(3*H*)-one **2a**.

Finally, to demonstrate the scalability and robustness of the flow method, 1 g (4.18 mmol) of substrate **1b** was successfully processed (Scheme 5). Although the isolated yield was slightly lower than in the case of the small-scale reaction (85%), productivity and throughput were very high. Moreover, the green credentials of this flow process^18^ make it a more appealing option compared to reported alternatives. Thus, our protocol shows clear benefits as (1) no additives/catalysts are required; (2) the reaction is fast, uses visible light, and operates at ambient temperature; (3) a miniaturized flow reactor is used that provides for safety and scalability; and (4) the product isolation by simple crystallization avoids chromatography. The only red flag is the use of DCM as cosolvent; however, this can be replaced by a mixture of acetonitrile/water albeit at lower concentration. In contrast to this, previously reported protocols^7−11^ for generating benzotriazin-4(3*H*)-ones use potentially explosive diazonium salts, toxic reagents, harsh conditions, and/or large amounts of additives.

**Scheme 5:**
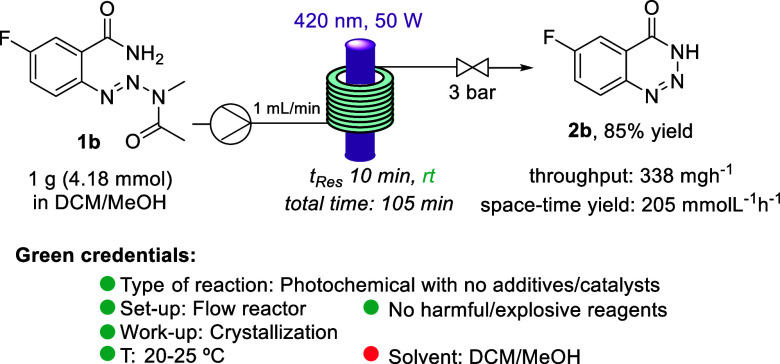
Reaction Scale-up and Green Credentials

In conclusion, we have developed a new method for the synthesis of benzotriazin-4(3*H*)-ones, which is based on the photochemical cyclization of amide bearing aryl triazines. The reaction uses violet light (420 nm) and uniquely operates via a nitrogen-centered [1,5]-H shift which is related to more classical Norrish type II reactions^19^ but unprecedented in this context. Continuous flow processing provides for scalability and process robustness, and the green credentials of this transformation show clear advantages compared to alternative routes toward these underexplored heterocyclic targets. This reaction is characterized by a wide substrate scope affording the desired benzotriazin-4(3*H*)-one products in high chemical yields with *N*-methylacetamide being the sole byproduct.

## Data Availability

The data underlying this study are available in the published article and its Supporting Information.

## Supporting Information Available

Complete experimental procedures, compound characterization, and pictures of the flow equipment. (PDF)
FAIR data, including the primary NMR FID files, for compounds **1b**–**1t**, **2a**–**2p**, **3**–**4** (ZIP)
