# Supplementary material for: Continuous Flow Synthesis of Benzotriazin-4(3H)-ones via Visible Light Mediated Nitrogen-Centered Norrish Reaction
Source: Org Lett. 2024 Mar 11;26(12):2371–5. doi: 10.1021/acs.orglett.4c00248 (PMC10985655; doi:10.1021/acs.orglett.4c00248)

Continuous Flow Synthesis of Benzotriazin-4(3*H*)-ones *via* Visible Light Mediated Nitrogen-Centered Norrish Reaction

Jorge García-Lacuna,* Marcus Baumann*

University College Dublin, School of Chemistry, Science Centre South, Belfield, Dublin 4, Ireland

Email: jorge.garcialacuna@ucd.ie and marcus.baumann@ucd.ie

Table of contents

General materials and methods 3

Experimental procedures 4

Characterization data 6

Characterization data of starting materials (**1a**-**1t**) 6

Characterization data of Benzotriazinones (**2a-2p**) 15

Characterization data of other compounds (**3** and **4**) 21

X-Ray data 23

Pictures of the flow equipment 28

References 29

Copies of NMR data 30

NMR copies of starting materials (**1b**-**1t**) 30

NMR copies of Benzotriazin-4(3*H*)-ones (**2a-2p**) 49

NMR copies of other compounds (**3**-**4**) 68

General materials and methods

Unless otherwise stated, all solvents were purchased from Fisher Scientific and used without further purification. Also, unless otherwise stated, all substrates and reagents were purchased from Fluorochem or Sigma-Aldrich and used as received. ^{1}H NMR spectra were recorded on 400, 500 and 600 MHz instruments and are reported relative to the residual solvent: $CHCl_3$ (δ 7.26 ppm) or DMSO-d6 (δ 2.50 ppm). $^{13}C\{^{1}H\}$ NMR spectra were recorded on the same instruments (100 and 125 MHz) and are reported relative to $CHCl_3$ (δ 77.0 ppm) or DMSO-d6 (δ 39.52 ppm). ^{19}F NMR were recorded at 376 MHz.

Data for ^{1}H NMR are reported as follows: chemical shift (δ/ ppm) (integration, multiplicity, coupling constant (Hz)). Multiplicities are reported as follows: s = singlet, d = doublet, t = triplet, q = quartet, p = pentet, m = multiplet, br s = broad singlet, app = apparent. Data for $^{13}C\{^{1}H\}$ NMR are reported in terms of chemical shift (δ/ppm) and multiplicity (C, CH, CH_2, or CH_3). COSY and HSQC experiments were used in the structural assignment.

High-resolution mass spectrometry was performed using the indicated techniques on a micromass LCT orthogonal time-of-flight mass spectrometer with leucine-enkephalin (Tyr-GlyPhe-Leu) as an internal lock mass. For UV/Vis measurements, a Shimadzu UV-1800 UV spectrophotometer was used. Melting points were recorded with a Stuart SMP10 melting point apparatus and are uncorrected. IR spectra were obtained by use of a Bruker Platinum spectrometer (neat, ATR sampling) with the intensities of the characteristic signals being reported as weak (w, 71% of the tallest signal). signal), medium (m, 21–70% of the tallest signal), or strong (s, >71% of the tallest signal).

Continuous flow experiments were performed on a Vapourtec E-series system equipped with peristaltic pumps and a dynamic BPR achieved through utilization of a peristaltic pump in a reverse direction (1-9 bar, Vapourtec). For photochemical experiments the UV-150 module (Vapourtec) was used in combination with a high-power LED (365 nm or Dual 385-420 nm, purchased from Vapourtec), tuned at the desired W (0-100 Watt) and cooled to 25-30 °C by passing a stream of compressed air through the reactor unit. No filters were used. Reactor coils were made of PFA (Perfluoroalkoxy polymer) tubing (i.d. 1/16 inch) with a volume of 10 mL.

Batch heated reactions were performed using DrySyn heating blocks.

TLC was performed on Merck pre-coated Silica gel 60 F254 aluminium plates with realisation by UV irradiation at 254 nm, $KMnO_4$ and vanillin stain. Flash chromatography was performed using Macherey-Nagel silica gel 60 M, with a particle range of 0.04 - 0.063 mm.

Experimental procedures

- Benzo[*d*][1,2,3]triazin-4(3*H*)-one synthesis optimization

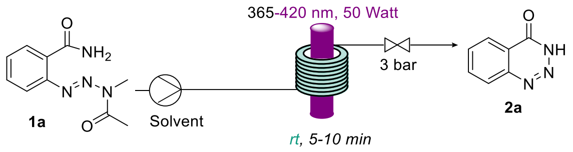

A solution of 66 mg (0.3 mmol) of **1a** in the corresponding degassed solvent is prepared. Once total solubility is achieved, the homogenous solution is placed in the reaction tube inlet and the valve is switched to inject the sample. Beforehand, the flow system is stabilized by setting the light intensity, flow rate, and back pressure (3 bar) for at least 5 minutes. Upon complete injection, the vial is rinsed (1 mL of the solvent) and finally, the valve is switched again to the solvent inlet. The solution is collected at the outlet of the reactor, the solvent is evaporated *in vacuo* and qNMR is calculated using 1,3,5-trimethoxybenzene as internal standard.

- General flow procedure for benzo[*d*][1,2,3]triazin-4(3*H*)-one synthesis

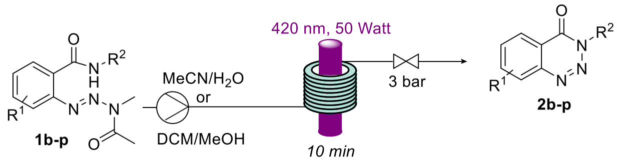

A 0.15-0.3 mmol (40-80 mg) solution of the starting material **1b-p** in the corresponding degassed solvent is prepared. Once total solubility is achieved, the homogenous solution is placed in the reaction tube inlet and the valve is switched to inject the sample. Beforehand, the flow system is stabilized by setting the light intensity at 50 Watt (420 nm wavelength), flow rate at 1 mL/min, and back pressure (3 bar) for at least 5 minutes. A peristaltic pump in a reverse direction was used as dynamic BPR (1-9 bar, Vapourtec) to achieve a constant pressure. Upon complete injection, the vial is rinsed (1 mL of the solvent) and finally, the valve is switched again to the solvent inlet. The solution is collected at the outlet of the reactor after 10 minutes, the solvent is evaporated *in vacuo* and purification is performed using column chromatography unless otherwise stated.

- General protocol for primary amides synthesis: (Starting materials: **1a-1e**, **1q)**

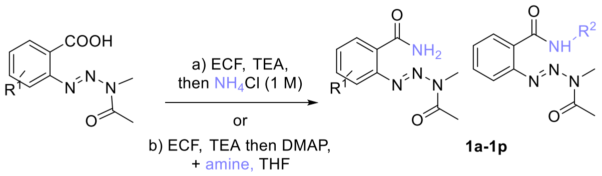

Our previous protocol is followed.[1] To a solution of the acid in THF (0.1 M) and triethylamine (3 equiv.), ethyl chloroformate (1.4 equiv.) is slowly added at 0 °C. After stirring for 10 min at 0 °C, TLC showed total conversion and 2 equiv. of NH_4Cl in a 1 M concentration in water is added at 0 °C. The mixture is stirred for 30 min at 0 °C. Then, the reaction is diluted with water and ethyl acetate. Both phases are separated, and the aqueous layer is extracted with ethyl acetate twice.

Combined organic extracts are washed with brine, dried over sodium sulphate and the solvent evaporated *in vacuo*. The desired amide is purified by flash column chromatography.

- General protocol for secondary amides synthesis: (Starting material synthesis **1f-1p)**

To a solution of the acid in THF (0.1 M) and triethylamine (3 equiv.), ethyl chloroformate (1.4 equiv.) is slowly added at 0 °C. After stirring for 30 min at 0 °C, 1.5 equiv. of the corresponding amine and 0.20 equiv. of dimethylaminopyridine are added in THF (0.5 M) at 0 °C. The mixture is stirred for 30 min at 0 °C, and 14 h at room temperature. Then, the reaction is diluted with water and ethyl acetate. Both phases are separated, and the aqueous layer is extracted with ethyl acetate twice. Combined organic extracts are washed with brine, dried over sodium sulphate and the solvent evaporated *in vacuo*. The desired amide is purified by flash column chromatography.

Characterization data

Characterization data of starting materials (1a-1t)

Compounds **1a**, **1g**, **1f**, **1r** and the carboxylic acids precursors of the amides are described in our previous publication.[1]

1b: (*E*)-2-(3-acetyl-3-methyltriaz-1-en-1-yl)-5-fluorobenzamide

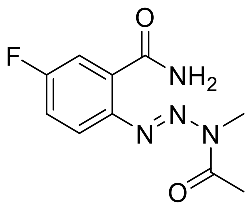

Following the general procedure for primary amides synthesis, using 500 mg (2.09 mmol) of the corresponding carboxylic acid as starting material. Eluent used in the isolation: Gradient from DCM:EtOAc (9:1) to 100% EtOAc: 383 mg (1.61 mmol, 77% yield) were obtained as white crystals. Off-white crystals suitable for X-ray crystallography were obtained by slow evaporation of the solid in chloroform. **M. p.**: 175 – 176 ºC. **^{1}H NMR (500 MHz, CDCl$_3$)** δ 8.16 (bs, 1H, N*H*H), 8.01 (dd, *J* = 9.5, 3.0 Hz, 1H, ArH), 7.65 (dd, *J* = 9.0, 5.0 Hz, 1H, ArH), 7.23 (ddd, *J* = 9.0, 7.1, 3.0 Hz, 1H, ArH), 6.22 (bs, 1H, N*H*H), 3.46 (s, 3H, CH_3N), 2.60 (s, 3H, CH_3). **^{19}F NMR (376 MHz, CDCl$_3$)** δ -110.37 (ddd, *J* = 9.5, 7.1, 5.0 Hz). **^{13}C{^{1}H} NMR (126 MHz, CDCl$_3$)** δ 172.8 (CO), 166.1 (CO), 162.8 (d, *J* = 250.8 Hz, C), 142.5 (d, *J* = 3.3 Hz, C), 131.1 (d, *J* = 7.6 Hz, C), 119.5 (d, *J* = 23.3 Hz, CH), 119.3 (d, *J* = 8.1 Hz, CH), 118.3 (d, *J* = 24.5 Hz, CH), 28.3 (CH_3), 22.1 (CH_3). **HRMS** (ESI-TOF) m/z: $[M+H]^+$ Calcd for $C_{10}H_{12}FN_4O_2$ 239.0939; Found 239.0940.

1c: methyl (*E*)-3-(3-acetyl-3-methyltriaz-1-en-1-yl)-4-carbamoylbenzoate

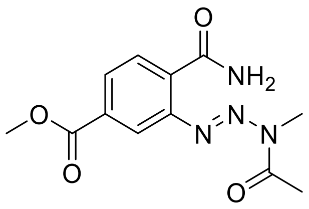

Following the general procedure for primary amides synthesis, using 92 mg (0.329 mmol) of the corresponding carboxylic acid as starting material. Eluent used in the isolation: Gradient from DCM:EtOAc (9:1) to DCM:EtOAc (1:1): 63 mg (0.227 mmol, 69% yield) were obtained as off-white crystals. Off-white crystals suitable for X-ray crystallography were obtained by slow evaporation of the solid in DCM. **M. p.**: 190 – 192 ºC. **^{1}H NMR (500 MHz, CDCl$_3$)** δ 8.34 (d, *J* = 8.2 Hz, 1H, ArH), 8.23 (d, *J* = 1.6 Hz, 1H, ArH), 8.10 (dd, *J* = 8.2, 1.7 Hz, 1H, ArH), 7.99 (s, 1H, bs, N*H*H), 6.16 (s, 1H, bs, N*H*H), 3.97 (s, 3H, CH_3O), 3.48 (s, 3H, CH_3N), 2.65 (s, 3H, CH_3). **^{13}C{^{1}H} NMR (126 MHz, CDCl$_3$)** δ 173.0 (CO), 166.7 (C), 165.9 (CO), 146.2 (C), 133.6 (CH), 132.3 (C), 132.0 (CH), 129.4 (CH), 118.9 (CH), 52.6 (CH_3), 28.4, 28.4 (CH_3), 22.2 (CH_3). **HRMS** (ESI-TOF) m/z: $[M+H]^+$ Calcd for $C_{12}H_{15}N_4O_4$ 278.1088, Found 278.1090. **IR** (neat) v/cm^{-1}: 3368 (w), 3150 (w),

1716 (s), 1673 (s), 1605 (w), 1459 (m), 1405 (m), 1370 (m), 1340 (m), 1308 (m) 1286 (m), 1235 (m), 1172 (m), 1134 (m), 997 (s), 761 (m), 751 (m), 577 (m), 480 (w), 431 (w).

1d: (*E*)-2-(3-acetyl-3-methyltriaz-1-en-1-yl)-5-methoxybenzamide

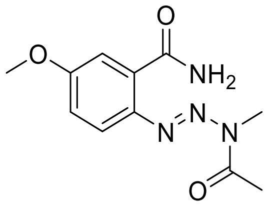

Following the general procedure for primary amides synthesis, using 105 mg (0.338 mmol) of the corresponding carboxylic acid as starting material. Eluent used in the isolation: Gradient from DCM:EtOAc (9:1) to DCM:EtOAc (1:1): 82 mg (0.329 mmol, 75% yield) were obtained as off-white crystals. **M. p.**: 203 – 204 ºC. **^{1}H NMR (500 MHz, CDCl$_3$)** δ 8.28 (bs, 1H, N*H*H), 7.83 (d, *J* = 3.0 Hz, 1H), 7.64 (d, *J* = 9.0 Hz, 1H), 7.07 (dd, *J* = 9.0, 3.0 Hz, 1H), 6.04 (bs, 1H, N*H*H), 3.92 (s, 3H, CH_3O), 3.44 (s, 3H, CH_3N), 2.59 (s, 3H, CH_3). **^{13}C{^{1}H} NMR (126 MHz, CDCl$_3$)** δ 172.9 (CO), 167.2 (CO), 160.4 (C), 139.9 (C), 130.3 (C), 119.8 (CH), 118.7 (CH), 114.4 (CH), 55.8 (CH_3), 28.1 (CH_3), 22.1 (CH_3). **HRMS** (ESI-TOF) m/z: $[M+H]^+$ Calcd for $C_{11}H_{15}N_4O_3$ 251.1139, found 251.1140.

1e: (*E*)-6-(3-acetyl-3-methyltriaz-1-en-1-yl)benzo[*d*][1,3]dioxole-5-carboxamide

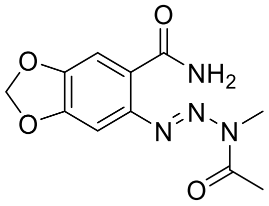

Following the general procedure for primary amides synthesis, using 115 mg (0.434 mmol) of the corresponding carboxylic acid as starting material. Eluent used in the isolation: Gradient from DCM:EtOAc (9:1) to DCM:EtOAc (1:1): 81 mg (0.308 mmol, 71% yield) were obtained as pale yellow crystals. **M. p.**: 220 – 221 ºC. **^{1}H NMR (400 MHz, CDCl$_3$)** δ 8.22 (bs, 1H, N*H*H), 7.78 (s, 1H, ArH), 7.17 (s, 1H, ArH), 6.09 (s, 2H, CH_2), 5.90 (bs, 1H, N*H*H), 3.44 (s, 3H, CH_3N), 2.60 (s, 3H, CH_3). **^{13}C{^{1}H} NMR (101 MHz, CDCl$_3$)** δ 172.8 (CO), 166.7 (CO), 151.1 (C), 148.8 (C), 142.3 (C), 124.6 (C), 110.6 (CH), 102.4 (CH_2), 96.9 (CH), 28.3 (CH_3), 22.2 (CH_3). **HRMS** (ESI-TOF) m/z: $[M+H]^+$ Calcd for $C_{11}H_{13}N_4O_4$ 265.0931; Found 265.0933.

1h: (*E*)-2-(3-acetyl-3-methyltriaz-1-en-1-yl)-*N*-(furan-2-ylmethyl)benzamide

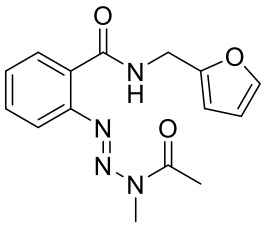

Following the general procedure for secondary amides synthesis, using 120 mg (0.542 mmol) of the corresponding carboxylic acid as starting material and 78 mg (0.81 mmol) of furfurylamine. Eluent used in the isolation: Pentane: Et_2O (1:1): 65 mg (0.216 mmol, 40% yield) were obtained as a white solid. **M. p.**: decomp. **^{1}H NMR (400 MHz, $CDCl_3$)** δ 8.62 (bs, 1H, ArH), 8.37 – 8.31 (m, 1H, ArH), 7.61 – 7.56 (m, 1H, ArH), 7.54 – 7.44 (m, 2H, ArH and CH), 7.36 (dd, *J* = 1.9, 0.9 Hz, 1H, ArH), 6.39 – 6.26 (m, 2H, 2xCH), 4.67 (d, *J* = 4.9 Hz, 2H, CH_2N), 3.20 (s, 3H, CH_3N), 2.58 (s, 3H, CH_3). **^{13}C{^{1}H} NMR (101 MHz, $CDCl_3$)** δ 173.0 (CO), 165.2 (CO), 151.1 (C), 145.8 (C), 142.3 (CH), 131.9 (CH), 131.7 (CH), 129.4 (CH), 129.1 (C), 117.0 (CH), 110.7 (CH), 108.1 (CH), 37.1 (CH_2), 27.8 (CH_3), 22.1 (CH_3). **HRMS** (ESI-TOF) m/z: $[M+H]^+$ Calcd for $C_{15}H_{17}N_4O_3$ 301.1297; Found 301.1295.

1i: (*E*)-2-(3-acetyl-3-methyltriaz-1-en-1-yl)-*N*-(2-methoxybenzyl)benzamide

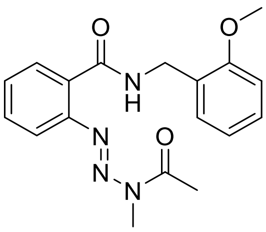

Following the general procedure for secondary amides synthesis, using 100 mg (0.452 mmol) of the corresponding carboxylic acid as starting material and 93 mg (0.68 mmol) of (2-methoxyphenyl)methanamine. Eluent used in the isolation: Pentane: Et_2O (1:1): 66 mg (0.194 mmol, 43% yield) were obtained as a white solid. **M. p.:** (109 – 111 ^oC). **^{1}H NMR (500 MHz, $CDCl_3$)** δ 8.34 – 8.26 (m, 2H, ArH and NH), 7.56 – 7.51 (m, 1H, ArH), 7.50 – 7.43 (m, 2H, ArH), 7.37 (dd, *J* = 7.4, 1.8 Hz, 1H, ArH), 7.30 – 7.25 (m, 1H, ArH), 6.92 (td, *J* = 7.4, 1.1 Hz, 1H, ArH), 6.87 (dd, *J* = 8.2, 1.0 Hz, 1H, ArH), 4.66 (d, *J* = 5.3 Hz, 2H, CH_2), 3.80 (s, 3H, CH_3O), 3.05 (s, 3H, CH_3N), 2.54 (s, 3H, CH_3).). **^{13}C{^{1}H} NMR (101 MHz, $CDCl_3$)** δ 173.0 (CO), 165.4 (CO), 157.5 (C), 145.7 (C), 131.6 (CH), 131.5 (CH), 130.6 (CH), 129.9 (C), 129.3 (CH), 129.2 (CH), 126.2 (C), 120.8 (CH), 117 (CH), 110.4 (CH), 55.3 (CH_3), 39.6 (CH_2), 27.5 (CH_3), 22.1 (CH_3). **HRMS** (ESI-TOF) m/z: $[M+H]^+$ Calcd for $C_{12}H_{14}N_3O_3$ 248.1030; Found 248.1033.

1j: (*E*)-2-(3-acetyl-3-methyltriaz-1-en-1-yl)-*N*-phenylbenzamide

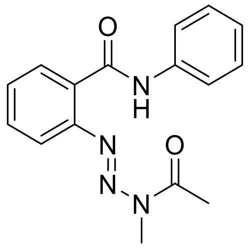

Following the general procedure for secondary amides synthesis, using 100 mg (0.452 mmol) of the corresponding carboxylic acid as starting material and 63 mg (0.68 mmol) of aniline. Eluent used in the isolation: Pentane: Et_2O (1:1): 42 mg (0.140 mmol, 31% yield) were obtained as a white solid. **M. p.**: 152 – 155 ºC. **^{1}H NMR (500 MHz, CDCl$_3$)** δ 10.03 (bs, 1H, NH), 8.40 – 8.35 (m, 1H, ArH), 7.67 – 7.63 (m, 2H, ArH), 7.60 – 7.49 (m, 2H, ArH), 7.42 – 7.35 (m, 2H, ArH), 7.37 – 7.29 (m, 1H, ArH), 7.18 – 7.13 (m, 1H, ArH), 3.57 (s, 3H, CH_3N), 2.63 (s, 3H, CH_3). **^{13}C{^{1}H} NMR (126 MHz, CDCl$_3$)** δ 172.9 (CO), 163.8 (CO), 145.8 (C), 138.1 (C), 132.2 (CH), 131.9 (CH), 129.9 (C), 129.6 (CH), 129.3 (CH), 129.2 (CH), 124.5 (CH), 124.1 (CH), 120.9 (CH), 120.3 (CH), 117.5 (CH), 28.5 (CH_3), 22.1 (CH_3). **HRMS** (ESI-TOF) m/z: $[M+H]^+$ Calcd for $C_{16}H_{17}N_4O_2$ 297.1346; Found 297.1347.

1k: *rac*-methyl (*E*)-(2-(3-acetyl-3-methyltriaz-1-en-1-yl)benzoyl)phenylalaninate

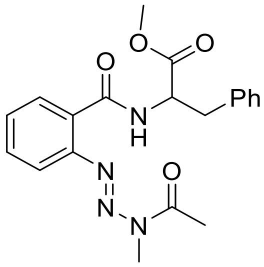

Following the general procedure for secondary amides synthesis, using 50 mg (0.226 mmol) of the corresponding carboxylic acid as starting material and 61 mg (0.34 mmol) of *rac*-methyl phenylalaninate. Eluent used in the isolation: Cyclohexane: AcOEt (3:2): 86 mg (0.140 mmol, 48% yield) were obtained as a white solid. **M. p.**: 123 – 124 ºC. **^{1}H NMR (400 MHz, CDCl$_3$)** δ 8.64 (d, J = 7.5 Hz, 1H, NH), 8.31 – 8.24 (m, 1H, ArH), 7.60 – 7.53 (m, 1H, ArH), 7.53 – 7.43 (m, 2H, ArH), 7.41 – 7.26 (m, 5H, ArH), 5.68 (q, J = 6.9 Hz, 1H, CHN), 3.60 (s, 3H, CH_3O), 3.18 (dd, J = 15.7, 6.2 Hz, 1H, C*H*H), 3.03 (s, 3H, CH_3N), 2.96 (dd, J = 15.7, 7.1 Hz, 1H, C*H*H), 2.55 (s, 3H, CH_3). **^{13}C{^{1}H} NMR (101 MHz, CDCl$_3$)** δ 172.9 (CO), 171.2 (CO), 165.1 (CO), 146.0 (C), 140.4 (C), 131.8 (CH), 131.6 (CH), 129.5 (C), 129.2 (CH), 128.8 (2xCH), 128.0 (CH), 126.7 (2xCH), 117.2 (CH), 51.8 (CH_3), 50.6 (CH), 39.2 (CH_2), 27.8 (CH_3), 22.1 (CH_3). **HRMS** (TOF-ESI+) m/z: $[M+H]^+$ Calcd for $C_{20}H_{23}N_4O_4$ 383.1714; Found 383.1716.

1l: methyl (*E*)-3-(3-acetyl-3-methyltriaz-1-en-1-yl)-4-(cyclopentylcarbamoyl)benzoate

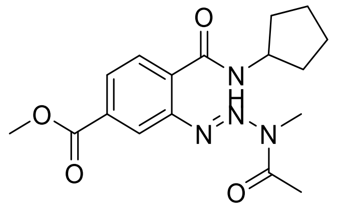

Following the general procedure for secondary amides synthesis, using 100 mg (0.358 mmol) of the corresponding carboxylic acid as starting material and 46 mg (0.537 mmol) of cyclopentylamine. Eluent used in the isolation: Cyclohexane: AcOEt (2:1): 57 mg (0.165 mmol, 46% yield) were obtained as a white solid. **M. p.**: 123 – 124 ºC. **^{1}H NMR (400 MHz, $CDCl_3$)** δ 8.29 (dd, *J* = 8.2, 0.5 Hz, 1H, ArH), 8.18 (dd, *J* = 1.7, 0.5 Hz, 1H, ArH), 8.07 (dd, *J* = 8.2, 1.7 Hz, 1H, ArH), 7.94 (d, *J* = 7.1 Hz, 1H, NH), 4.42 (h, *J* = 7.1 Hz, 1H, CH), 3.95 (s, 3H, CH_3O), 3.47 (s, 3H, CH_3N), 2.63 (s, 3H, CH_3), 2.19 – 2.07 (m, 2H, CH_2), 1.75 – 1.61 (m, 4H, 2xCH_2), 1.51 – 1.39 (m, 2H, CH_2). **$^{13}C\{^1H\}$ NMR (101 MHz, $CDCl_3$)** δ 173.0 (CO), 165.9 (CO), 164.5 (CO), 145.9 (C), 133.6 (C), 132.8 (C), 131.7 (CH), 129.5 (CH), 118.8 (CH), 52.6 (CH), 51.8 (CH_3), 33.3 (2xCH_2), 28.3 (CH_3), 23.9 (2xCH_2), 22.2 (CH_3). **HRMS** (TOF-ESI+) m/z: $[M+H]^+$ Calcd for $C_{17}H_{23}N_4O_4$ 347.1714; Found 347.1717.

1m: (*E*)-6-(3-acetyl-3-methyltriaz-1-en-1-yl)-*N*-isobutylbenzo[*d*][1,3]dioxole-5-carboxamide

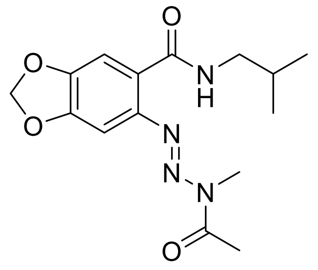

Following the general procedure for secondary amides synthesis, using 100 mg (0.377 mmol) of the corresponding carboxylic acid as starting material and 41 mg (0.565 mmol) of isobutylamine. Eluent used in the isolation: Cyclohexane: AcOEt (2:1): 54 mg (0.170 mmol, 45% yield) were obtained as pale-yellow crystals. **M. p.**: 160 – 162 ºC. **^{1}H NMR (400 MHz, $CDCl_3$)** δ 8.29 (bs, 1H, NH), 7.77 (s, 1H, ArH), 7.14 (s, 1H, ArH), 6.07 (s, 2H, CH_2O), 3.43 (s, 3H, CH_3N), 3.30 (dd, *J* = 7.0, 6.0 Hz, 2H, CH_2N), 2.59 (s, 3H, CH_3), 1.86 (dt, *J* = 13.5, 6.7 Hz, 1H, CH), 0.96 (d, *J* = 6.7 Hz, 6H, 2xCH_3). **$^{13}C\{^1H\}$ NMR (101 MHz, $CDCl_3$)** δ 172.8 (CO), 165.0 (CO), 150.4 (C), 148.8 (C), 141.8 (C), 125.8 (C), 110.5 (CH), 102.3 (CH_2), 96.8 (CH), 47.8 (CH_2), 28.7 (CH_2), 28.2 (CH_3), 22.2 (CH_3), 20.4 (2xCH_3). **HRMS** (ESI-TOF) m/z: $[M+H]^+$ Calcd for $C_{15}H_{21}N_4O_4$ 321.1557; Found 321.1561. **IR** (neat) v/cm^{-1}: 3295 (w), 1703 (s), 1631 (w), 1612 (w), 1595 (w), 1542 (m), 1503 (m), 1482 (m), 1416 (m), 1369 (w), 1343 (w), 1312 (m), 1282 (w), 1207 (w), 1166 (m), 1144 (w), 1124 (w), 1064 (m), 1039 (s), 960 (w), 684 (w), 655 (w), 586 (m) 567 (m), 515 (w), 489 (w) 462 (w).

1n: (*E*)-2-(3-acetyl-3-methyltriaz-1-en-1-yl)-*N*-benzyl-5-fluorobenzamide

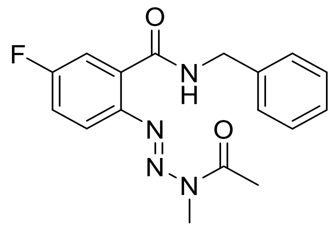

Following the general procedure for secondary amides synthesis, using 100 mg (0.418 mmol) of the corresponding carboxylic acid as starting material and 67 mg (0.452 mmol) of benzylamine. Eluent used in the isolation: Cyclohexane: AcOEt (3:1): 47 mg (0.142 mmol, 32% yield) were obtained as an off-white solid. **M. p.**: 155 – 157 ºC. **^{1}H NMR (400 MHz, CDCl$_3$)** δ 8.62 (bs, 1H, NH), 8.08 (dd, *J* = 9.7, 3.0 Hz, 1H, ArH), 7.61 (dd, *J* = 9.0, 5.1 Hz, 1H, ArH), 7.39 – 7.27 (m, 5H, ArH), 7.19 (ddd, *J* = 9.0, 7.2, 3.0 Hz, 1H, ArH), 4.64 (d, *J* = 5.0 Hz, 2H, CH_2), 2.84 (s, 3H, CH_3), 2.53 (s, 3H, CH_3). **^{13}C{^{1}H} NMR (101 MHz, CDCl$_3$)** δ 172.7 (CO), 163.9 (d, *J* = 1.9 Hz, CO), 162.9 (d, *J* = 250.6 Hz, C), 142.2 (d, *J* = 3.1 Hz, C), 137.7 (C)k, 131.5 (d, *J* = 7.5 Hz, C), 128.9 (2xCH), 128.5 (2xCH), 128.0 (CH), 119.1 (d, *J* = 11.2 Hz, CH), 118.9 (d, *J* = 3.9 Hz, CH), 118.2 (d, *J* = 24.8 Hz, CH), 44.9 (CH_2), 27.7 (CH_3), 22.0 (CH_3). **^{19}F NMR (376 MHz, CDCl$_3$)** δ -110.43 (ddd, *J* = 9.7, 7.2, 5.1 Hz). **HRMS** (TOF-ESI+) m/z: $[M+Na]^+$ Calcd for $C_{17}H_{17}FN_4O_2Na$ 351.1228; Found 351.1228.

1o: methyl (*E*)-3-(3-acetyl-3-methyltriaz-1-en-1-yl)-4-((4-isopropylphenyl)carbamoyl)benzoate

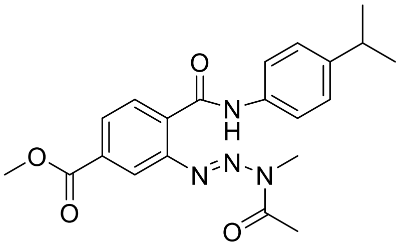

Following the general procedure for secondary amides synthesis, using 120 mg (0.430 mmol) of the corresponding carboxylic acid as starting material and 87 mg (0.644 mmol) of 4-isopropylaniline. Eluent used in the isolation: Cyclohexane: AcOEt (3:1): 42 mg (0.107 mmol, 25% yield) were obtained as a white solid. **M. p.**: 141 – 143 ºC. **^{1}H NMR (400 MHz, CDCl$_3$)** δ 9.96 (s, 1H, NH), 8.42 (dd, *J* = 8.2, 1H, ArH), 8.26 (d, *J* = 1.3 Hz, 1H, ArH), 8.13 (dd, *J* = 8.2, 1.7 Hz, 1H, ArH), 7.60 – 7.51 (m, 2H, ArH), 7.28 – 7.21 (m, 3H, ArH), 3.57 (s, 3H, CH_3), 2.94 – 2.85 (m, 1H, CH), 2.66 (s, 3H, CH_3), 1.25 (d, *J* = 6.9 Hz, 6H, 2xCH_3). **^{13}C{^{1}H} NMR (101 MHz, CDCl$_3$)** δ 172.9 (CO), 165.9 (CO), 162.7 (CO), 145.8 (C), 145.6 (C), 135.5 (C), 133.4 (CH), 132.1 (CH), 129.6 (CH), 127.2 (2xCH), 120.4 (2xCH), 119.0 (CH), 52.6 (CH_3), 33.6 (CH), 28.7 (CH_3), 24.0 (2xCH_3), 22.2 (CH_3). **HRMS** (ESI-TOF) m/z: $[M+H]^+$ Calcd for $C_{21}H_{25}N_4O_4$ 397.1870; Found 397.1872.

1p: (*E*)-6-(3-acetyl-3-methyltriaz-1-en-1-yl)-*N*-(4-fluorophenyl)benzo[*d*][1,3]dioxole-5-carboxamide

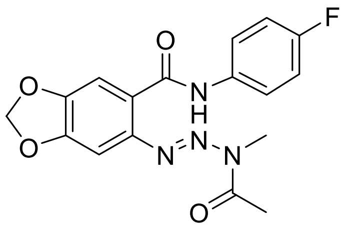

Following the general procedure for secondary amides synthesis, using 120 mg (0.452 mmol) of the corresponding carboxylic acid as starting material and 75 mg (0.678 mmol) of 4-flourolaniline. Eluent used in the isolation: Cyclohexane: AcOEt (1:1): 37 mg (0.104 mmol, 23% yield) were obtained as a pale-yellow solid. **M. p.**: decomp. **^{1}H NMR (500 MHz, CDCl$_3$)** δ 10.24 (bs, 1H, NH), 7.85 (s, 1H, ArH), 7.58 (dd, *J* = 9.0, 4.8 Hz, 2H, ArH), 7.20 (s, 1H, ArH), 7.06 (dd, *J* = 9.1, 8.3 Hz, 2H, ArH), 6.11 (s, 2H, CH_2), 3.54 (s, 3H, CH_3), 2.62 (s, 3H, CH_3). **^{13}C{^{1}H} NMR (126 MHz, CDCl$_3$)** δ 172.78 (CO), 163.0 (CO), 159.42 (d, *J* = 244.0 Hz, CH), 151.1 (C), 149.1 (C), 141.9 (C), 134.18 (d, *J* = 2.9 Hz, C), 125.3 (C), 122.09 (d, *J* = 7.7 Hz, 2xCH), 115.83 (d, *J* = 22.4 Hz, 2xCH), 110.5 (CH), 102.5 (CH_2), 97.0 (CH), 28.6 (CH_3), 22.2 (CH_3). **^{19}F NMR (376 MHz, CDCl$_3$)** δ -117.72 (ddd, *J* = 8.3, 4.8, 3.5 Hz). **HRMS** (ESI-TOF) m/z: $[M+H]^+$ Calcd for $C_{17}H_{15}FN_4O_4$ 359.1150; Found 359.1150.

1q: (*E*)-2-(3-acetyl-3-benzyltriaz-1-en-1-yl)benzamide

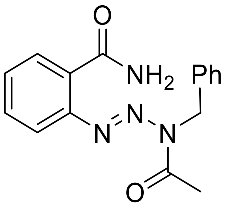

Following the general procedure for primary amides synthesis, using 83 mg (0.279 mmol) of the corresponding carboxylic acid as starting material. Eluent used in the isolation: DCM:EtOAc (9:1) 55 mg (0.184 mmol, 66% yield) as a white solid. **M. p.**: 179 – 191 ºC. **^{1}H NMR (400 MHz, CDCl$_3$)** δ 8.18 (ddd, *J* = 7.8, 1.6, 0.6 Hz, 1H, ArH), 7.55 – 7.41 (m, 3H, ArH), 7.36 – 7.24 (m, 3H, ArH), 7.19 – 7.09 (m, 2H, ArH), 6.76 (bs, 1H, N*H*H), 5.36 (s, 2H, CH_2), 5.20 (bs, 1H, N*H*H), 2.73 (s, 3H, CH_3). **^{13}C{^{1}H} NMR (101 MHz, CDCl$_3$)** δ 173.3 (CO), 167.0 (CO), 146.2 (C), 134.7 (C), 132.1 (CH), 131.7 (CH), 129.3 (CH), 128.99 (2xCH_2), 128.90 (C), 127.6 (CH), 126.1 (2xCH), 117.8 (CH), 44.7 (CH_2), 22.3 (CH_3). **HRMS** (TOF-ESI+) m/z: $[M+H]^+$ Calcd for $C_{16}H_{17}N_4O_2$ 297.1346; Found 297.1345.

1s: (*E*)-2-(3-acetyl-3-methyltriaz-1-en-1-yl)-*N*,*N*-diethylbenzamide

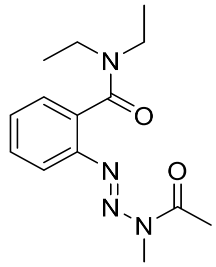

Following the general procedure for secondary amides synthesis, using 100 mg (0.452 mmol) of the corresponding carboxylic acid as starting material and 50 mg (0.68 mmol) of diethylamine. Eluent used in the isolation: Gradient from Cyclohexane: AcOEt (3:1) to (1:3). 59 mg (0.213 mmol, 47% yield) were obtained as a colorless oil. **^{1}H NMR (400 MHz, CDCl$_3$)** δ 7.59 – 7.54 (m, 1H, ArH), 7.44 – 7.34 (m, 3H, ArH), 3.83 (bs, 1H, C*H*H), 3.36 (s, 3H, CH_3N), 3.35 (bs, 1H, C*H*H), 3.14 (bs, 2H, CH_2), 2.54 (s, 3H, CH_3CO), 1.24 (t, *J* = 7.1 Hz, 3H, CH_3), 1.01 (t, *J* = 7.1 Hz, 3H, CH_3). **^{13}C{^{1}H} NMR (101 MHz, CDCl$_3$)** δ 173.3 (CO), 169.0 (CO), 144.5 (C), 135.3 (C), 129.3 (CH), 129.2 (CH), 127.1 (CH), 117.4 (CH), 42.8 (CH_2), 38.8 (CH_2), 27.6 (CH_3), 22.0 (CH_3), 13.9kk (CH_3), 12.9 (CH_3). **HRMS** (ESI-TOF) m/z: $[M+H]^+$ Calcd for $C_{14}H_{21}N_4O_2$ 277.1659; Found 277.1660.

1t: (*E*)-1-(3-(2'-amino-[1,1'-biphenyl]-2-yl)-1-methyltriaz-2-en-1-yl)ethan-1-one

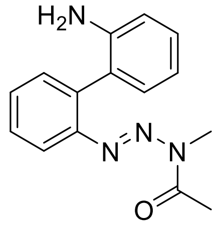

Yellow oil. **^{1}H NMR (400 MHz, CDCl$_3$)** δ 7.59 – 7.51 (m, 1H, ArH), 7.47 – 7.40 (m, 3H, ArH), 7.15 (ddd, *J* = 8.0, 7.3, 1.6 Hz, 1H, ArH), 7.06 (dd, *J* = 7.6, 1.6 Hz, 1H, ArH), 6.79 (td, *J* = 7.4, 1.2 Hz, 1H, ArH), 6.73 (dd, *J* = 8.0, 1.1 Hz, 1H, ArH), 3.59 (bs, 2H, NH_2), 3.16 (s, 3H, CH_3), 2.42 (s, 3H, CH_3). **^{13}C{^{1}H} NMR (101 MHz, CDCl$_3$)** δ 173.3 (CO), 146.7 (C), 143.6 (C), 135.8 (C), 131.7 (CH), 131.1 (CH), 129.2 (CH), 128.5 (CH), 128.5 (CH), 125.3 (C), 118.6 (CH), 118.2 (CH), 115.3 (CH), 27.4 (CH_3), 21.9 (CH_3). **HRMS** (ESI-TOF) m/z: $[M+H]^+$ Calcd for $C_{15}H_{17}N_4O$ 269.1397; Found 269.1397.

The title compound was synthesized according to the following scheme:

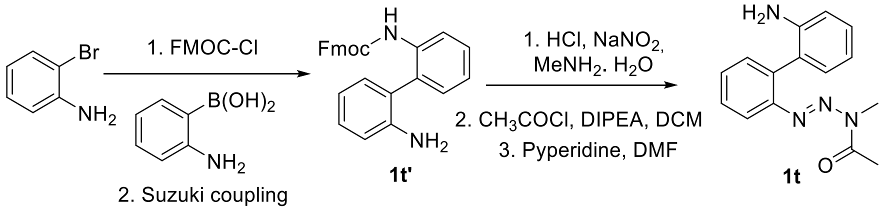

To 596 mg (3.46 mmol) of 2-bromoaniline in a round bottom flask, 1 g of fmoc-cl (3.46 mmol) was added with few water drops. Both solids were gently stirred at 110 ºC for 4 h. Then, the reaction was allowed to cool down to room temperature and 10 mL of 0.1 M HCl aq. solution were added.

The crude was extracted with EtOAc (20 mL) three times. Combined organic layers were washed with brine and dried with sodium sulfate. No further purification was needed.

Suzuki coupling: 300 mg (0.76 mmol) of the resulting protected product was added to a flame dried round bottom flask with the boronic acid (156 mg, 1.14 mmol, 1.5 equiv.), potassium carbonate (422 mg, 3.05 mmol, 4 equiv.) and PPh_3 (40 mg, 0.15 mmol, 0.2 equiv.) in 41 mL of toluene/EtOH (3/1). The suspension is degassed three times with nitrogen gas, and $Pd(Ph_3)_2Cl_2$ (53 mg, 0.08 mmol, 0.1 equiv.), and 3.5 mL of H_2O were added. The suspension is refluxed at 120 ºC for 4 hours. After that time, TLC showed no starting material, and the crude products was filtered though a pad of zelite. Solvent was evaporated *in vacuo* and product **1t'** was obtained as a pale yellow foam after column chromatography (Cyclohexane:AcOEt, gradient from 12:1 to 4:1). 42% yield (130 mg, 0.319 mmol)

1t': (9*H*-fluoren-9-yl)methyl (2'-amino-[1,1'-biphenyl]-2-yl)carbamate

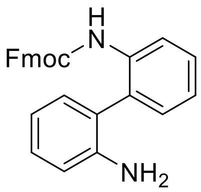

Pale yellow foam. **M. p.**: 66 – 67 ºC. **^{1}H NMR (500 MHz, $CDCl_3$)** δ 7.99 (bs, 1H, NH), 7.78 – 7.73 (m, 2H, ArH), 7.53 – 7.46 (m, 2H, ArH), 7.41 – 7.38 (m, 3H, ArH), 7.31 – 7.26 (m, 4H, ArH), 7.19 (td, *J* = 7.5, 1.2 Hz, 1H, ArH), 7.12 (dd, *J* = 7.6, 1.6 Hz, 1H, ArH), 7.01 (bs, 1H, ArH), 6.91 (td, *J* = 7.4, 1.2 Hz, 1H, ArH), 6.85 (dd, *J* = 8.0, 1.2 Hz, 1H, ArH), 4.47 – 4.41 (m, 1H, C*H*HO), 4.38 – 4.32 (m, 1H, C*H*HO), 4.24 (t, *J* = 7.3 Hz, 1H, C*H*), 3.60 (bs, 2H, NH_2). **^{13}C NMR (126 MHz, $CDCl_3$)** δ 153.8 (CO), 143.8 (C), 143.72 (C), 143.70 (C), 141.29 (C), 141.28 (C), 135.6 (C), 131.1 (CH), 130.7 (CH), 129.5 (CH), 128.8 (CH), 127.70 (CH), 127.69 (CH), 127.0 (3xCH), 125.2 (CH), 125.1 (CH), 124.2 (C),123.3 (C), 120.0 (2xCH), 119.2 (2xCH), 115.7 (CH), 67.0 (CH_2), 47.0 (CH). **HRMS** (ESI-TOF) m/z: $[M+H]^+$ Calcd for $C_{27}H_{23}N_2O_2$ 406.1682; Found 406.1685.

To a solution of 0.3 mL (3.66 mmol, 6 equiv.) HCl (cc), **1t'** (250 mg, 0.61 mmol) was slowly added at 0 ºC. Then, $NaNO_2$ (43 mg ,0.63 mmol, 1.03 equiv.) in 0.4 mL H_2O was added dropwise and the suspension turned to reddish. After 20 minutes of vigorous stirring in an ice-bath, the suspension was slowly added to a stirred solution of methylamine (0,73 mL, 9.15 mmol, 15 equiv.) in H_2O (40%) at -10 ºC. After complete addition, it was extracted with EtOAc (20 mL x 3). Combined organic layers were washed with brine and dried with sodium sulfate. Solvent was evaporated *in vacuo*. The resulting crude was dissolved in DCM (2.5 mL) and *N,N* -Diisopropylethylenediamine (0.43 mL, 2.44 mmol, 4 equiv.). Acetyl chloride (46 µL,0.64 mmol, 1.05 equiv.) was slowly added in an ice bath. After 3 h, 5 mL of HCl (1M) were added, and the aqueous layer was extracted with DCM (10 mL x3). Combined organic layers were washed with brine and dried with sodium sulfate. Solvent was evaporated *in vacuo.*

The resulting crude was directly dissolved in DMF (4 mL) and piperidine (120 µL, 1.22 mmol, 2 equiv.) was added at room temperature. After 1 h stirring at room temperature TLC showed total conversion. The crude was added to a mixture of ice-water and it was extracted tree times with 10 mL of AcOEt. Combined organic layers were washed with brine and dried with sodium sulfate. Solvent was evaporated *in vacuo.* Product **1t** was purified by column chromatography using Pentane:AcOEt 3:2. 54 mg (0.20 mmol) were obtained as yellow oil (33% yield of the 3 steps)

Characterization data of Benzotriazinones (2a-2p)

2a: benzo[*d*][1,2,3]triazin-4(3*H*)-one

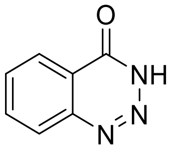

Entry 4 and 7, table 1: Following the general flow procedure, but using 365 nm wavelength. Using 66 mg (0.300 mmol) of starting material **1a** in 4.2 mL (0.05 M) of DCM/MeOH (1:3) or in 7.9 mL of MeCN/H_2O (2/1). 40 mg of the title compound (90% yield, 0.271 mmol) were obtained as a white solid after chromatography. Eluent used in the isolation: DCM: AcOEt (1:1). **^{1}H NMR (400 MHz, $CDCl_3$)** δ 11.95 (bs, 1H, NH), 8.38 (ddd, *J* = 7.9, 1.5, 0.6 Hz, 1H, ArH), 8.22 (ddd, *J* = 8.1, 1.1, 0.6 Hz, 1H, ArH), 8.01 (ddd, *J* = 8.1, 7.3, 1.5 Hz, 1H, ArH), 7.84 (ddd, *J* = 7.9, 7.3, 1.2 Hz, 1H, ArH). The reported data is in accordance with literature.[2]

Entry 8, table 1: 88% of isolated yield (118 mg, 0.801 mmol) was obtained at 200 mg scale (0.91 mmol) of **1a** following the general flow procedure. The product was purified by solvent evaporation followed by redissolving the solid in DCM (5 mL) and the organic layer was washed twice with brine (5 mL) and dried with sodium sulphate.

2b: 6-fluorobenzo[*d*][1,2,3]triazin-4(3*H*)-one

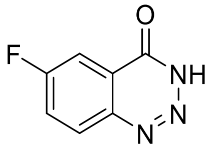

Small scale conditions: Following the general flow procedure, using 50 mg (0.210 mmol) of starting material **1b** in 4.2 mL (0.05 M) of DCM/MeOH (1:3). 33.5 mg of the title compound (97% yield, 0.204 mmol) were obtained as a white solid after chromatography. Eluent used in the isolation: DCM: AcOEt (1:1). **^{1}H NMR (400 MHz, $CDCl_3$)** δ 11.74 (bs, 1H, NH), 8.24 (ddd, *J* = 8.9, 4.8, 0.4 Hz, 1H, ArH), 7.98 (ddd, *J* = 7.7, 2.8, 0.4 Hz, 1H, ArH), 7.68 (ddd, *J* = 8.9, 8.0, 2.8 Hz, 1H, ArH). The reported data is in accordance with literature.[2]

1 g long run conditions: 1 g (4.20 mmol) of **1b** was dissolved in 84 mL of degassed DCM/MeOH (1:3). Once total solubility is achieved, the solution is placed at the reagent inlet. The flow system was previously stabilized for 10 minutes with the following conditions: 1 mL/min of flow rate, bpr at 3 bar, light intensity at 50 W (420 nm), and temperature around 22-25 ºC. Then, the valved is

switched to the reagent line. The crude was collected after 10 minutes. When all the solution was injected into the system, the flask was rinsed with 10 mL of DCM. Total time collecting: 105 min. After total collection the solvent is evaporated in vacuo and the product was purified by crystallization using MeOH. 590 mg of the pure compound were obtained (85% yield, throughput: 338 mg/h, space time yield: 205 mmol/Lh).

2c: methyl 4-oxo-3,4-dihydrobenzo[*d*][1,2,3]triazine-7-carboxylate

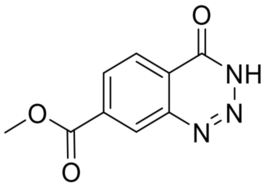

Following the general flow procedure, using 45 mg (0.162 mmol) of starting material **1c** in 4.25 mL (0.038 M) of DCM/MeOH (1:3). 31.5 mg of the title compound (95% yield, 0.154 mmol) were obtained as a white solid after chromatography. Eluent used in the isolation: DCM: AcOEt (9:1). **M. p.:** 202 – 204 ºC. **^{1}H NMR (500 MHz, DMSO-*d*$_6$)** δ 8.60 (dd, *J* = 1.4, 0.8 Hz, 1H, ArH), 8.36 – 8.32 (m, 2H, ArH), 3.97 (s, 3H, CH_3), 3.30 (bs, 1H, NH). **^{13}C{^{1}H} NMR (126 MHz, DMSO-*d*$_6$)** δ 165.2 (CO), 155.5 (CO), 144.5 (C), 136.1 (C), 132.2 (CH), 129.2 (CH), 125.9 (CH), 123.8 (C), 53.5 (CH_3). **HRMS** (ESI-TOF) m/z: $[M+H]^+$ Calcd for $C_9H_8N_3O_3$ 206.0560; Found 206.0562. **IR** (neat) v/cm^{-1}: 3237 (w), 3202 (w), 1703 (s), 1438 (w), 1380 (w), 1316 (m), 1293 (w), 1208 (m), 1134 (m), 1084 (w), 944 (w), 791 (w), 763 (m).

2d: 6-methoxybenzo[*d*][1,2,3]triazin-4(3*H*)-one

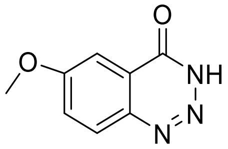

Following the general flow procedure, using 45 mg (0.180 mmol) of starting material **1d** in 4.70 mL (0.038 M) of DCM/MeOH (1:3). 29.3 mg of the title compound (92% yield, 0.164 mmol) were obtained as a white solid after chromatography. Eluent used in the isolation: DCM: AcOEt (9:1). **^{1}H NMR (400 MHz, DMSO-*d*$_6$)** δ 8.13 (d, *J* = 8.9 Hz, 1H, ArH), 7.63 (dd, *J* = 8.9, 2.8 Hz, 1H, ArH), 7.56 (d, *J* = 2.9 Hz, 1H, ArH), 3.97 (s, 3H, CH_3). The reported data is in accordance with literature.[3]

2e: [1,3]dioxolo[4',5':4,5]benzo[1,2-*d*][1,2,3]triazin-4(3*H*)-one

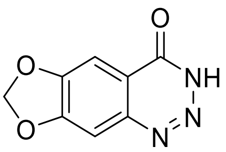

Following the general flow procedure, using 45 mg (0.170 mmol) of starting material **1e** in 6.80 mL (0.025 M) of MeCN/H_2O (2:1). 26 mg of the title compound (80% yield, 0.136 mmol) were

obtained as a white solid after chromatography. Eluent used in the isolation: DCM: AcOEt (1:1). **M. p.:** 199 – 201 ºC. **^{1}H NMR (500 MHz, DMSO-*d*$_6$)** δ 7.61 (s, 1H, ArH), 7.50 (s, 1H, ArH), 6.33 (s, 2H, CH_2). **^{13}C{^{1}H} NMR (126 MHz, DMSO-*d*$_6$)** δ 155.2 (CO), 153.6 (C), 151.3 (C), 142.3 (C), 116.6 (C), 105.6 (CH), 103.5 (CH), 100.7 (CH_2). **HRMS** (ESI-TOF) m/z: $[M+H]^+$ Calcd for $C_8H_6N_3O_3$ 192.0404; Found 192.0406.

2f: 3-cyclopentylbenzo[*d*][1,2,3]triazin-4(3*H*)-one

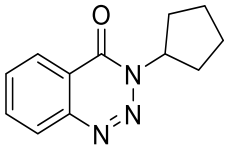

Following the general flow procedure, using 50 mg (0.173 mmol) of starting material **1f** in 3.5 mL (0.05 M) of DCM/MeOH (1:3). 28 mg of the title compound (75% yield, 0.130 mmol) were obtained as a white solid after chromatography. Eluent used in the isolation: Pentane: AcOEt (4:1). **^{1}H NMR (400 MHz, $CDCl_3$)** δ 8.34 (ddd, *J* = 8.0, 1.5, 0.6 Hz, 1H, ArH), 8.13 (ddd, *J* = 8.2, 1.2, 0.6 Hz, 1H, ArH), 7.92 (ddd, *J* = 8.2, 7.2, 1.5 Hz, 1H, ArH), 7.77 (ddd, *J* = 8.2, 7.2, 1.2 Hz, 1H, ArH), 5.53 (tt, *J* = 8.1, 6.9 Hz, 1H, CH), 2.26 – 2.06 (m, 4H), 2.04 – 1.94 (m, 2H), 1.82 – 1.72 (m, 2H). The reported data is in accordance with literature.[4]

2g: 3-isobutylbenzo[*d*][1,2,3]triazin-4(3*H*)-one

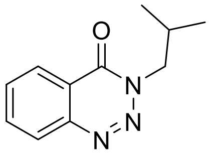

Following the general flow procedure, using 75 mg (0.271 mmol) of starting material **1h** in 5.4 mL (0.05 M) of DCM/MeOH (1:3). 53 mg of the title compound (97% yield, 0.263 mmol) as were obtained a colorless oil after chromatography. Eluent used in the isolation: DCM: AcOEt (9:1).**^{1}H NMR (400 MHz, $CDCl_3$)** δ 8.36 (ddd, *J* = 7.9, 1.5, 0.6 Hz, 1H, ArH), 8.15 (ddd, *J* = 8.2, 1.2, 0.6 Hz, 1H, ArH), 7.94 (ddd, *J* = 8.2, 7.2, 1.5 Hz, 1H, ArH), 7.80 (ddd, *J* = 7.9, 7.2, 1.2 Hz, 1H, ArH), 4.30 (d, *J* = 7.4 Hz, 2H, CH_2), 2.45 – 2.31 (m, 1H, CH), 1.01 (d, *J* = 6.7 Hz, 6H, 2xCH_3). **^{13}C{^{1}H} NMR (101 MHz, $CDCl_3$)** δ 155.7 (CO), 144.2 (C), 134.7 (CH), 132.2 (CH), 128.2 (CH), 125.2 (CH), 119.8 (C), 56.7 (CH_2), 28.2 (CH), 20.0 (2xCH_3). **HRMS** (ESI-TOF) m/z: $[M+H]^+$ Calcd for $C_{11}H_{14}N_3O$ 204.1131; Found 204.1135.

2h: 3-(furan-2-ylmethyl)benzo[*d*][1,2,3]triazin-4(3*H*)-one

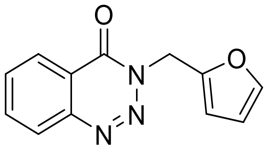

Following the general flow procedure, using 45 mg (0.150 mmol) of starting material **1h** in 3.9 mL (0.038 M) of MeCN/H_2O (2:1). 25.5 mg of the title compound (75% yield, 0.113 mmol) were obtained as a white solid after chromatography. Eluent used in the isolation: Pentane: Et_2O (1:1).**^{1}H NMR (400 MHz, $CDCl_3$)** δ 8.36 (ddd, *J* = 8.0, 1.5, 0.6 Hz, 1H, ArH), 8.16 (ddd, *J* = 8.1, 1.2, 0.6 Hz, 1H, ArH), 7.94 (ddd, *J* = 8.1, 7.2, 1.5 Hz, 1H, ArH), 7.79 (ddd, *J* = 7.9, 7.3, 1.2 Hz, 1H, ArH), 7.37 (dd, *J* = 1.9, 0.9 Hz, 1H, CH), 6.52 – 6.47 (m, 1H, CH), 6.37 – 6.31 (m, 1H, CH), 5.65 – 5.63 (m, 2H, CH_2). The reported data is in accordance with literature.[5]

2i: 3-(2-methoxybenzyl)benzo[*d*][1,2,3]triazin-4(3*H*)-one

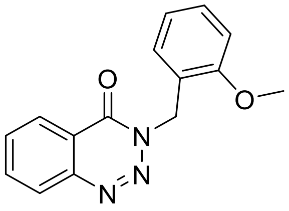

Following the general flow procedure, using 45 mg (0.132 mmol) of starting material **1h** in 2.65 mL (0.05 M) of DCM/MeOH (1:3). 31.5 mg of the title compound (89% yield, 0.117 mmol) as a white solid. The product was purified by solvent evaporation followed by redissolving the solid in DCM (5 mL) and the organic layer was washed twice with brine (5 mL) and dried with sodium sulphate. **M. p.**: 117 – 118 ºC. **^{1}H NMR (400 MHz, $CDCl_3$)** δ 8.36 (ddd, *J* = 8.0, 1.5, 0.6 Hz, 1H, ArH), 8.15 (ddd, *J* = 8.2, 1.2, 0.6 Hz, 1H, ArH), 7.94 (ddd, *J* = 8.2, 7.3, 1.5 Hz, 1H, ArH), 7.78 (ddd, *J* = 7.9, 7.3, 1.2 Hz, 1H, ArH), 7.23 – 7.18 (m, 2H, ArH), 6.94 – 6.86 (m, 2H, ArH), 5.69 (s, 2H, CH_2), 3.87 (s, 3H, CH_3). **^{13}C{^{1}H} NMR (101 MHz, $CDCl_3$)** δ 157.4 (C), 155.5 (C), 144.3 (C), 134.7 (CH), 132.2 (CH), 129.4 (CH), 129.2 (CH), 128.2 (CH), 125.2 (CH), 124.0 (C), 120.5 (CH), 120.1 (C), 110.6 (CH), 55.5 (CH_2), 48.5 (CH_2). **HRMS** (ESI-TOF) m/z: $[M+H]^+$ Calcd for $C_{15}H_{14}N_3O_2$ 267.1081; Found 267.1084.

2j: 3-phenylbenzo[*d*][1,2,3]triazin-4(3*H*)-one

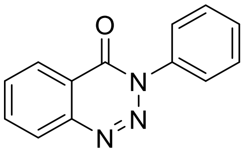

Following the general flow procedure, using 45 mg (0.152 mmol) of starting material **1j** in 4 mL (0.038 M) of MeCN/H_2O (2:1). 28 mg of the title compound (82% yield, 0.125 mmol) were obtained as a white solid after chromatography. Eluent used in the isolation: DCM: AcOEt (9:1). **^{1}H NMR (500 MHz, $CDCl_3$)** δ 8.46 (ddd, *J* = 7.9, 1.4, 0.6 Hz, 1H), 8.24 (ddd, *J* = 8.2, 1.2, 0.6 Hz, 1H), 8.00 (ddd, *J* = 8.2, 7.3, 1.5 Hz, 1H), 7.86 (ddd, *J* = 7.9, 7.3, 1.2 Hz, 1H), 7.68 – 7.64 (m, 2H), 7.60 – 7.54 (m, 2H), 7.52 – 7.47 (m, 1H). The reported data is in accordance with literature.[3]

2k: *rac*-methyl 2-(4-oxobenzo[*d*][1,2,3]triazin-3(4*H*)-yl)-3-phenylpropanoate

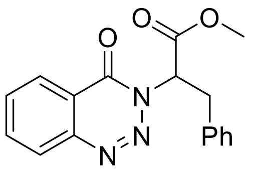

Following the general flow procedure, using 50 mg (0.131 mmol) of starting material **1k** in 2.6 mL (0.05 M) of MeOH/DCM (1:3). 35.5 mg of the title compound (88% yield, 0.115 mmol) were obtained as a white solid after chromatography. Eluent used in the isolation: Pentane: AcOEt (6:1). **M. p.**: 96 – 98 ºC. **^{1}H NMR (400 MHz, $CDCl_3$)** δ 8.32 (dd, J = 7.9, 1.4 Hz, 1H, ArH), 8.16 – 8.08 (m, 1H, ArH), 7.91 (ddd, J = 8.3, 7.2, 1.5 Hz, 1H, ArH), 7.76 (ddd, J = 8.2, 7.2, 1.2 Hz, 1H, ArH), 7.59 – 7.52 (m, 2H, ArH), 7.38 – 7.24 (m, 3H, ArH), 6.70 (dd, J = 10.0, 5.6 Hz, 1H, CHN), 3.82 (dd, J = 16.9, 10.1 Hz, 1H, C*H*H), 3.63 (s, 3H, CH_3), 3.31 (dd, J = 16.9, 5.7 Hz, 1H, C*H*H). **$^{13}C\{^1H\}$ NMR (101 MHz, $CDCl_3$)** δ 170.6 (CO), 155.0 (CO), 143.8 (C), 138.3 (C), 134.8 (CH), 132.3 (CH), 128.8 (2xCH), 128.5 (CH), 128.2 (CH), 127.6 (2xCH), 125.4 (CH), 119.7 (C), 56.9 (CH), 51.9 (CH_3), 38.4 (CH_2). **HRMS** (ESI-TOF) m/z: $[M+H]^+$ Calcd for $C_{17}H_{16}N_3O_3$ 310.1186; Found 310.1189.

2l: methyl 3-cyclopentyl-4-oxo-3,4-dihydrobenzo[*d*][1,2,3]triazine-7-carboxylate

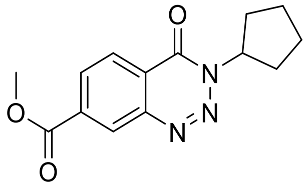

Following the general flow procedure, using 50 mg (0.203 mmol) of starting material **1l** in 4 mL (0.05 M) of MeOH/DCM (3.1). 46.5 mg of the title compound (84% yield, 0.170 mmol) were obtained as a silver-white solid after chromatography. Eluent used in the isolation: Pentane: AcOEt (4:1). **M. p.**: 116 – 118 ºC. **^{1}H NMR (400 MHz, $CDCl_3$)** δ 8.77 (dd, J = 1.5, 0.7 Hz, 1H, ArH), 8.46 – 8.28 (m, 2H, ArH), 5.52 (tt, J = 8.1, 6.8 Hz, 1H, CH), 4.02 (s, 3H, CH_3), 2.30 – 2.07 (m, 4H, $2xCH_2$), 1.99 (ddt, J = 8.8, 7.4, 2.5 Hz, 2H, CH_2), 1.86 – 1.69 (m, 2H, CH_2). **$^{13}C\{^1H\}$ NMR (101 MHz, $CDCl_3$)** δ 165.2 (CO), 154.8 (CO), 143.7 (C), 135.9 (C), 132.0 (CH), 129.8 (CH), 125.7 (CH), 122.1 (CH), 58.8 (CH), 52.9 (CH_3), 32.2 ($2xCH_2$), 24.9 ($2xCH_2$). **HRMS** (TOF-ESI+) m/z: $[M+H]^+$ Calcd for $C_{14}H_{16}N_3O_3$ 274.1186; Found 274.1190.

2m: 3-isobutyl-[1,3]dioxolo[4',5':4,5]benzo[1,2-*d*][1,2,3]triazin-4(3*H*)-one

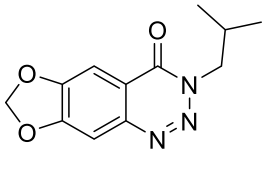

Following the general flow procedure, using 50 mg (0.156 mmol) of starting material **1m** in 3.1 mL (0.05 M) of MeOH/DCM (3.1). 37 mg of the title compound (96% yield, 0.150 mmol) as a pale-yellow solid. The product was purified by solvent evaporation followed by redissolving the solid in

DCM (5 mL) and the organic layer was washed twice with brine (5 mL) and dried with sodium sulphate. **M. p.:** 124 – 126 ºC. **^{1}H NMR (400 MHz, $CDCl_3$)** δ 7.60 (s, 1H, ArH), 7.41 (s, 1H, ArH), 6.20 (s, 2H, CH_2), 4.25 (d, *J* = 7.3 Hz, 2H, CH_2N), 2.41 – 2.28 (m, 1H, CH), 0.98 (d, *J* = 6.7 Hz, 6H, 2xCH_3). **$^{13}C\{^1H\}$ NMR (101 MHz, $CDCl_3$)** δ 155.4 (CO), 153.4 (C), 151.5 (C), 142.3 (C), 116.5 (C), 105.9 (CH), 102.9 (CH_2), 101.9 (CH), 56.6 (CH_2), 28.1 (CH), 19.9 (2xCH_3). **HRMS** (ESI-TOF) m/z: $[M+H]^+$ Calcd for $C_{12}H_{14}N_3O_3$ 248.1030; Found 248.1033. **IR** (neat) ν/cm^{-1}: 2912 (w), 1662 (s), 1616 (w), 1472 (m) 1418 (w), 1290 (m), 1274 (w), 1132 (w), 1034 (s), 937 (w), 864 (m), 813 (w), 791 (w), 755 (w), 677 (w), 598 (w), 551 (w), 502 (w), 461 (w), 450 (w).

2n: 3-benzyl-6-fluorobenzo[*d*][1,2,3]triazin-4(3*H*)-one

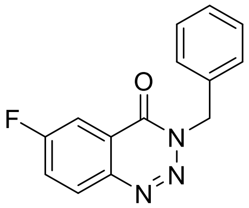

Following the general flow procedure, using 45 mg (0.116 mmol) of starting material **1n** in 2.3 mL (0.05 M) of MeOH/DCM (1:3). 27.5 mg of the title compound (93% yield, 0.108 mmol) were obtained as a white solid after chromatography. Eluent used in the isolation: Pentane: AcOEt (6:1). White crystals suitable for X-ray were obtained by slow evaporation of the white solid in DCM with drops of MeOH. **M. p.:** 105 – 106 ºC. **^{1}H NMR (400 MHz, $CDCl_3$)** δ 8.19 (dd, *J* = 9.0, 4.8 Hz, 1H, ArH), 7.95 (dd, *J* = 7.9, 2.8 Hz, 1H, ArH), 7.62 (ddd, *J* = 8.9, 8.0, 2.8 Hz, 1H, ArH), 7.54 – 7.47 (m, 2H, ArH), 7.38 – 7.28 (m, 3H, ArH), 5.62 (s, 2H, CH_2). **$^{13}C\{^1H\}$ NMR (101 MHz, $CDCl_3$)** δ 164.1 (d, *J* = 257.4 Hz, C), 154.7 (d, *J* = 3.1 Hz, CO), 141.4 (d, *J* = 2.1 Hz, CO), 135.5 (C), 131.5 (d, *J* = 9.1 Hz, CH), 128.9 (2xCH), 128.8 (2xCH), 128.3 (CH), 123.6 (d, *J* = 24.5 Hz. CH), 122.3 (d, *J* = 9.6 Hz, CO), 110.5 (d, *J* = 24.1 Hz), 53.4 (CH_2). **^{19}F NMR (376 MHz, $CDCl_3$)** δ -102.34 (td, *J* = 8.0, 4.8 Hz). **HRMS** (ESI-TOF) m/z: $[M+H]^+$ Calcd for $C_{10}H_{11}FN_3O$ 256.0881, Found 256.0879.

2o: methyl 3-(4-isopropylphenyl)-4-oxo-3,4-dihydrobenzo[*d*][1,2,3]triazine-7-carboxylate

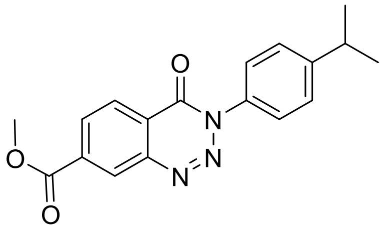

Following the general flow procedure, using 40 mg (0.101 mmol) of starting material **1o** in 2 mL (0.05 M) of MeOH/DCM (1:3). 17.5 mg of the title compound (54% yield, 0.055 mmol) were obtained as a white solid after chromatography. Eluent used in the isolation: Pentane: AcOEt (6:1). **M. p.:** 137 – 139 ºC. **^{1}H NMR (400 MHz, $CDCl_3$)** δ 8.87 (dd, *J* = 1.6, 0.6 Hz, 1H, ArH), 8.51 (dd, *J* = 8.3, 0.6 Hz, 1H, ArH), 8.44 (dd, *J* = 8.3, 1.6 Hz, 1H, ArH), 7.60 – 7.53 (m, 2H, ArH), 7.45 – 7.39 (m, 2H, ArH), 4.05 (s, 3H, CH_3), 3.02 (p, *J* = 6.9 Hz, 1H, CH), 1.31 (d, *J* = 6.9 Hz, 6H,

2xCH_3). **$^{13}C\{^1H\}$ NMR (101 MHz, $CDCl_3$)** δ 165.1 (CO), 154.7 (CO), 150.1 (C), 143.5 (C), 136.3 (C), 136.2 (C), 132.6 (CH), 130.2 (CH), 127.2 (2xCH), 126.2 (CH), 125.8 (2xCH), 123.2 (C), 53.0 (CH_3), 34.0 (CH), 23.9 (2xCH_3). **HRMS** (ESI-TOF) m/z: $[M+H]^+$ Calcd for $C_{18}H_{18}N_3O_3$ 324.1343; Found 324.1343.

2p: 3-(4-fluorophenyl)-[1,3]dioxolo[4',5':4,5]benzo[1,2-*d*][1,2,3]triazin-4(3*H*)-one

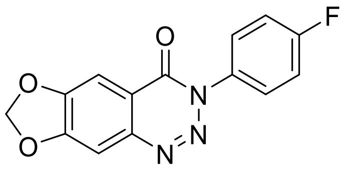

Following the general flow procedure, using 37 mg (0.104 mmol) of starting material **1p** in 2 mL (0.05 M) of MeOH/DCM (1:3). 19 mg of the title compound (76% yield, 0.079 mmol) were obtained as a pale-yellow solid after chromatography. Eluent used in the isolation: Cyclohexane: AcOEt (6:1). Note: the product coelutes with an inseparable unknown small impurity (96:4 according to F-NMR). **M. p.**: decop. **^1H NMR (500 MHz, $CDCl_3$)** δ 7.70 (s, 1H, ArH), 7.65 – 7.60 (m, 2H, ArH), 7.51 (s, 1H, ArH), 7.25 – 7.20 (m, 2H, 2xCH), 6.25 (s, 2H, CH_2). **^{19}F NMR (376 MHz, $CDCl_3$)** δ -112.09 (tt, *J* = 8.2, 4.8 Hz). **$^{13}C\{^1H\}$ NMR (126 MHz, $CDCl_3$)** δ 162.5 (d, *J* = 248.9 Hz, C), 155.0 (CO), 153.8 (C), 152.1 (C), 141.8 (C), 134.8 (d, *J* = 3.1 Hz, C), 127.9 (d, *J* = 8.8 Hz, 2xCH), 117.1 (C), 116.0 (d, *J* = 23.0 H, 2xCH), 106.5 (CH), 103.2 (CH_2), 102.5 (CH). **HRMS** (ESI-TOF) m/z: $[M+H]^+$ Calcd for $C_{14}H_9FN_3O_3$ 286.0622; Found 286.0624.

Characterization data of other compounds (3 and 4)

3: *N*-ethylbenzamide

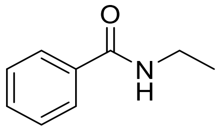

18.5 mg (0.124 mmol, 86% yield) of title compound were obtained as a colorless oil when using **1s** (0.145 mmol, 40 mg) with the general flow protocol. Chromatography in Pentane:Et_2O (2:1) **^{11}H NMR (400 MHz, $CDCl_3$)** δ 7.78 – 7.73 (m, 2H, ArH), 7.52 – 7.46 (m, 1H, ArH), 7.45 – 7.40 (m, 2H, ArH), 6.09 (bs, 1H, NH), 3.59 – 3.41 (m, 2H, CH_2), 1.26 (t, *J* = 7.2 Hz, 3H, CH3).The reported data is in accordance with literature.[6]

4: benzo[*c*]cinnoline

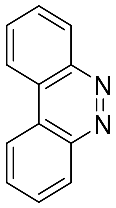

The title compound was observed as the major compound (NMR yield 44% yield) when using **1t** (40 mg, 0.149 mmol) in 4 mL of DCM/MeOH (0.037 M) with the general flow protocol. **^{1}H NMR (400 MHz, $CDCl_3$)** δ 8.81 – 8.73 (m, 2H, ArH), 8.65 – 8.58 (m, 2H, ArH), 7.98 – 7.89 (m, 4H, ArH). The reported data is in accordance with literature.[7]

X-Ray data

- Summary of X-ray data for compounds 1b, 1c and 2n:

For each sample, a suitable single crystal was removed from the growing vessel and manipulated in a perfluoropolyalkylether oil matrix (F06206K, ABCR company) on a standard glass microscope slide. The selected crystal was mounted in the end of a cactus fibre afixed to a copper pin magnetically attached to a standard goniometer head which was placed in the Euler cradle and flashed cooled with a cold blanket of N2 gas using an Oxford Cryosystems cooling device. The mounted crystal was kept under a (<110 K) gaseous flow of N_2 during the entire collection process. A complete diffraction data set was collected on a 4-circle dual source Rigaku SuperNova A instrument equipped with a 140 mm Atlas-model CCD detector using mono-chromated Cu-Kα (λ=1.54184 Å, 50.0 kV, 0.8 mA) radiation generated by a microfocus source. For data collection, optimized ω-scans were employed and raw data treatment including cell indexing (using reflections measured from the entire data set), space group determination, raw data reduction and gaussian-based adsorption corrections[8] were performed with the CrysAlisPro application (version 42).[9] A small number of reflections were filtered out when the associated Rint value was particularly large. In the case of **2n**, the crystal presented with non-monohedral rotational twinning where two-unit cells (using the twin law -1 0 0 0 1 0 0.628 0 0.03) were identified. Initially, a hklf4 file was generated from a set of one unit cell parameters and used to generate a preliminary solution, then refinement was completed with a merged hlkf5 file.

The structures were solved by intrinsic phasing using the SHELXT program.[10] Refinement was performed by full matrix least squares minimization on F^2 for all data using SHELX-LS.[11] Hydrogen atoms were added at calculated positions and refined using a riding model based on the connecting atom. Where electron density was sufficient, the hydrogen atoms were located manually from electron density map and the positions were allowed to ride to the attached atom. The associated isotropic thermal displacement parameters for the hydrogen atoms were fixed to 1.2 times (1.5 times for the methyl groups) to that of the attached atom. For hydrogen atoms that were engaged with hydrogen bonding with heteroatoms, these particular hydrogen were located on the difference map and allowed to refine. Anisotropic thermal displacement parameters were used for all non-hydrogen atoms.

None of the structure reported displayed positional disorder. Structure solution, refinement and structural analysis were performed using the program Olex2 (version 1.5-ac).

- Table 1: Key Parameters of Reported Structures

Identification code	(**1b**) mb1926	(**2n**) mb1936	(**1c**) mb1936
CCDC Code	2326279	2326280	2326278
Empirical formula	$C_{12}H_{14}N_4O_4$	$C_{14}H_{10}FN_3O$	$C_{10}H_{11}FN_4O_2$
Formula weight	278.27	255.25	238.23
Temperature/K	140(1)	106.7(2)	106.8(2)
Crystal system	triclinic	monoclinic	orthorhombic
Space group	P_{-1}	$P2_1/n$	$Pna2_1$
a/Å	3.95230(10)	7.96380(10)	28.3057(4)
b/Å	12.4023(3)	5.62640(10)	12.9854(2)
c/Å	13.3950(4)	25.9228(4)	5.92720(10)
α/°	81.919(2)	90	90
β/°	83.574(2)	95.4250(10)	90
γ/°	88.433(2)	90	90
Volume/Å^3	645.94(3)	1156.33(3)	2178.61(6)
Z	2	4	8
ρcalcg/cm^3	1.431	1.466	1.453
μ/mm^{-1}	0.928	0.895	0.993
F(000)	292.0	528.0	992.0
Crystal size/mm^3	0.62 × 0.12 × 0.1	0.39 × 0.38 × 0.08	0.44 × 0.13 × 0.07
Radiation	Cu Kα (λ=1.54184)	Cu Kα (λ=1.54184)	Cu Kα (λ=1.54184)
2Θ range for data collection/°	6.706 to 152.264	6.85 to 148.99	6.246 to 148.852
Index ranges	-4 ≤ h ≤ 4, -15 ≤ k ≤ 15, -16 ≤ l ≤ 16	-9 ≤ h ≤ 9, 0 ≤ k ≤ 7, 0 ≤ l ≤ 32	-33 ≤ h ≤ 35, -15 ≤ k ≤ 16, -7 ≤ l ≤ 7
Reflections collected	15104	2343	26634
Independent reflections	2658 [Rint = 0.0292, Rsigma = 0.0133]	2343 [Rsigma = 0.0159]	4347 [Rint = 0.0599, Rsigma = 0.0280]
Data/restraints/parameters	2658/0/192	2343/0/174	4347/1/327
Goodness-of-fit on F^2	1.037	1.084	1.055
Final R indexes [I>=2σ (I)]	R_1 = 0.0334, wR_2 = 0.0921	R1 = 0.0442, wR2 = 0.1191	R_1 = 0.0370 wR_2 = 0.1021
Final R indexes [all data]	R_1 = 0.0355, wR_2 = 0.0947	R1 = 0.0451, wR_2 = 0.1197	R_1 = 0.0391, wR_2 = 0.1049
Largest diff. peak/hole / e Å^{-3}	0.25/-0.24	0.25/-0.23	0.16/-0.24
Flack parameter	n/a	n/a	0.05(11)

- Image of Crystal Structure for **1b** (CCDC 2326279)

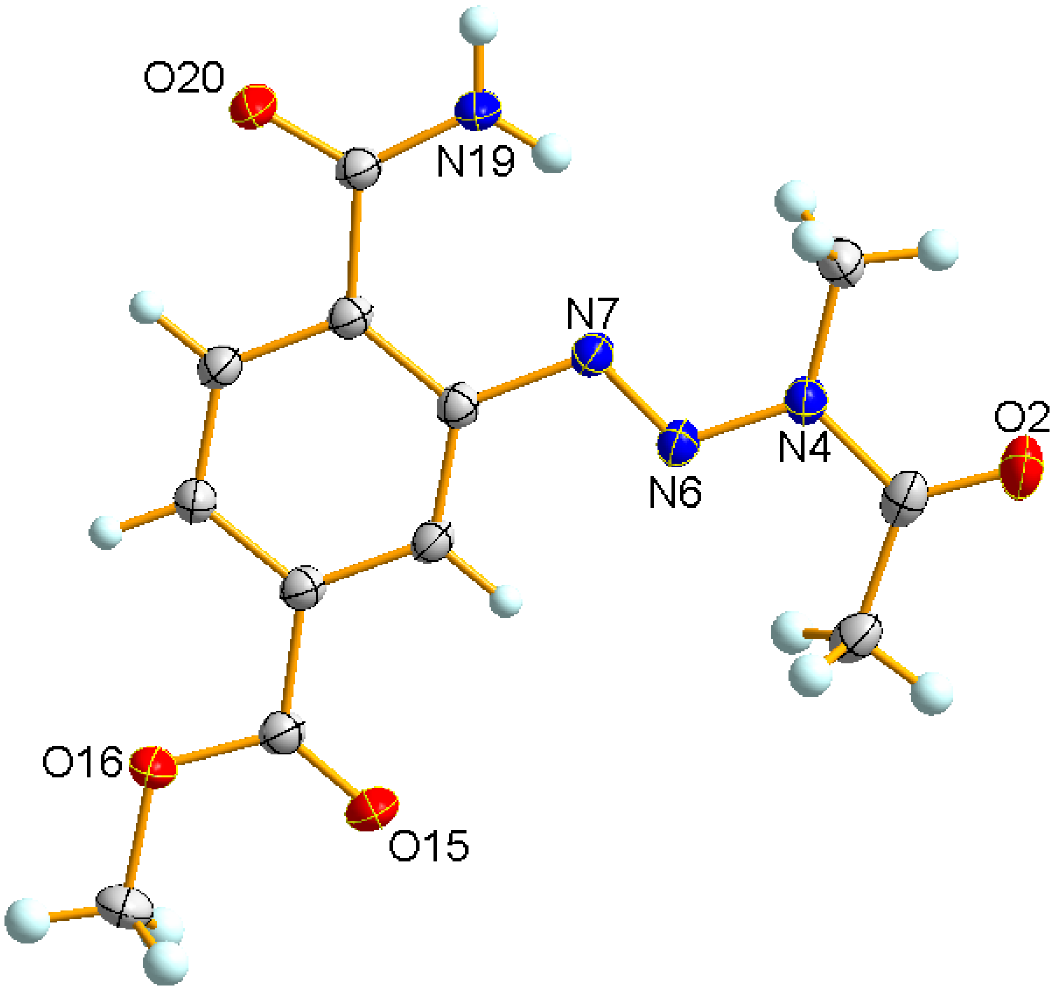

ORTEP diagram of **1b** drawn with 50% probability ellipsoids.

- Image of Crystal Structure for **1c** (CCDC 2326278)

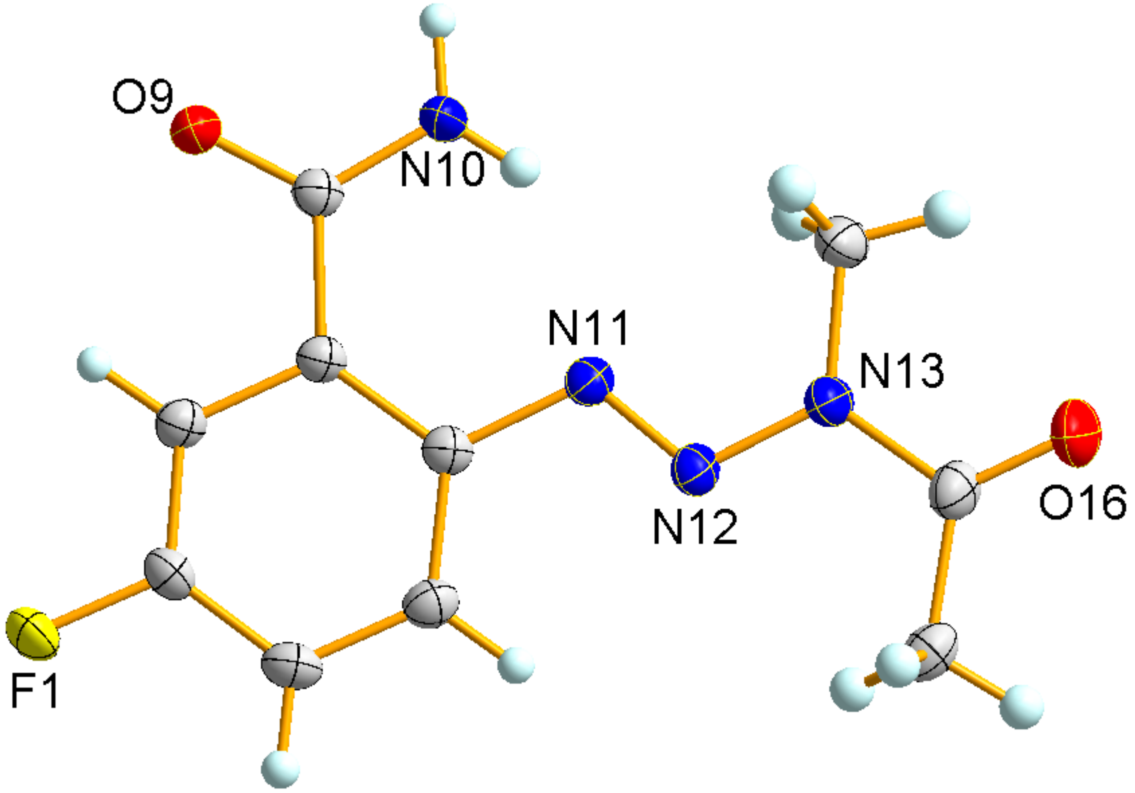

ORTEP diagram of **1c** drawn with 50% probability ellipsoids.

- Image of Crystal Structure for **2n** (CCDC 2326280)

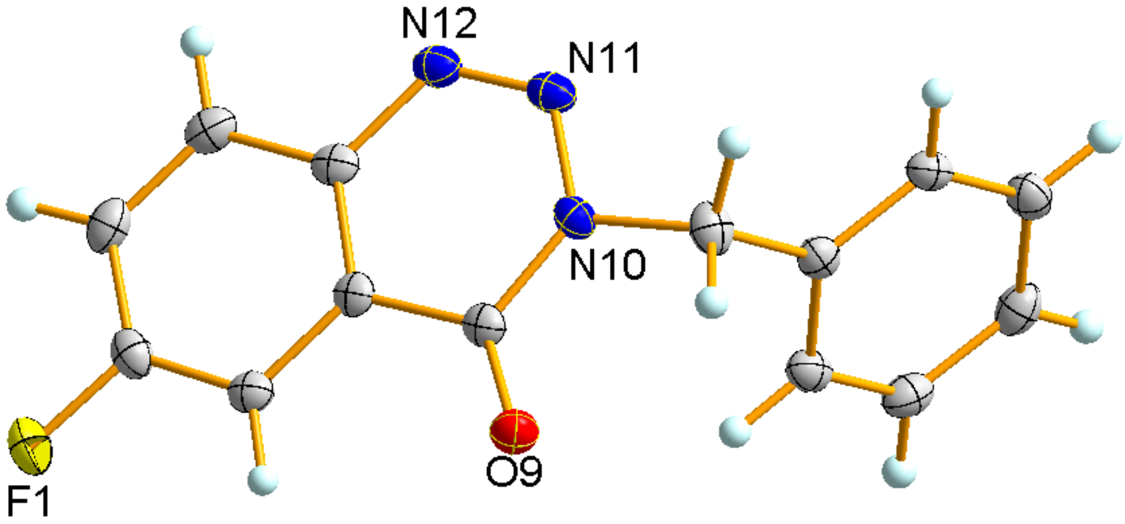

ORTEP diagram of **2n** drawn with 50% probability ellipsoids.

Pictures of the flow equipment

Flow set-up during 1 g long run

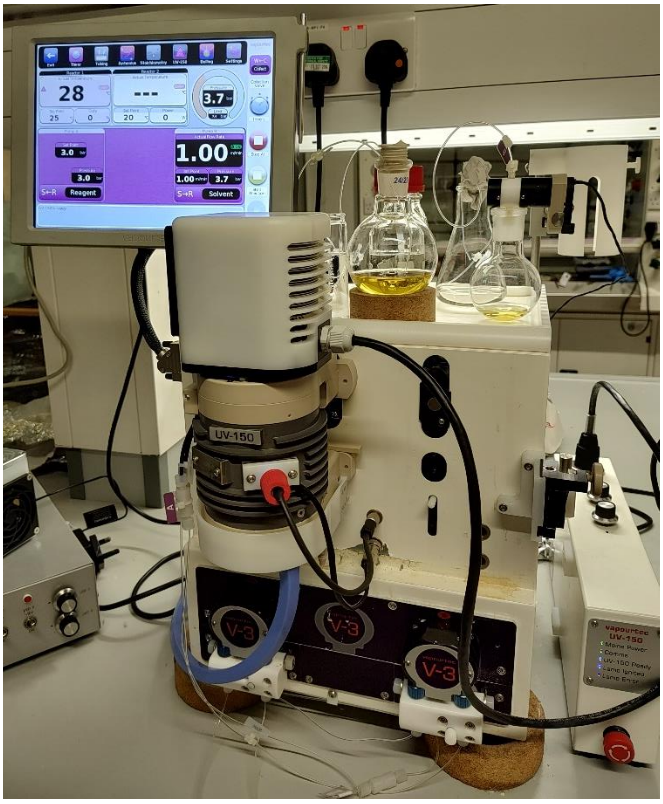

10 mL coil reactor:

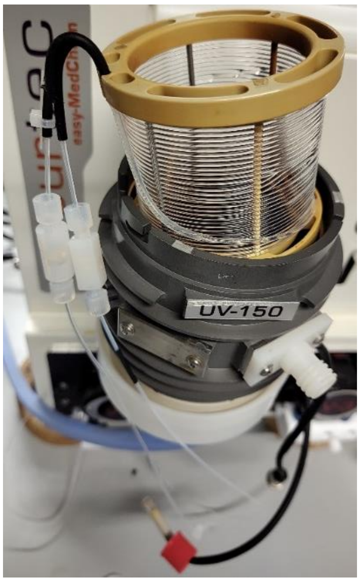

References

(1) García-Lacuna, J.; Baumann, M. Modular Photochemical Flow Synthesis of Structurally Diverse Benzyne and Triazine Precursors. *Adv. Synt. Catal.* **2023**, *365*, 2628–2635. (ref. 15 of main manuscript)

(2) Zhou, Y.; Wang, Y.; Lou, Y.; Song, Q. Oxidative Rearrangement of 3-Aminoindazoles for the Construction of 1,2,3-Benzotriazine-4(3H)-Ones at Ambient Temperature. *Org. Lett.* **2018**, *20*, 6494–6497. (ref. 13 of main manuscript)

(3) Cai, Y.-M.; Zhang, X.; An, C.; Yang, Y.-F.; Liu, W.; Gao, W.-X.; Huang, X.-B.; Zhou, Y.-B.; Liu, M.-C.; Wu, H.-Y. Catalyst-Free Oxidative N–N Coupling for the Synthesis of 1,2,3-Triazole Compounds with tBuONO. *Org. Chem. Front.* **2019**, *6*, 1481–1484. (ref. 10b of main manuscript)

(4) Madasamy, K.; Balakrishnan, M. H.; Korivi, R.; Mannathan, S. Trifluoroacetic Acid-Mediated Denitrogenative Ortho-Hydroxylation of 1,2,3-Benzotriazin-4(3H)-Ones: A Metal-Free Approach. *J. Org. Chem.* **2022**, *87*, 8752–8756. (ref. 5 of main manuscript)

(5) Dong, B.; Liu, Y.; Yang, P.; Sang, D.; Tian, J.; Li, L.; Long, S. Denitrogenative Cleavage of Benzotriazoles and Benzotriazinones, and Selective N-Desulfonylation of Benzotriazoles by Aluminum Halides. *Tetrahedron. Lett.* **2022**, *103*, 153965.

(6) Ghosh, S. C.; Ngiam, J. S. Y.; Chai, C. L. L.; Seayad, A. M.; Dang, T. T.; Chen, A. Iron-Catalyzed Efficient Synthesis of Amides from Aldehydes and Amine Hydrochloride Salts. *Adv. Synth. Catal.* **2012**, *354*, 1407–1412.

(7) Bjørsvik, H.-R.; González, R. R.; Liguori, L. Investigations of a Novel Process to the Framework of Benzo[c]Cinnoline. *J. Org. Chem.* **2004**, *69*, 7720–7727. (ref. 16 of main manuscript)

(8) Coppens, P.; Hamilton, W. C. Anisotropic extinction corrections in the Zachariasen approximation. *Acta Cryst.* **1970**, *A26*, 71–83.

(9) Sheldrick, G. M. SHELXT − Integrated Space-Group and Crystal-Structure Determination. *Acta Crystallogr.* **2015**, *A71*, 3–8.

(10) Sheldrick, G. M. Crystal Structure Refinement with SHELXL. Acta Crystallogr. **2015**, *C71*, 3–8.

(11) Dolomanov, O.V.; Bourhis, L.J.; Gildea, R.J.; Howard, J.A.K.; Puschmann, H., OLEX2: A complete structure solution, refinement and analysis program. *J. Appl. Cryst.*, **2009**, *42*, 339–341.

Copies of NMR data

NMR copies of starting materials (**1b**-**1t**)

1b: (*E*)-2-(3-acetyl-3-methyltriaz-1-en-1-yl)-5-fluorobenzamide

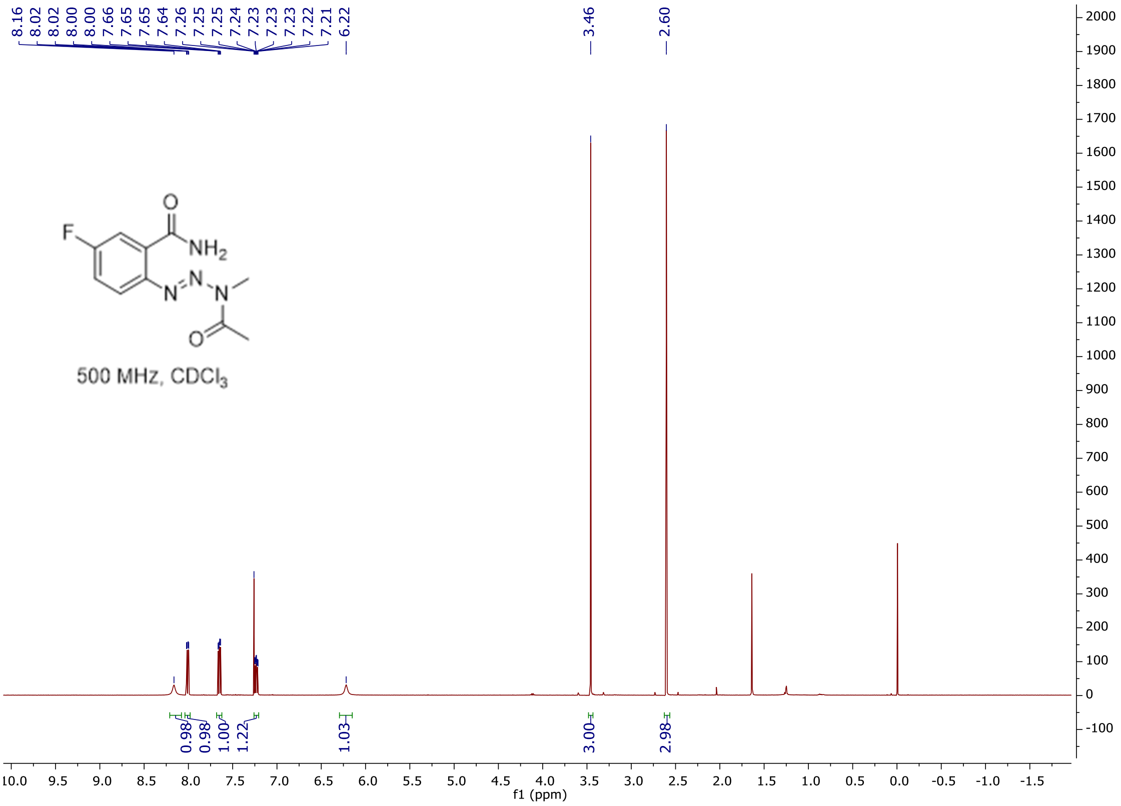

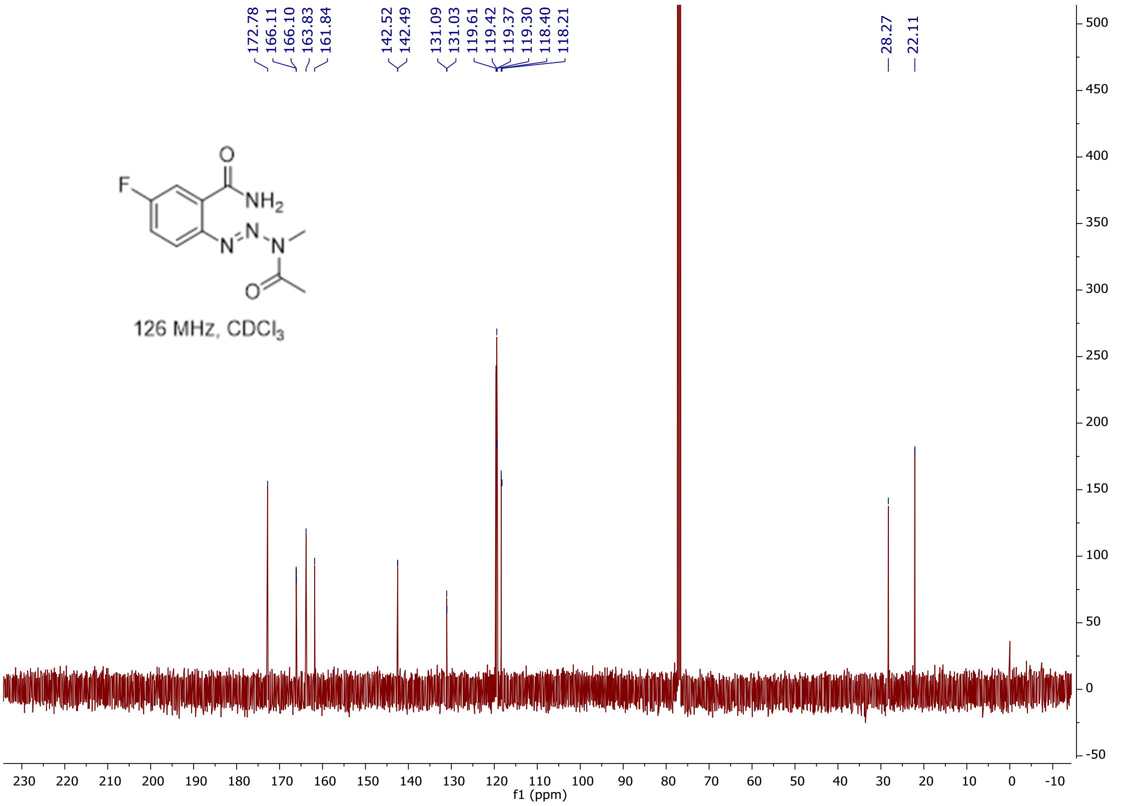

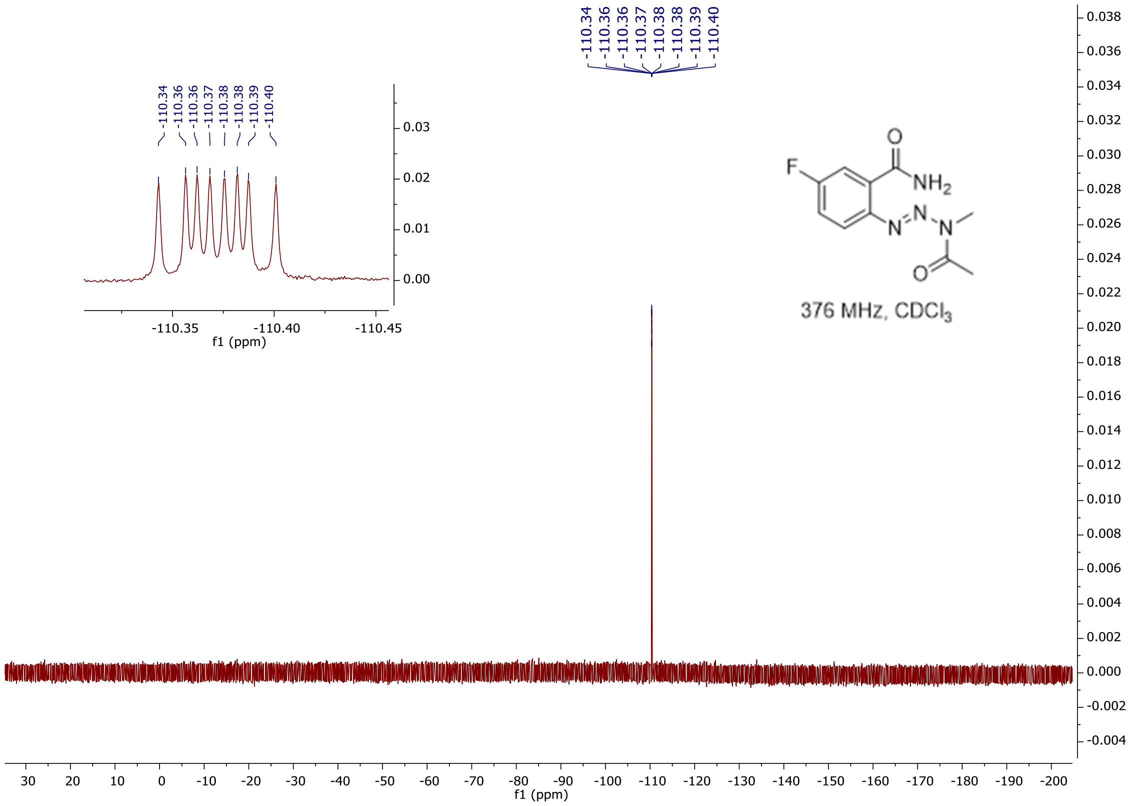
-110.34
-110.36
-110.36
-110.37
-110.38
-110.38
-110.39
-110.40
376 MHz, CDCl3
f1 (ppm)

1c: methyl (*E*)-3-(3-acetyl-3-methyltriaz-1-en-1-yl)-4-carbamoylbenzoate

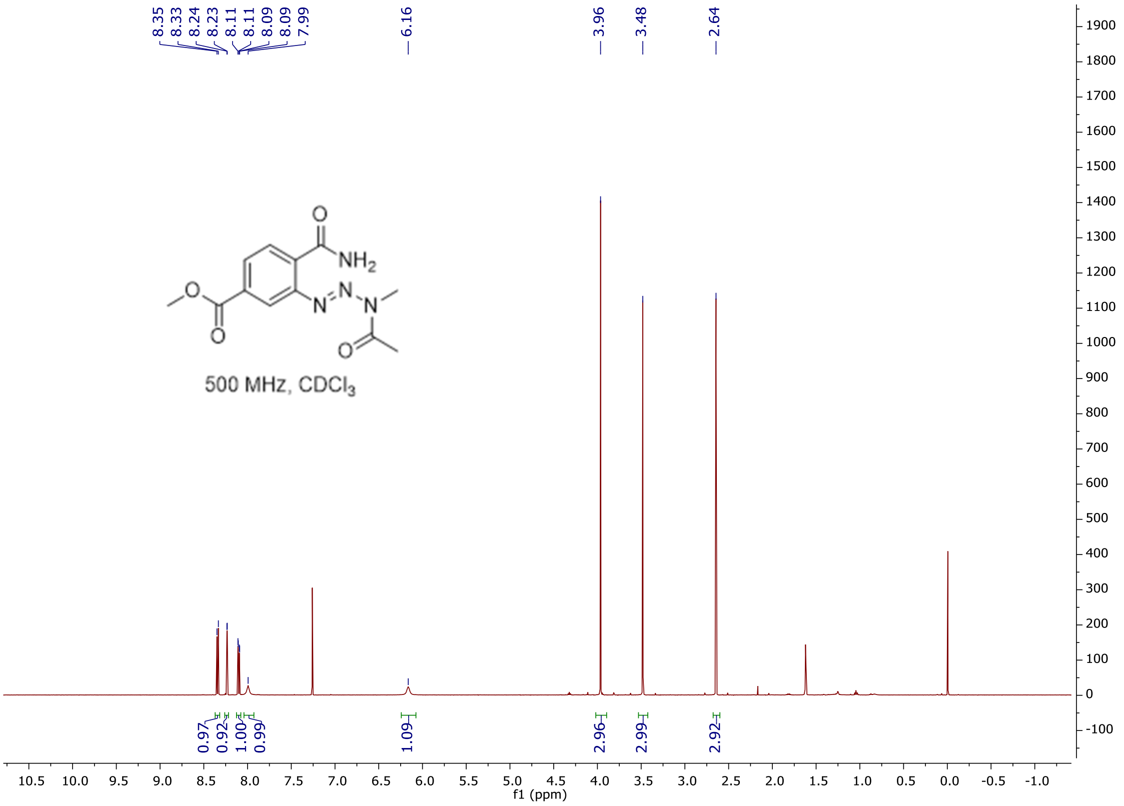

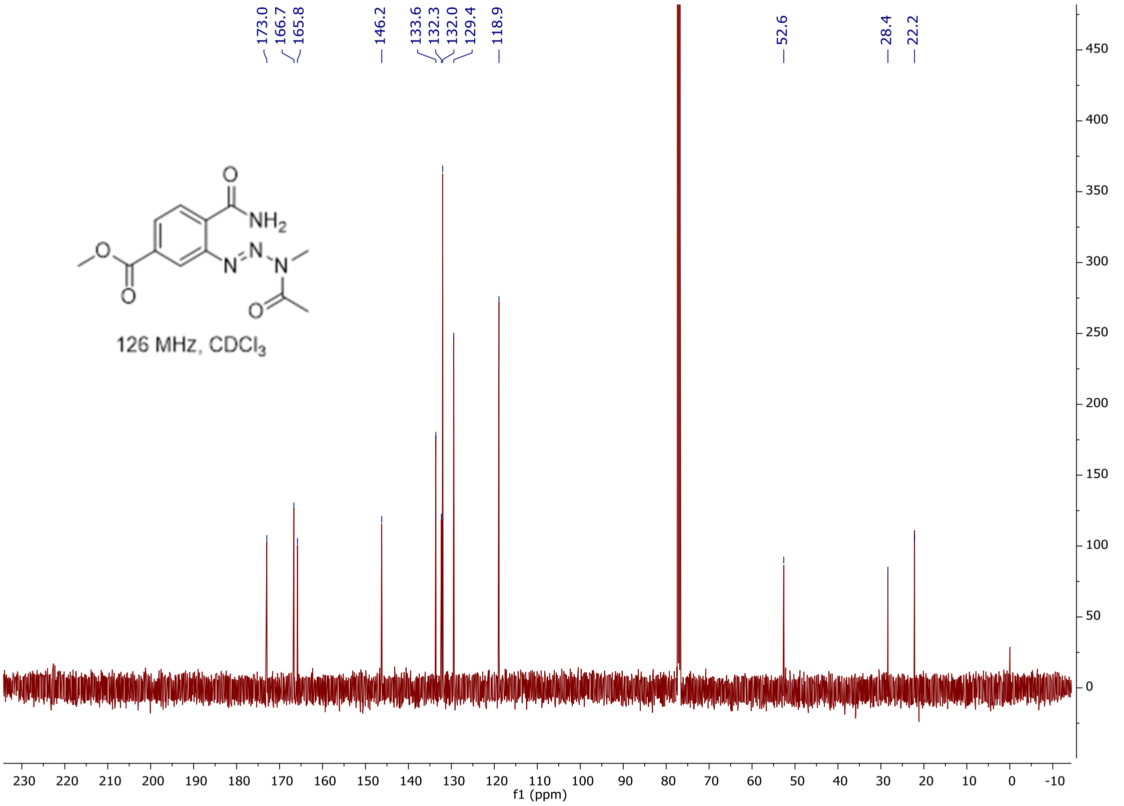

1d: (*E*)-2-(3-acetyl-3-methyltriaz-1-en-1-yl)-5-methoxybenzamide

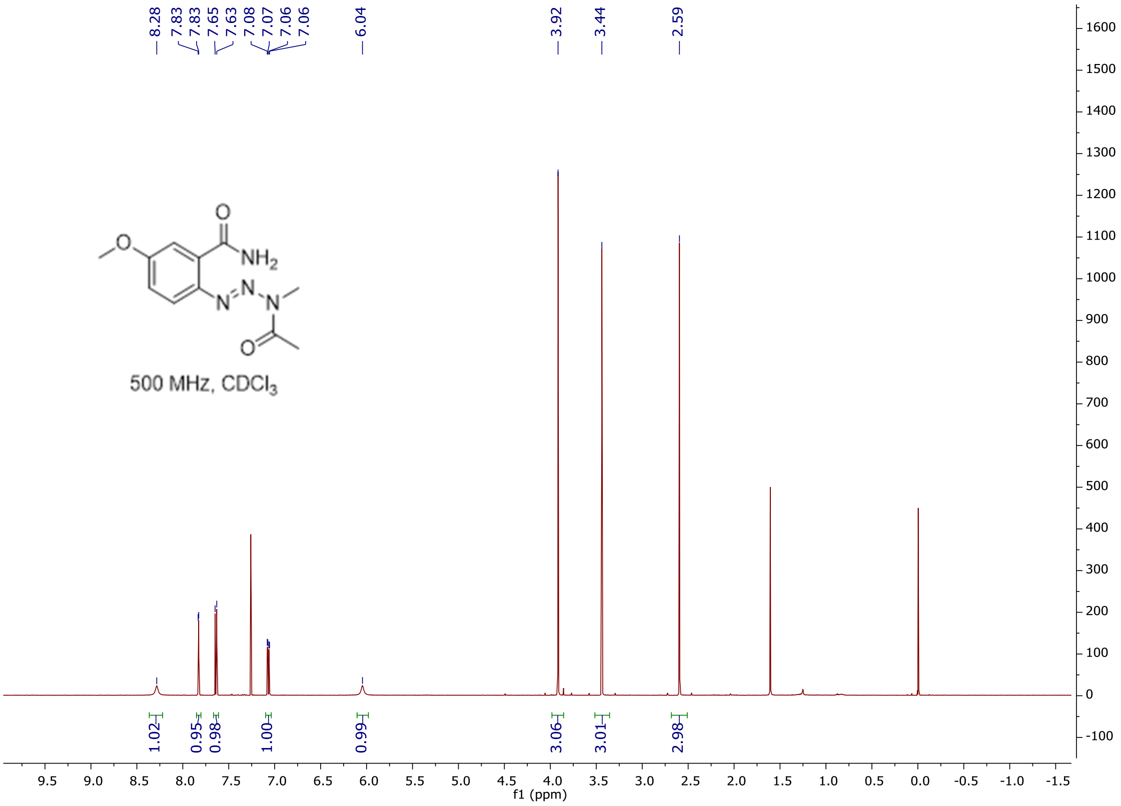

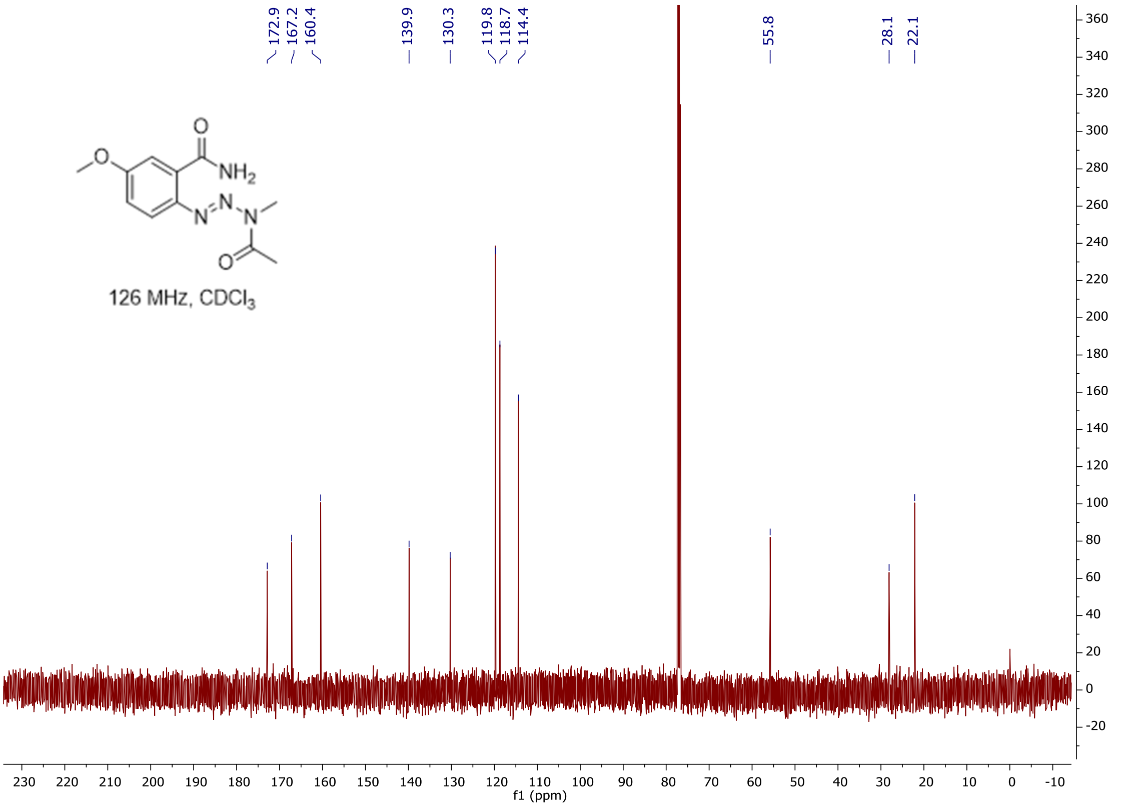

1e: (*E*)-6-(3-acetyl-3-methyltriaz-1-en-1-yl)benzo[*d*][1,3]dioxole-5-carboxamide

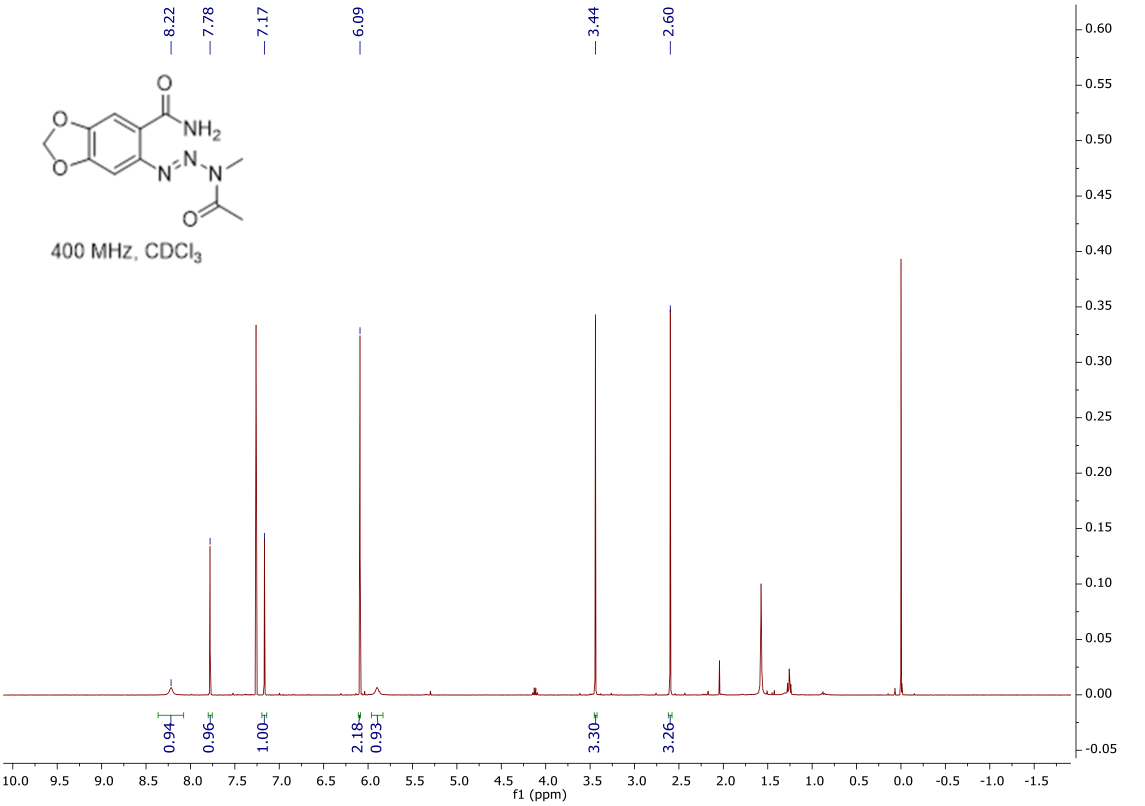

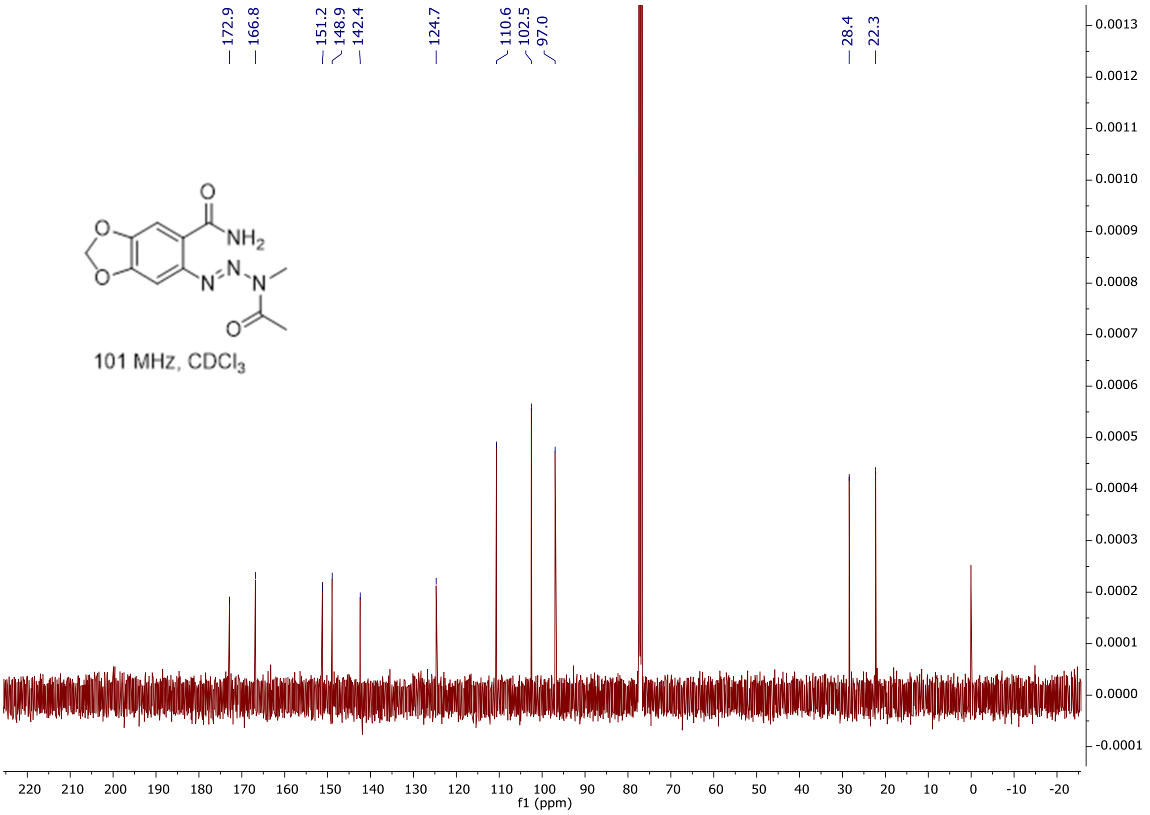

1h: (*E*)-2-(3-acetyl-3-methyltriaz-1-en-1-yl)-*N*-(furan-2-ylmethyl)benzamide

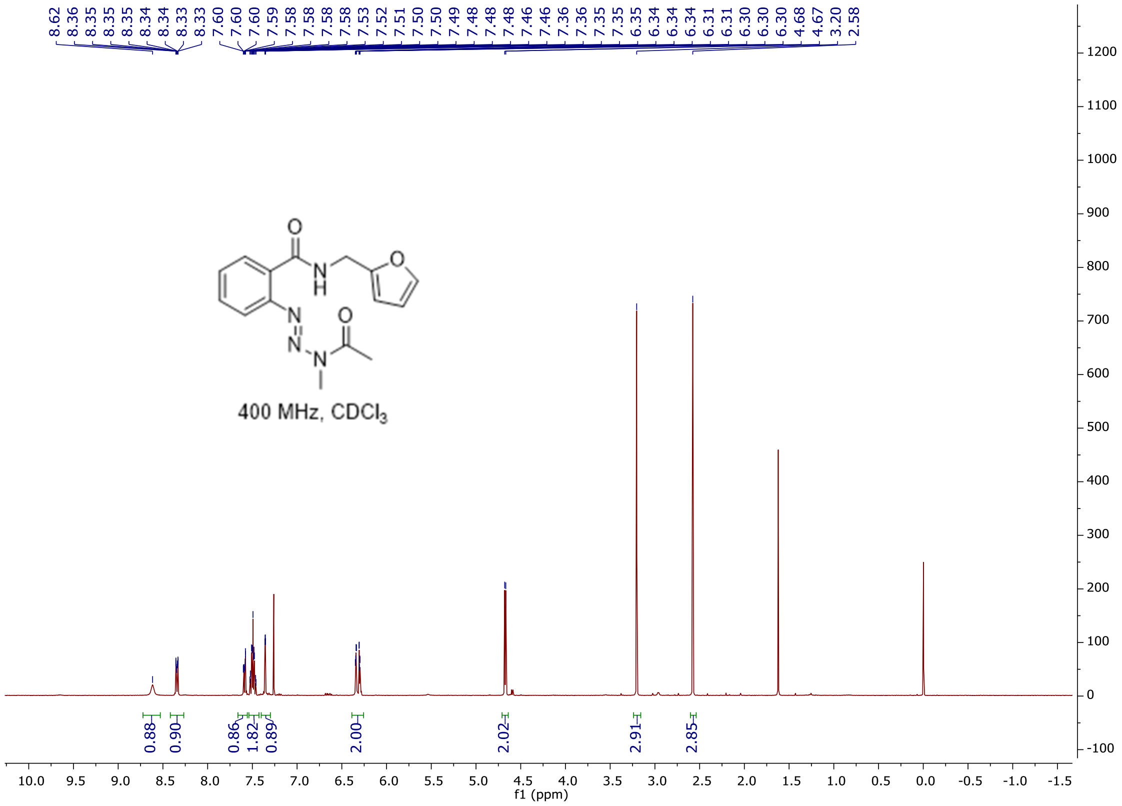

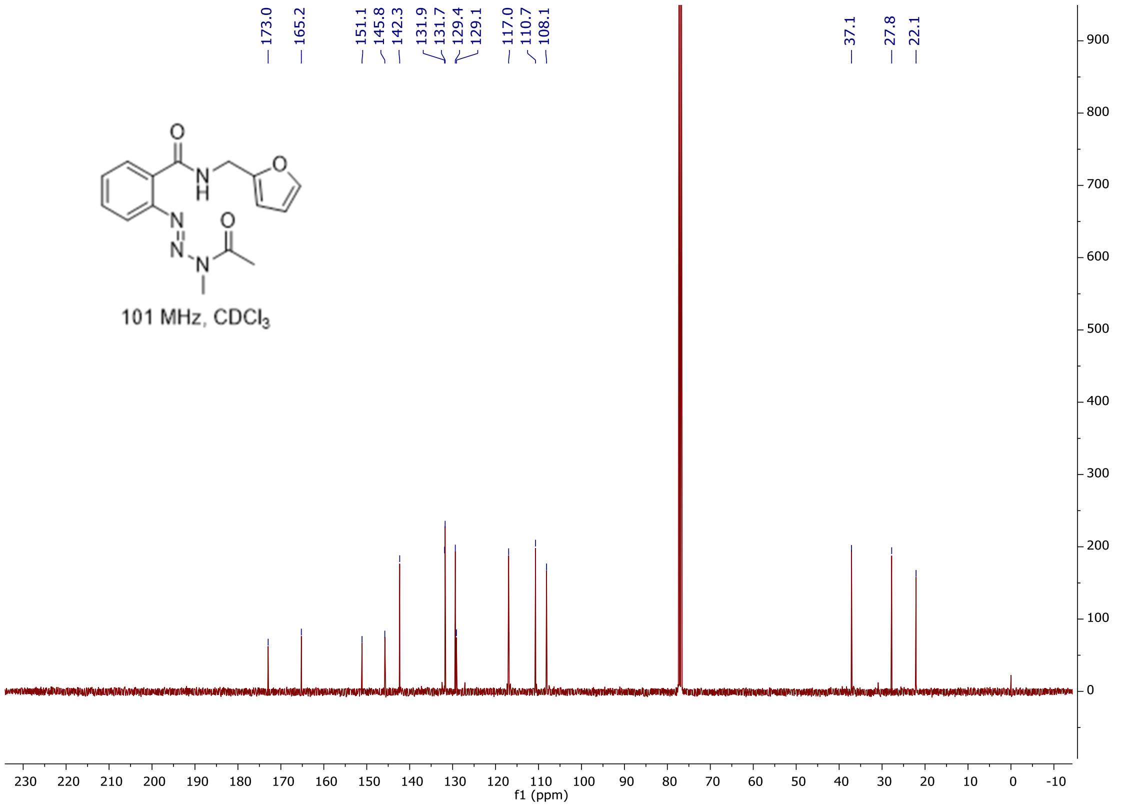

1i: (*E*)-2-(3-acetyl-3-methyltriaz-1-en-1-yl)-*N*-(2-methoxybenzyl)benzamide

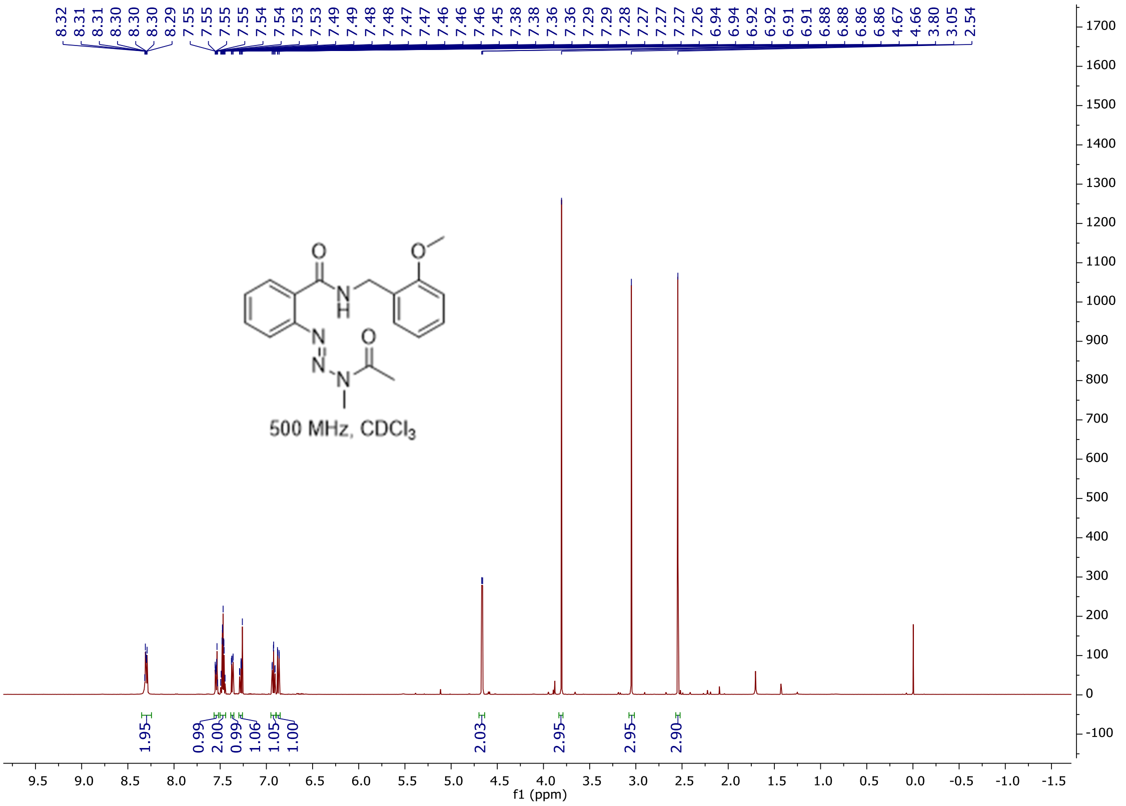

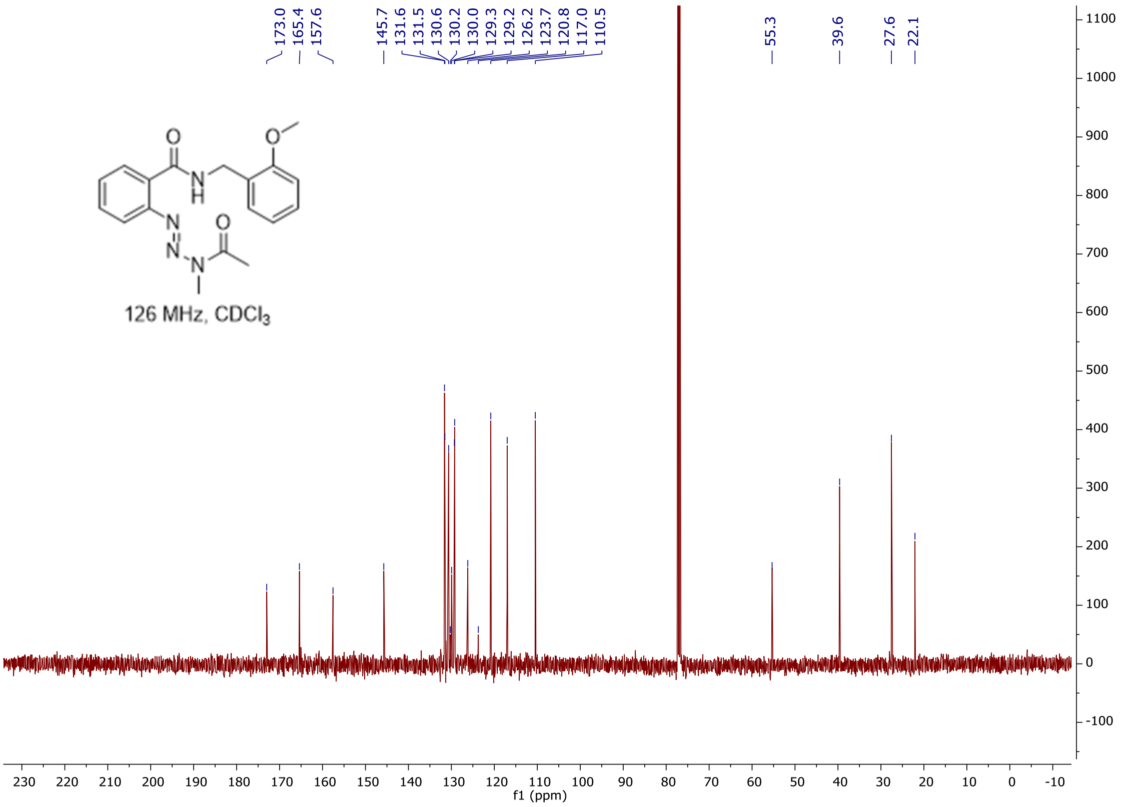

1j: (*E*)-2-(3-acetyl-3-methyltriaz-1-en-1-yl)-*N*-phenylbenzamide

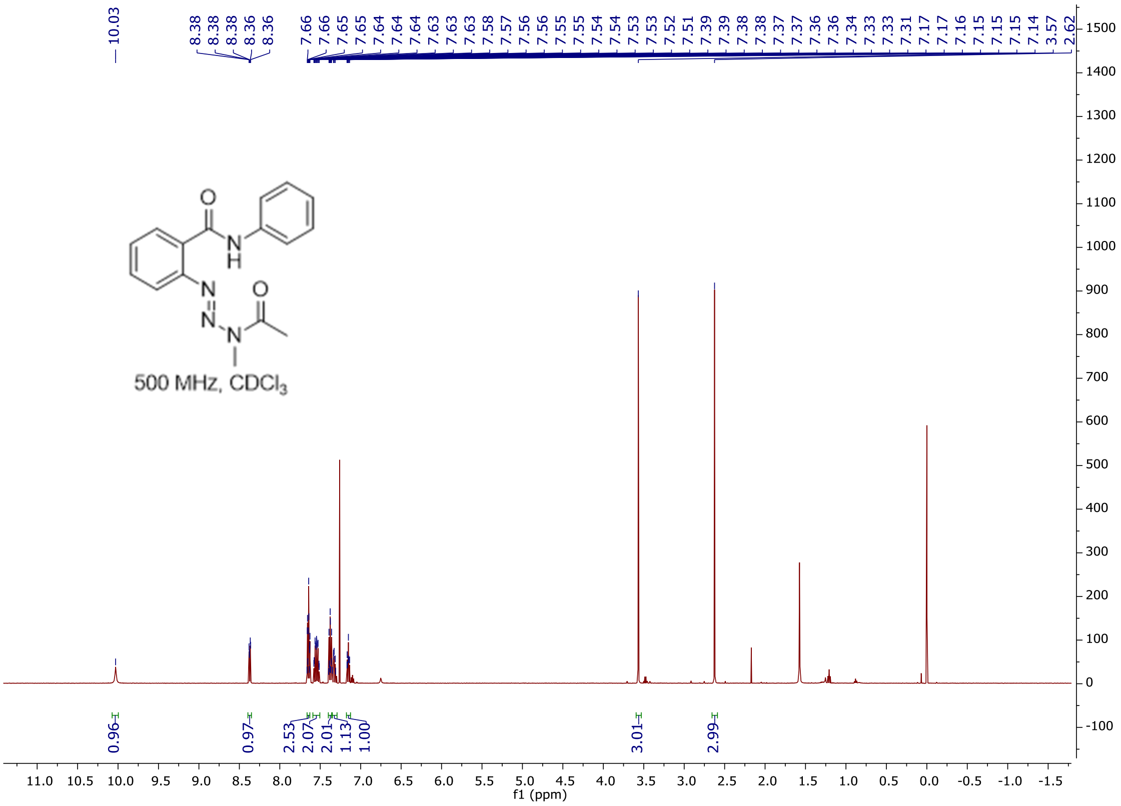

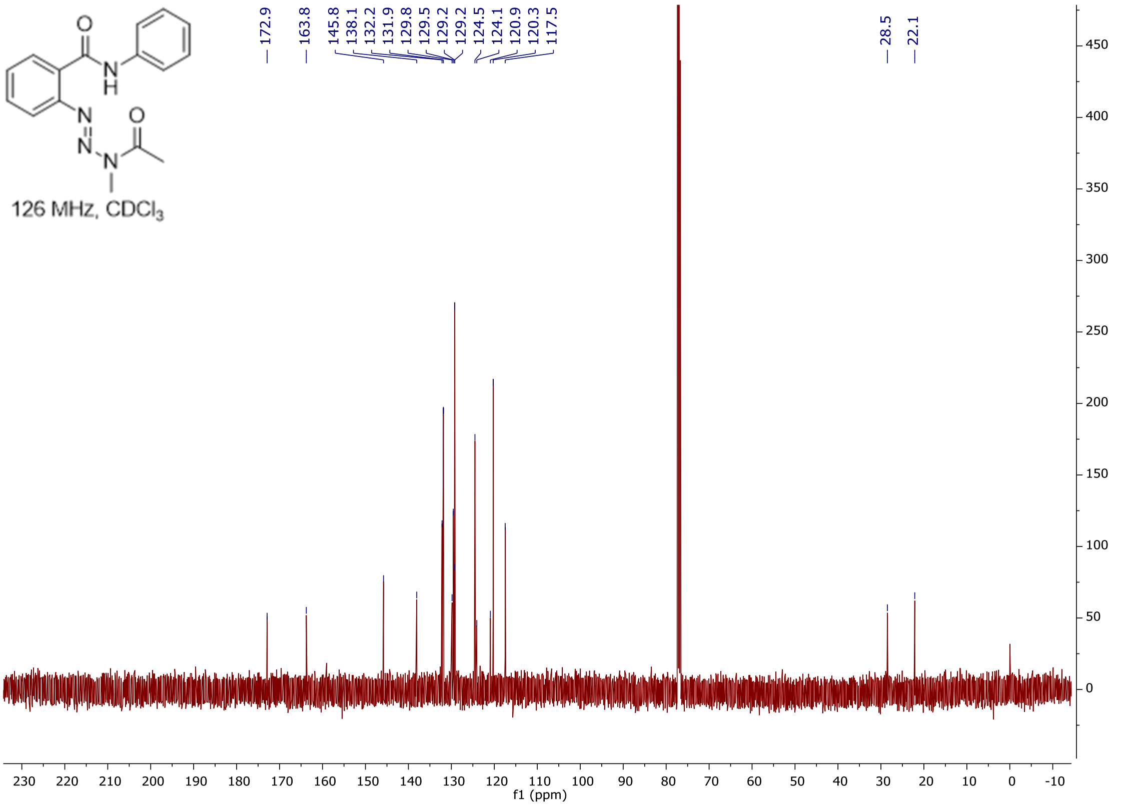

1k: *rac*-methyl (*E*)-(2-(3-acetyl-3-methyltriaz-1-en-1-yl)benzoyl)phenylalaninate

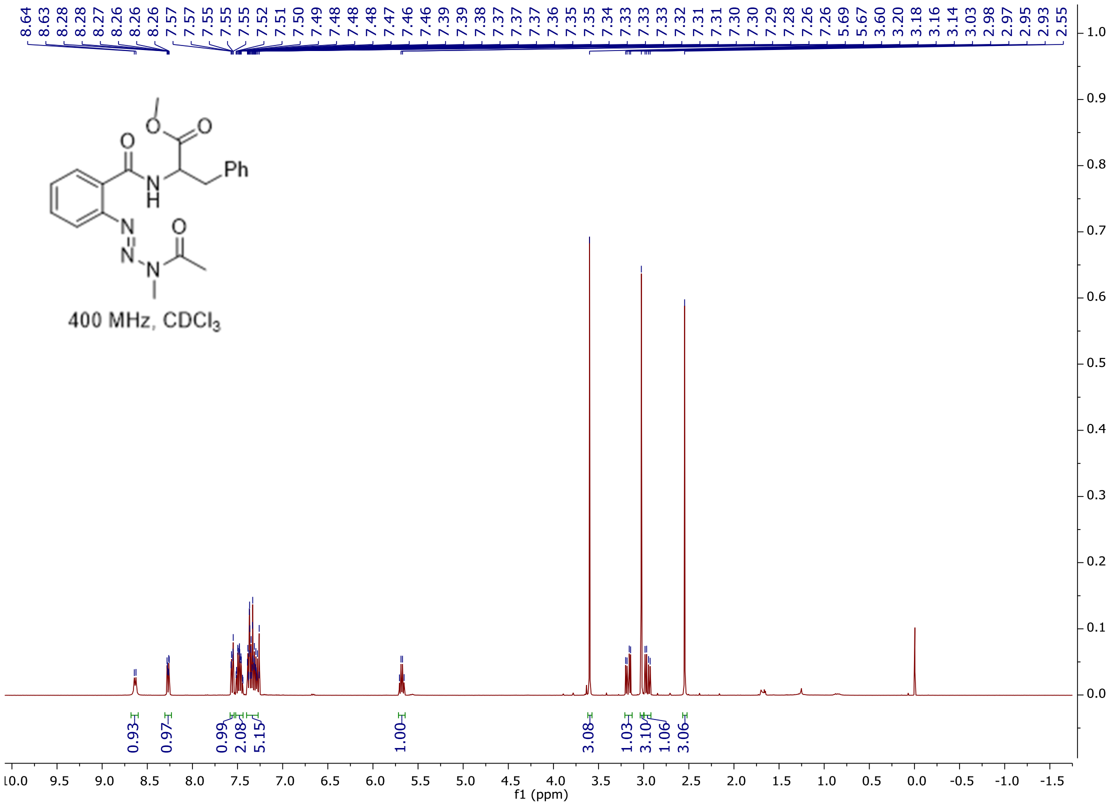

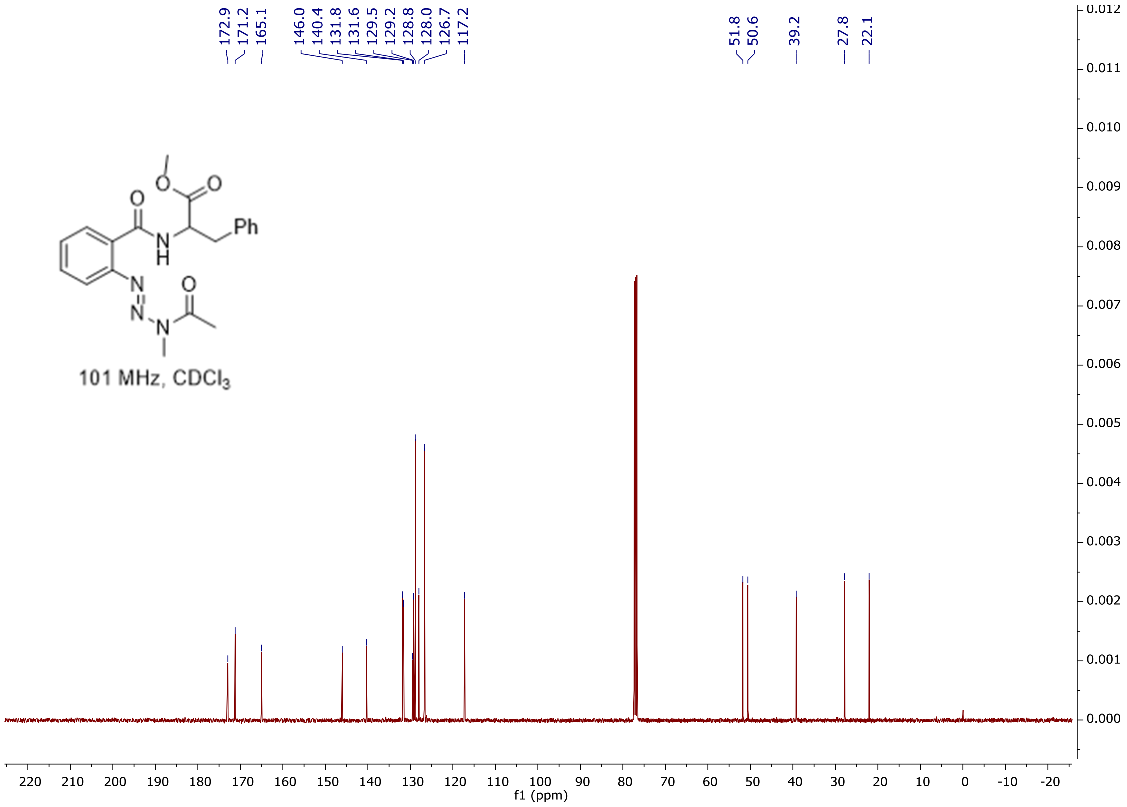

1l: methyl (*E*)-3-(3-acetyl-3-methyltriaz-1-en-1-yl)-4-(cyclopentylcarbamoyl)benzoate

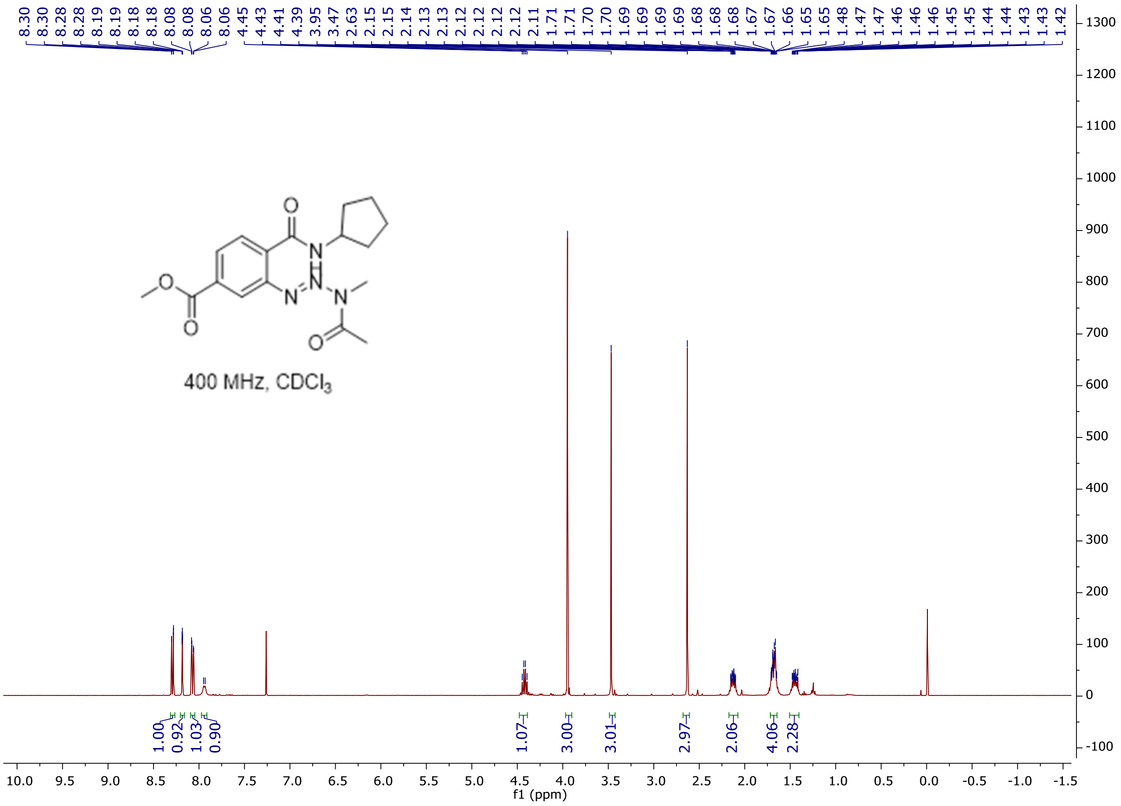

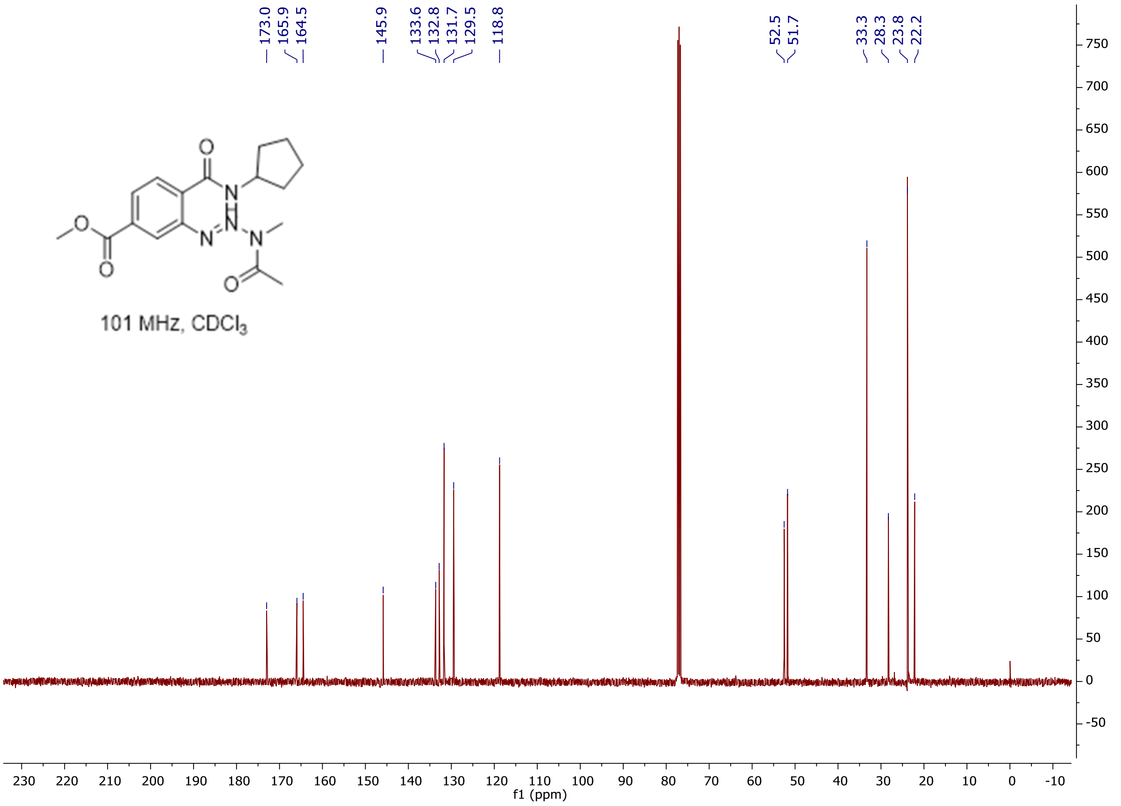

1m: (*E*)-6-(3-acetyl-3-methyltriaz-1-en-1-yl)-*N*-isobutylbenzo[*d*][1,3]dioxole-5-carboxamide

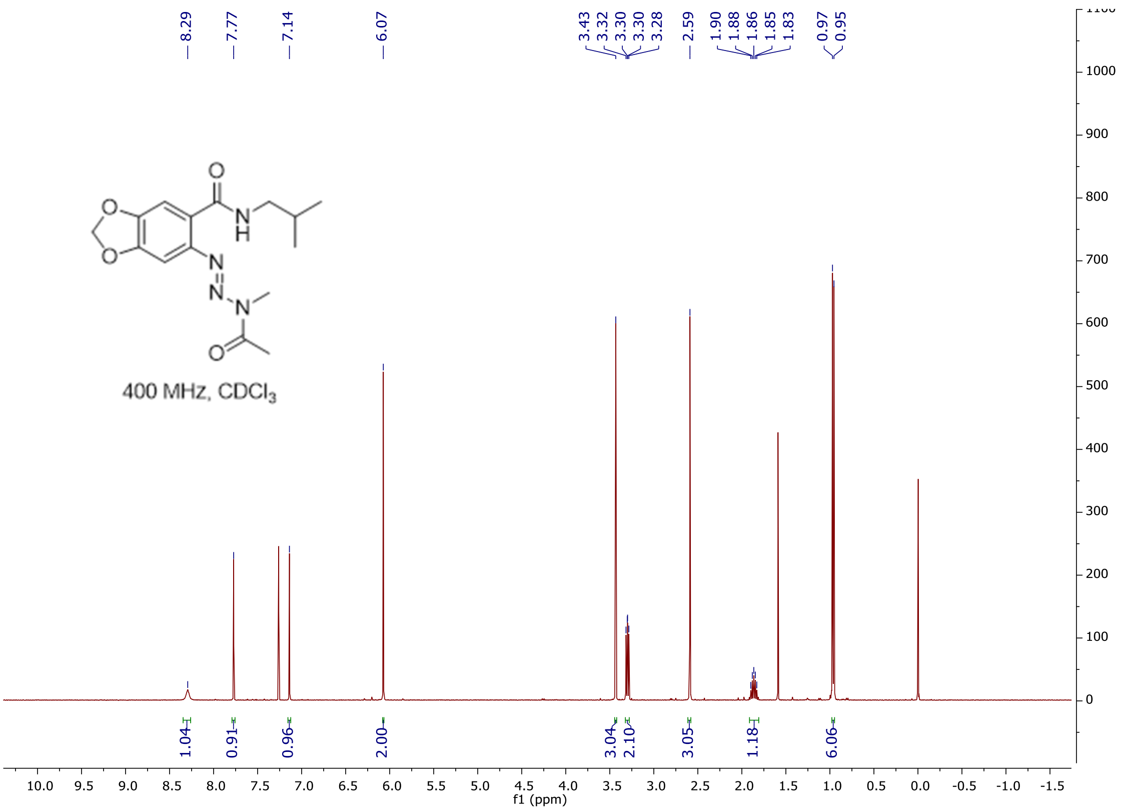

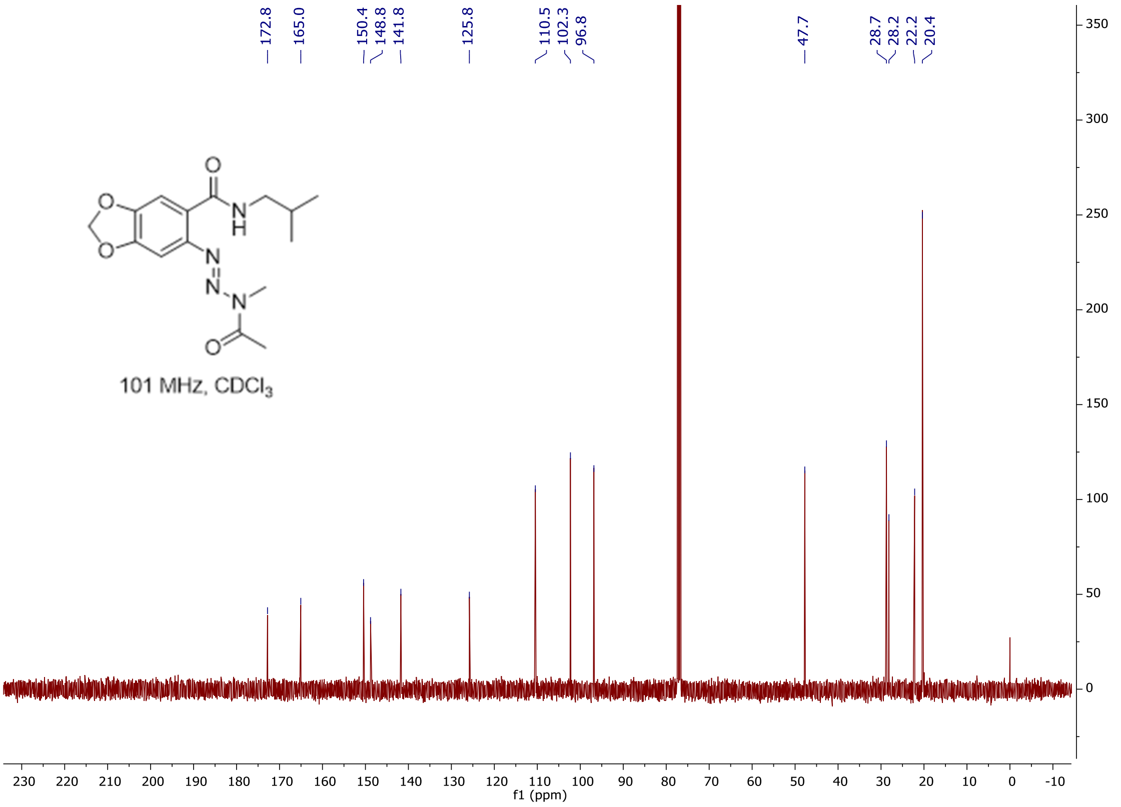

1n: (*E*)-2-(3-acetyl-3-methyltriaz-1-en-1-yl)-*N*-benzyl-5-fluorobenzamide

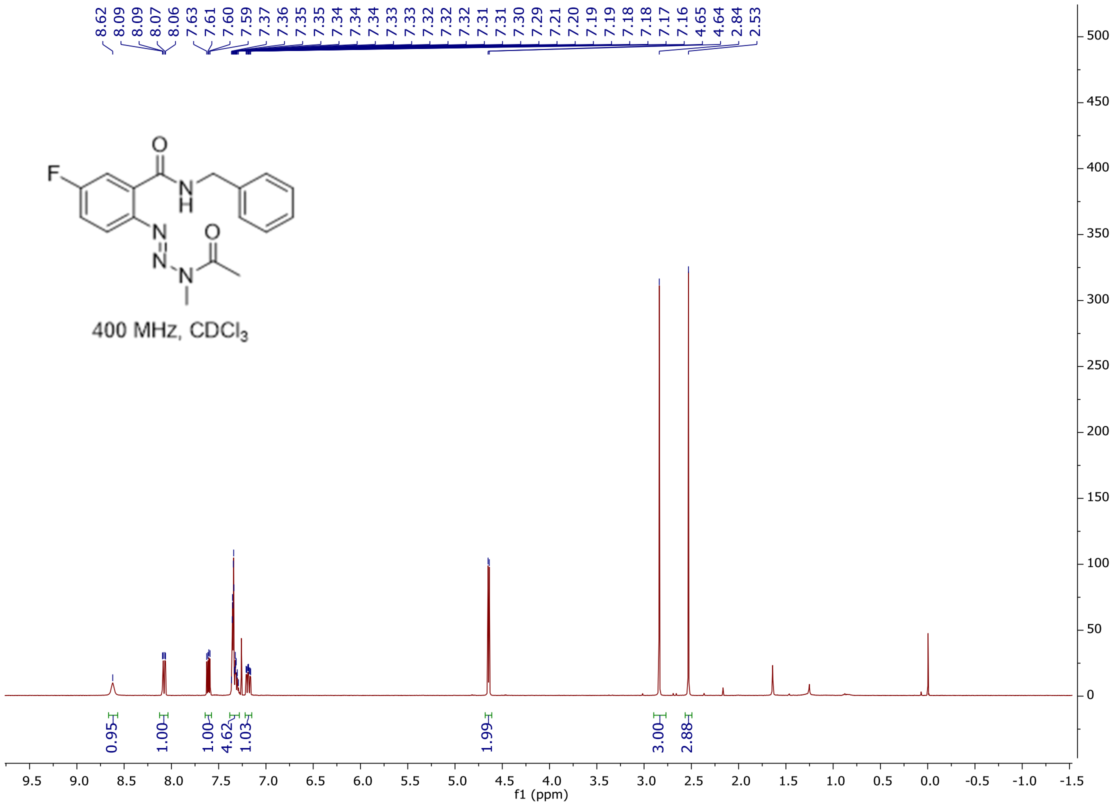

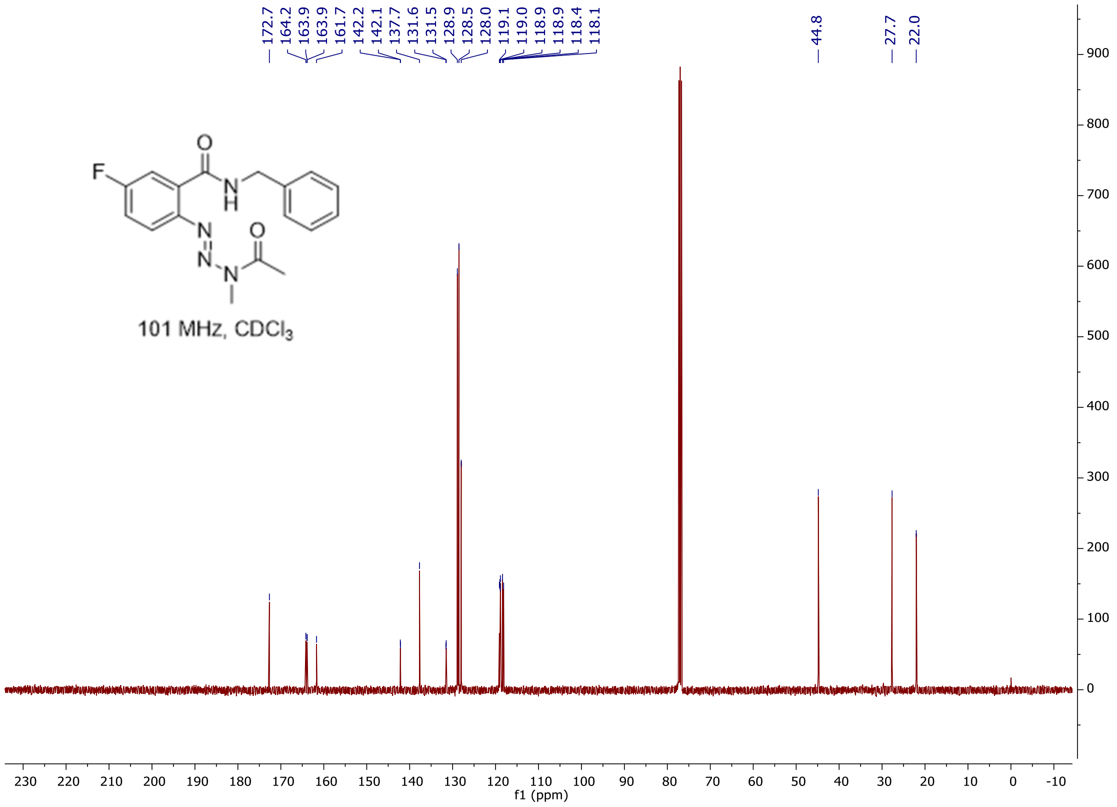

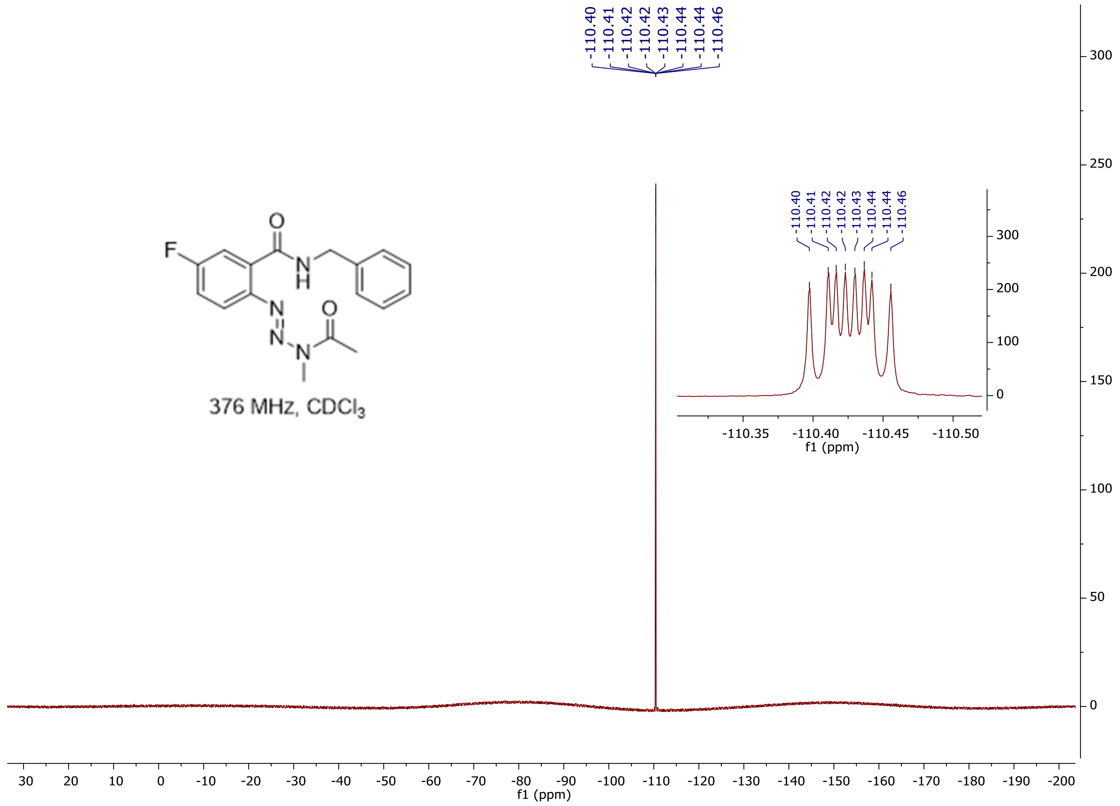
-110.40
-110.41
-110.42
-110.42
-110.43
-110.44
-110.44
-110.46
376 MHz, CDCl3
f1 (ppm)

1o: methyl (*E*)-3-(3-acetyl-3-methyltriaz-1-en-1-yl)-4-((4-isopropylphenyl)carbamoyl)benzoate

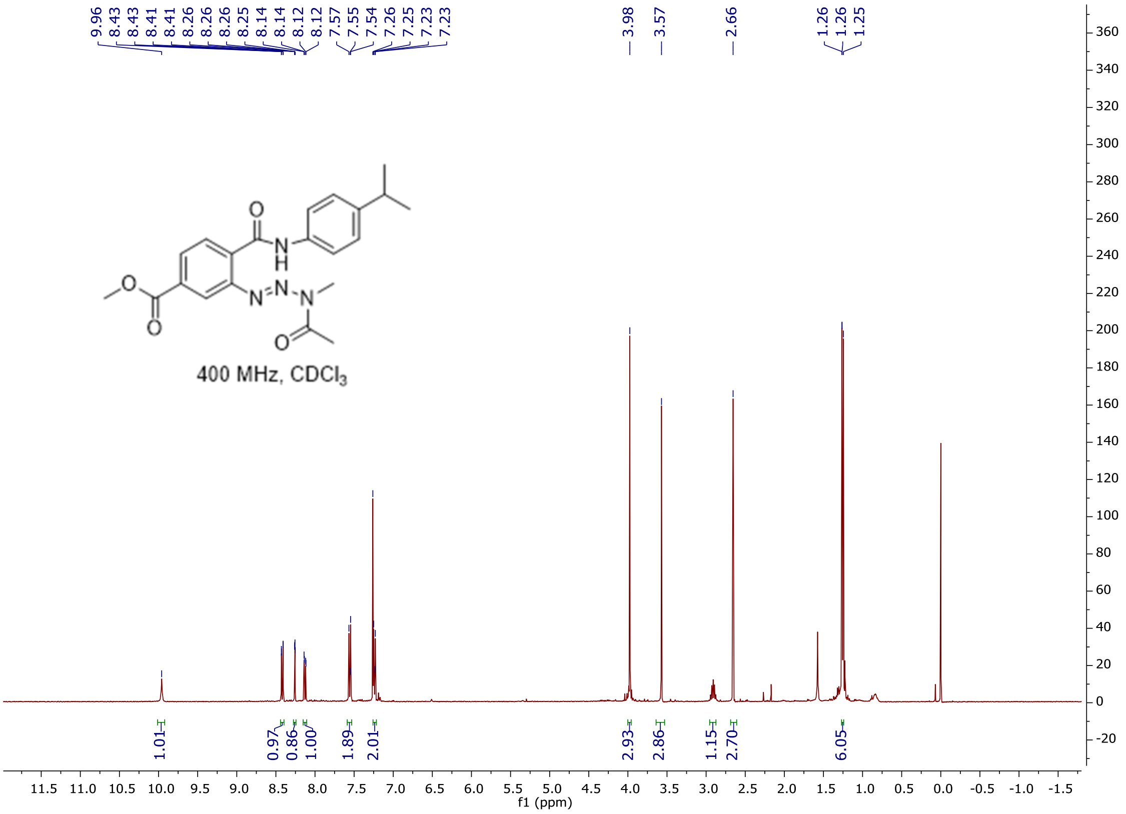

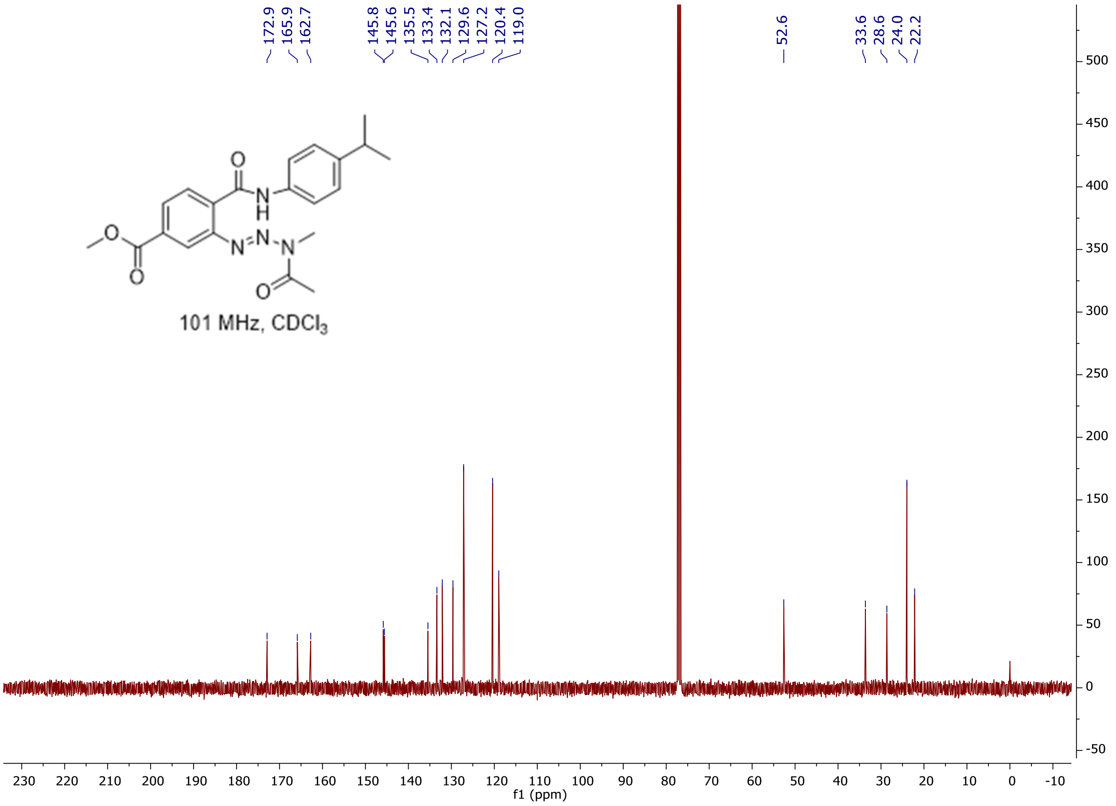

1p: (*E*)-6-(3-acetyl-3-methyltriaz-1-en-1-yl)-*N*-(4-fluorophenyl)benzo[*d*][1,3]dioxole-5-carboxamide

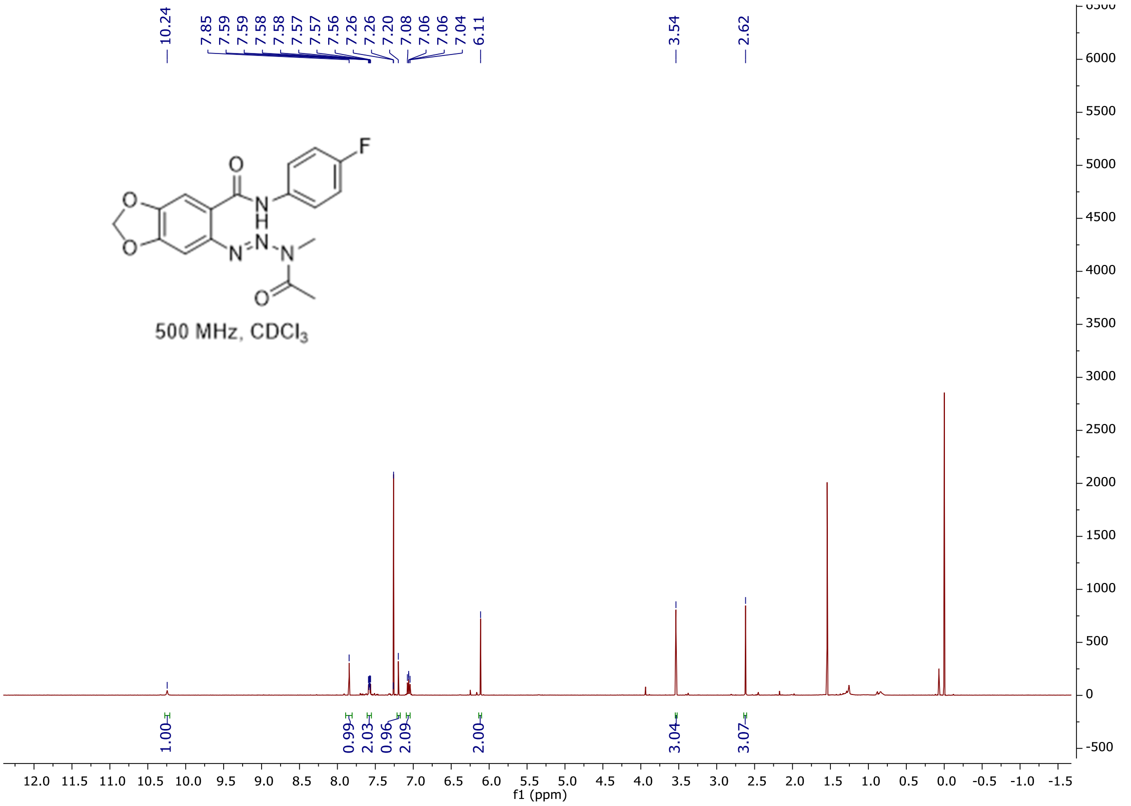

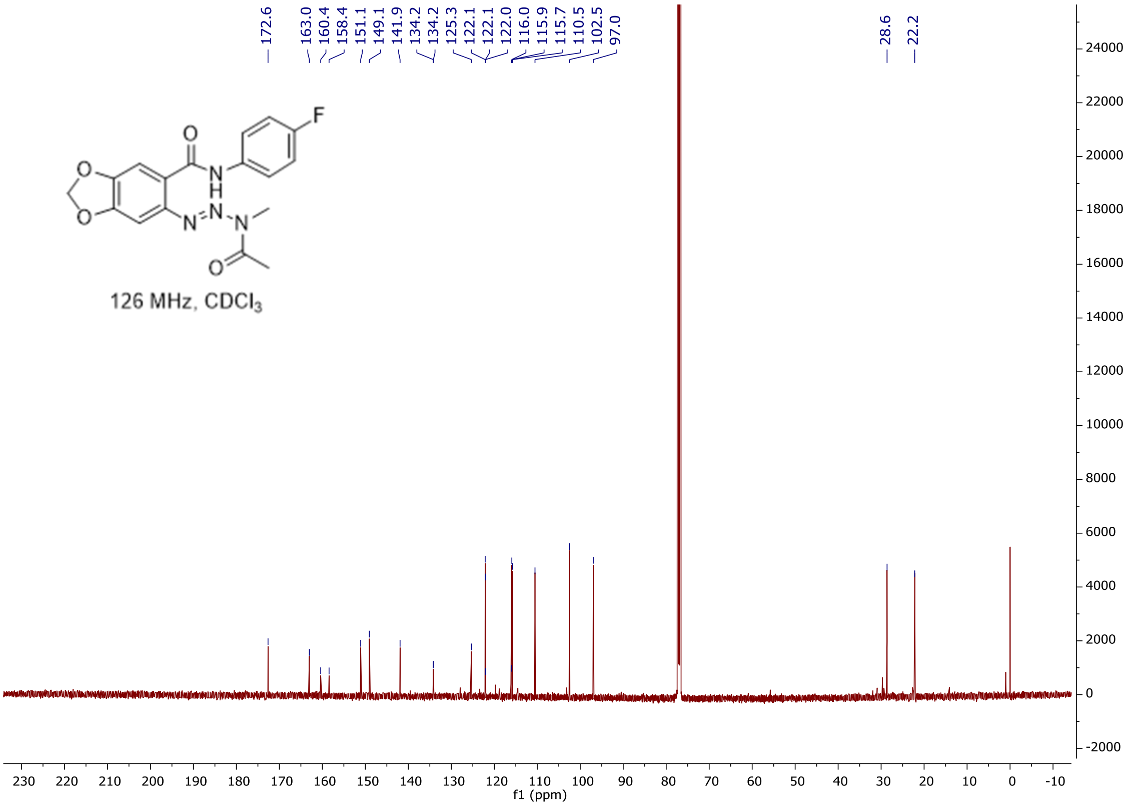

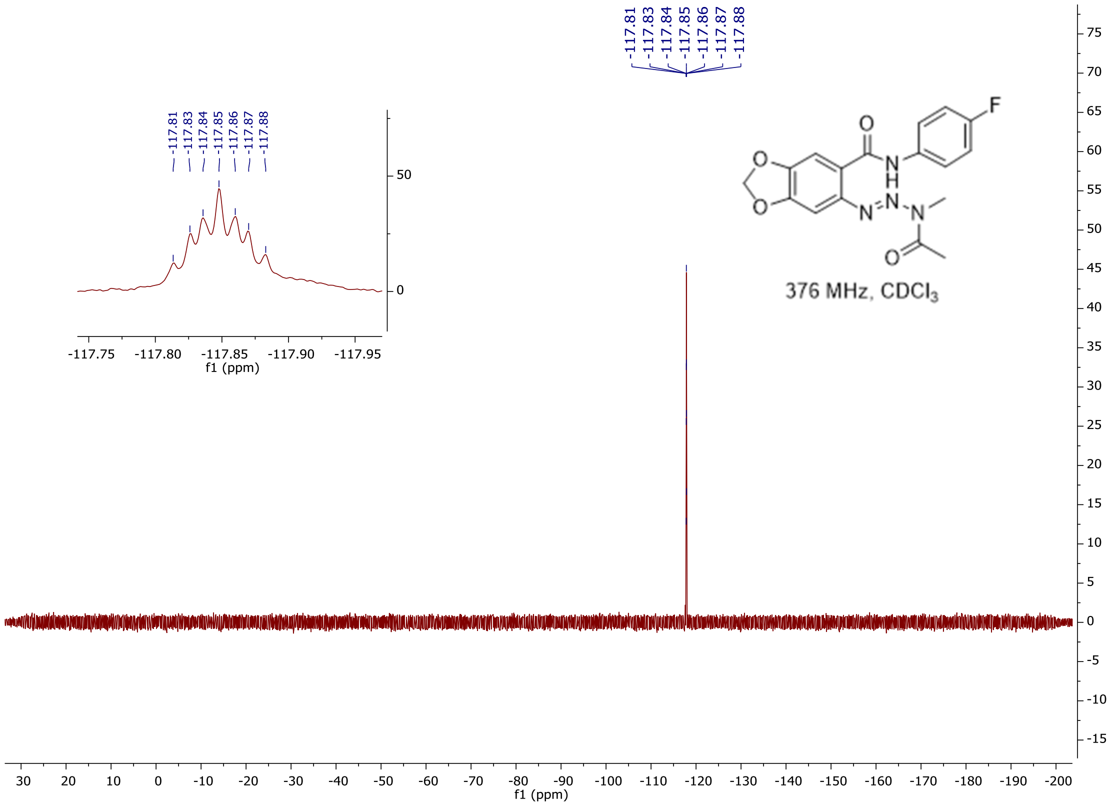

-117.81
-117.83
-117.84
-117.85
-117.86
-117.87
-117.88
376 MHz, CDCl3
f1 (ppm)

1q: (*E*)-2-(3-acetyl-3-benzyltriaz-1-en-1-yl)benzamide

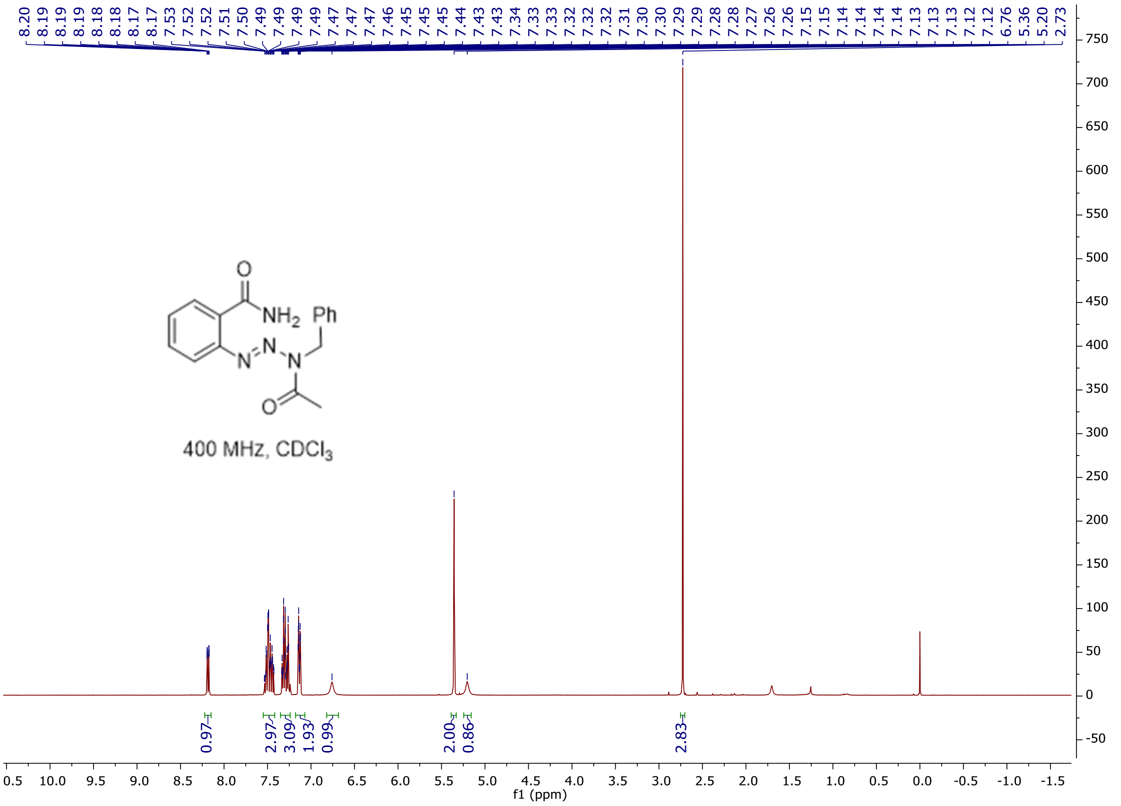

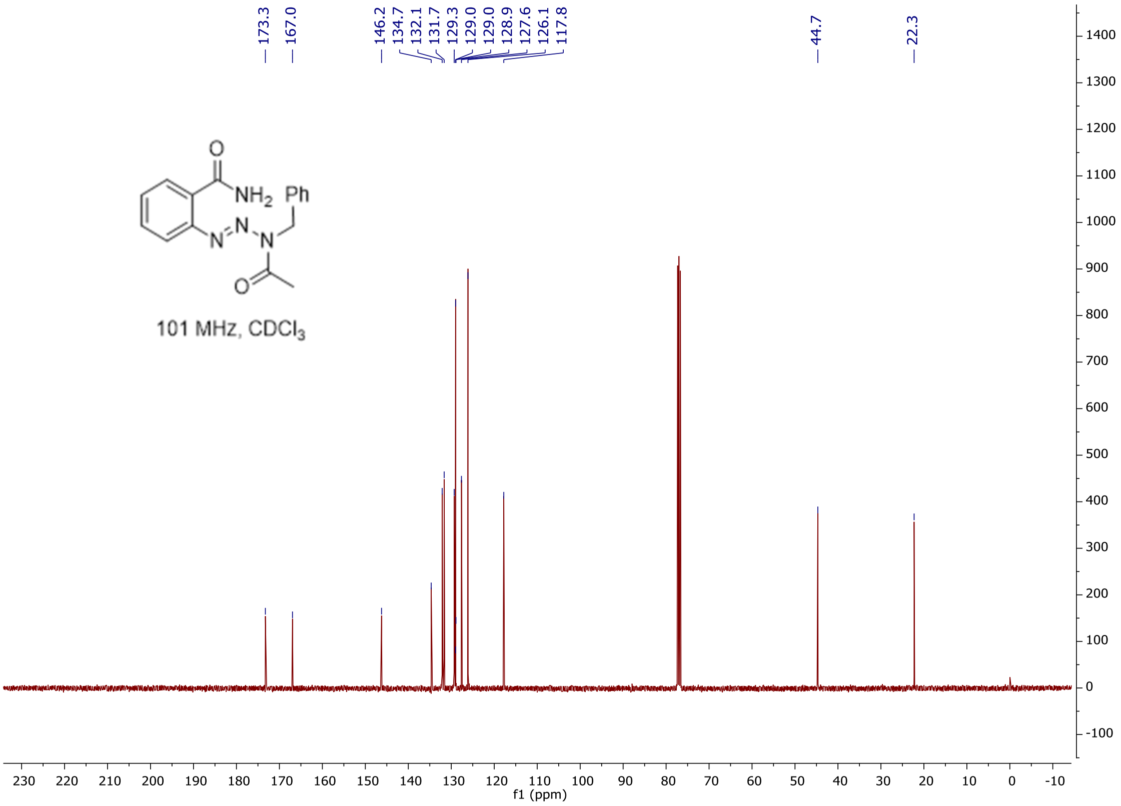

1s: (*E*)-2-(3-acetyl-3-methyltriaz-1-en-1-yl)-*N*,*N*-diethylbenzamide

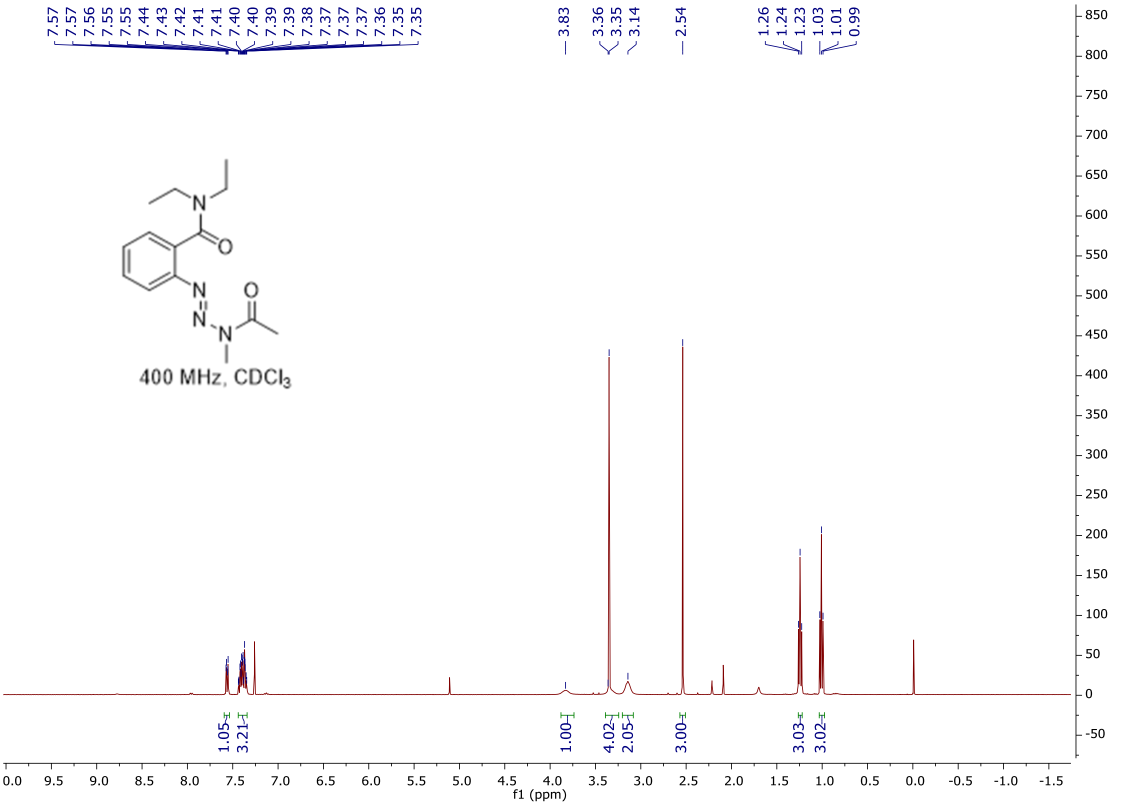

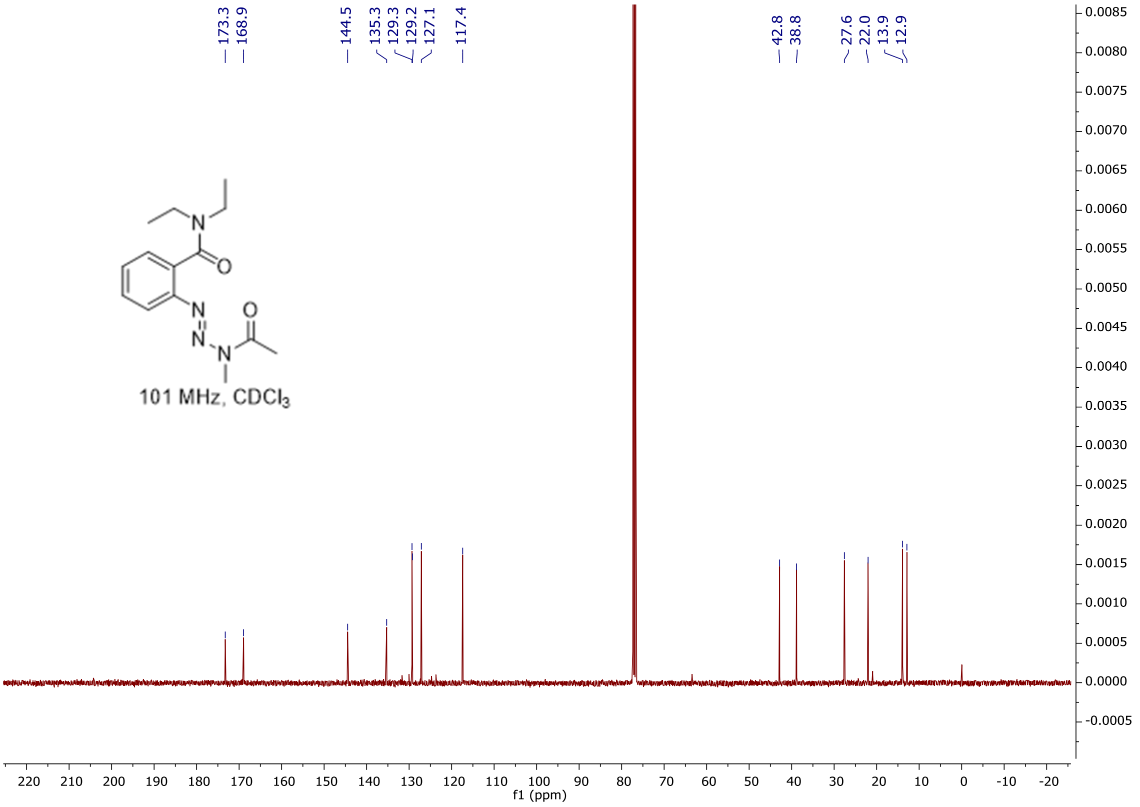

1t: (*E*)-1-(3-(2'-amino-[1,1'-biphenyl]-2-yl)-1-methyltriaz-2-en-1-yl)ethan-1-one

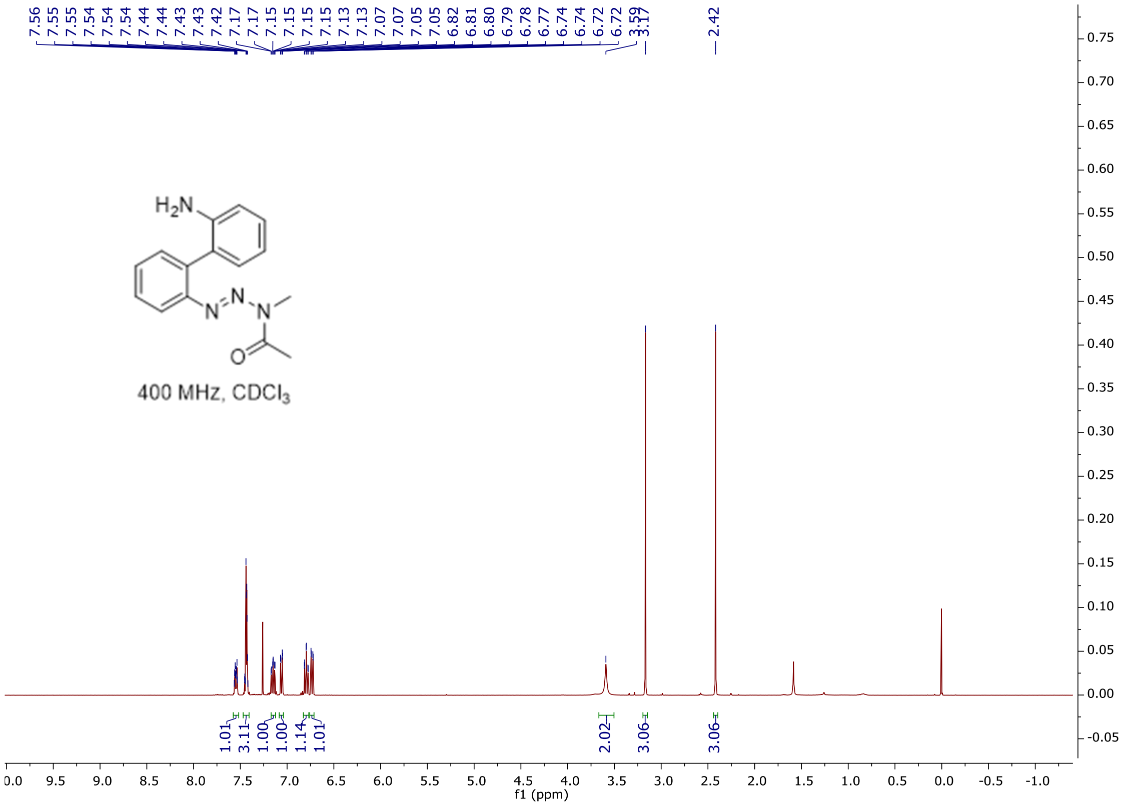

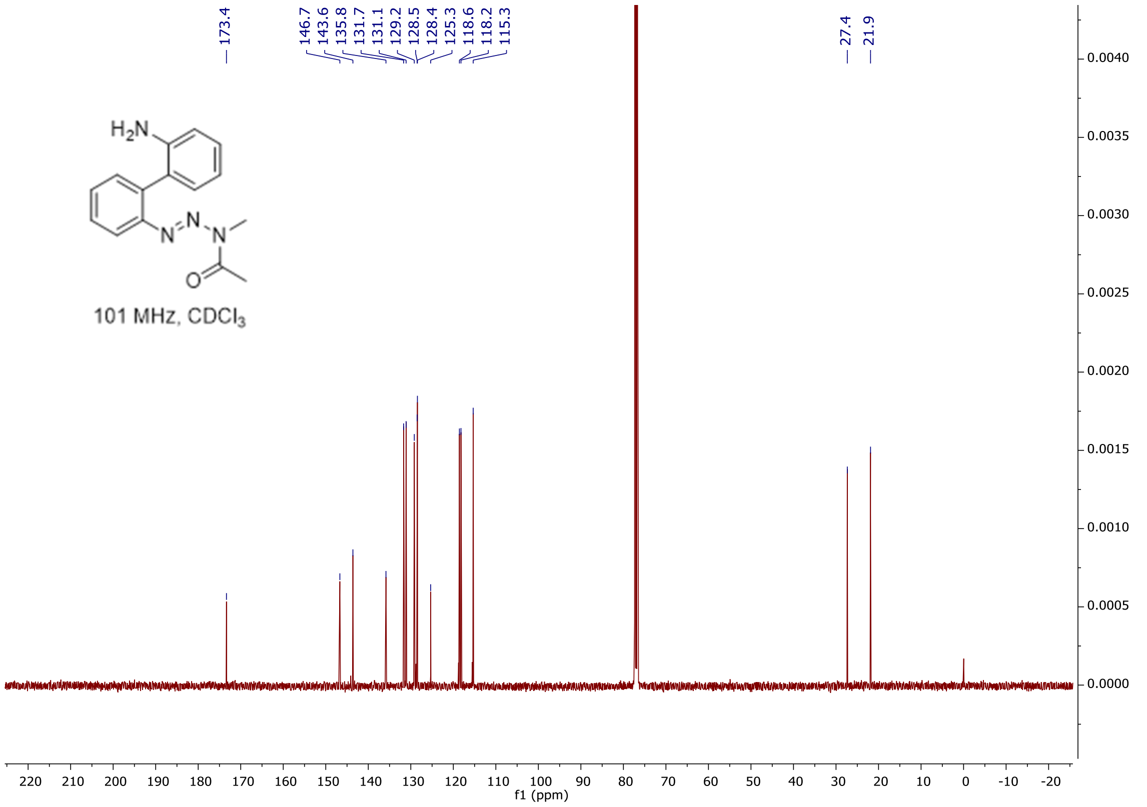

1t': (9*H*-fluoren-9-yl)methyl (2'-amino-[1,1'-biphenyl]-2-yl)carbamate

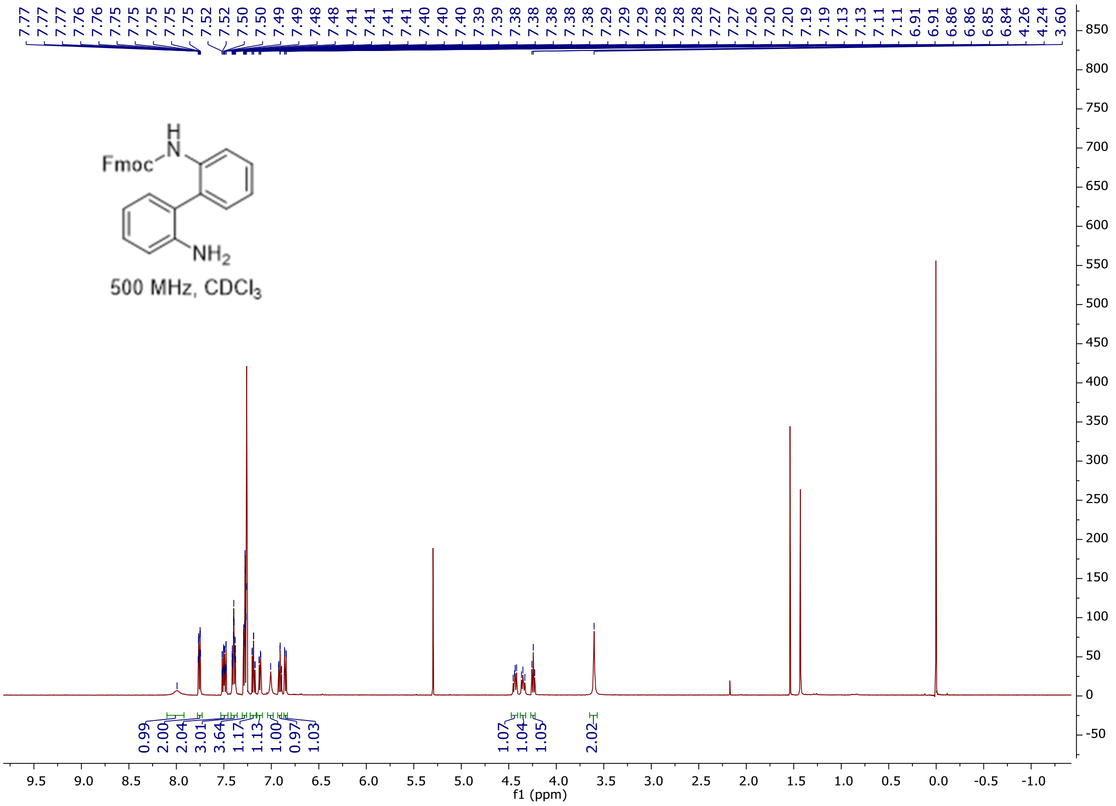

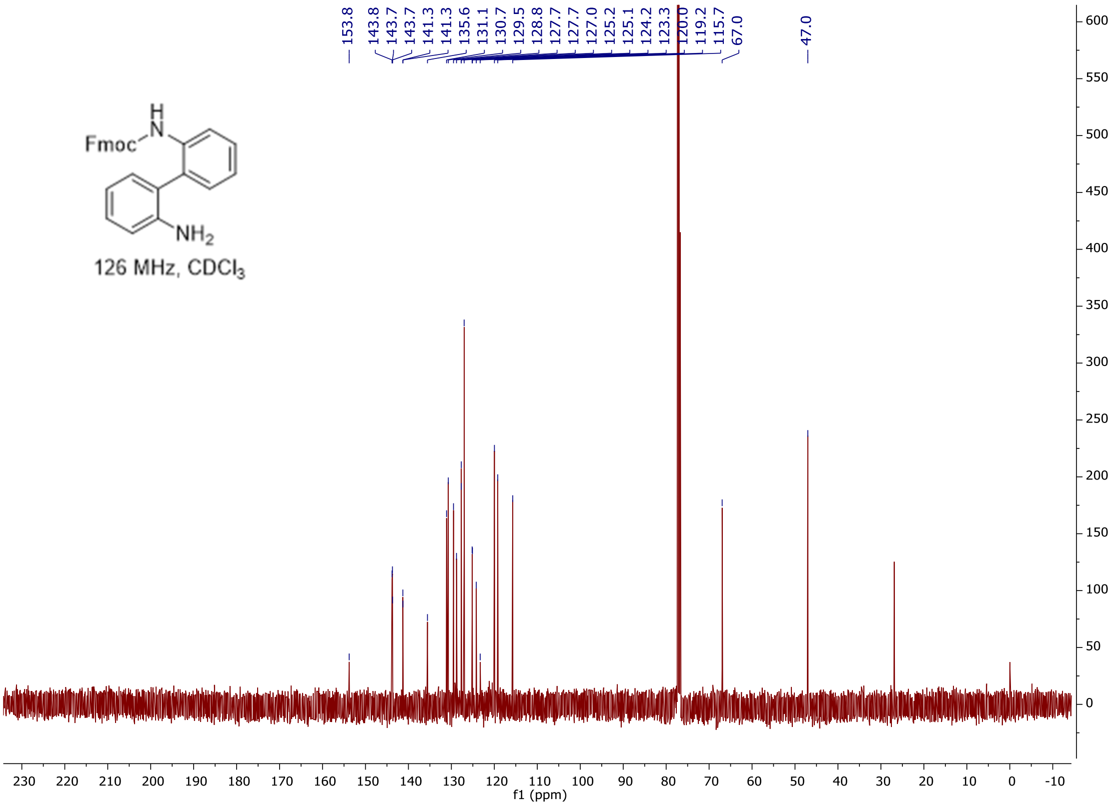

NMR copies of Benzotriazin-4(3*H*)-ones (**2a-2p**)

2a: benzo[*d*][1,2,3]triazin-4(3*H*)-one

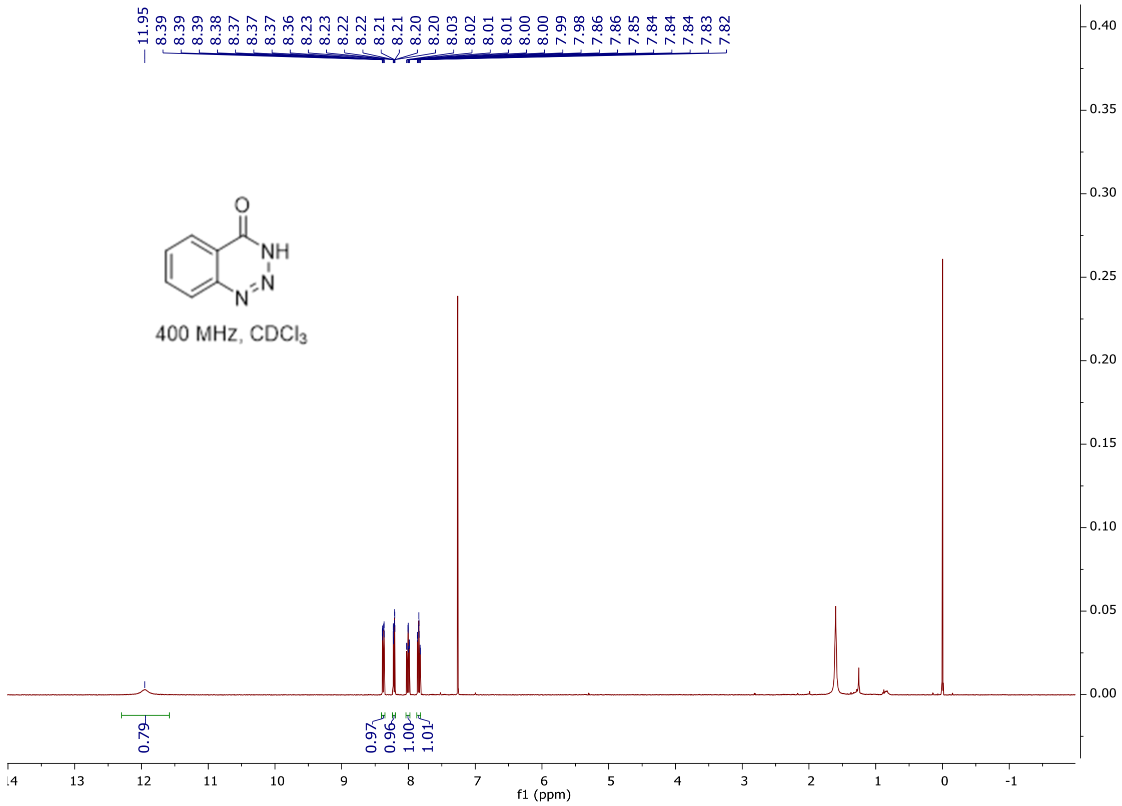

11.95
8.39
8.39
8.39
8.38
8.37
8.37
8.37
8.36
8.23
8.23
8.22
8.22
8.21
8.21
8.20
8.03
8.02
8.01
8.00
8.00
7.99
7.98
7.86
7.85
7.84
7.84
7.84
7.83
7.82
NH
N
N
O
400 MHz, CDCl3
0.79
0.97
0.96
1.00
1.01
f1 (ppm)

2b: 6-fluorobenzo[*d*][1,2,3]triazin-4(3*H*)-one

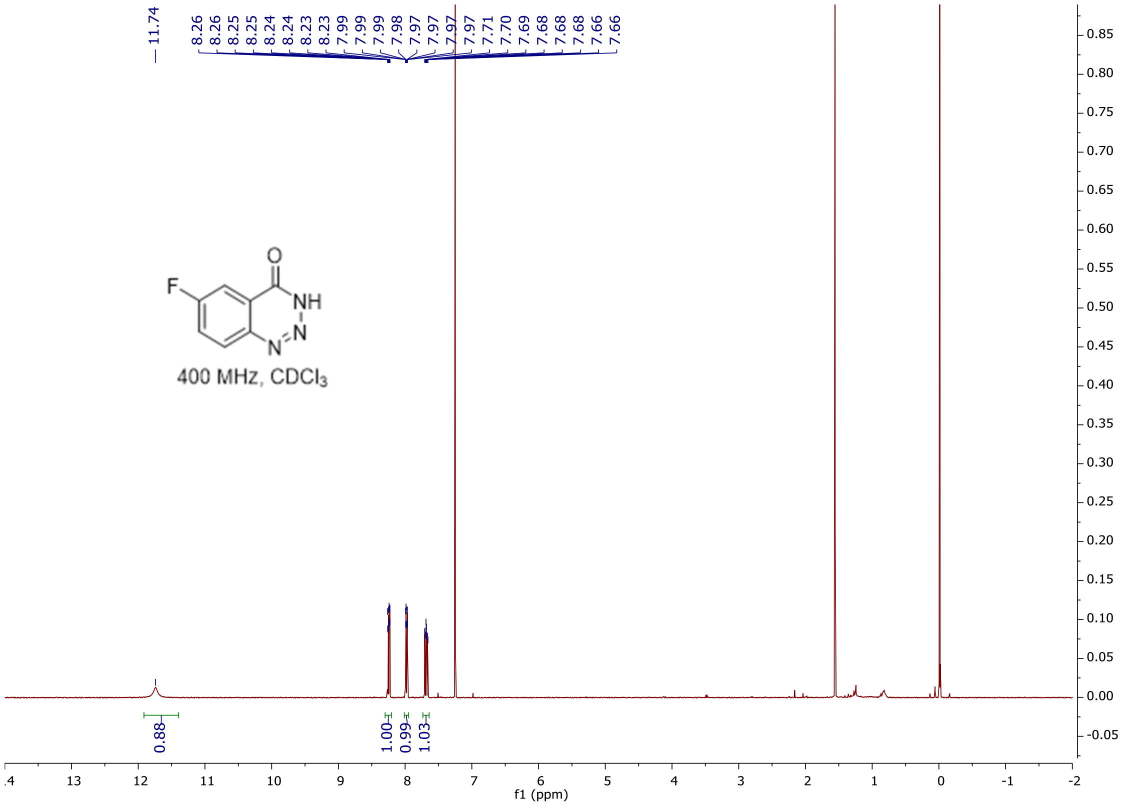

2c: methyl 4-oxo-3,4-dihydrobenzo[*d*][1,2,3]triazine-7-carboxylate

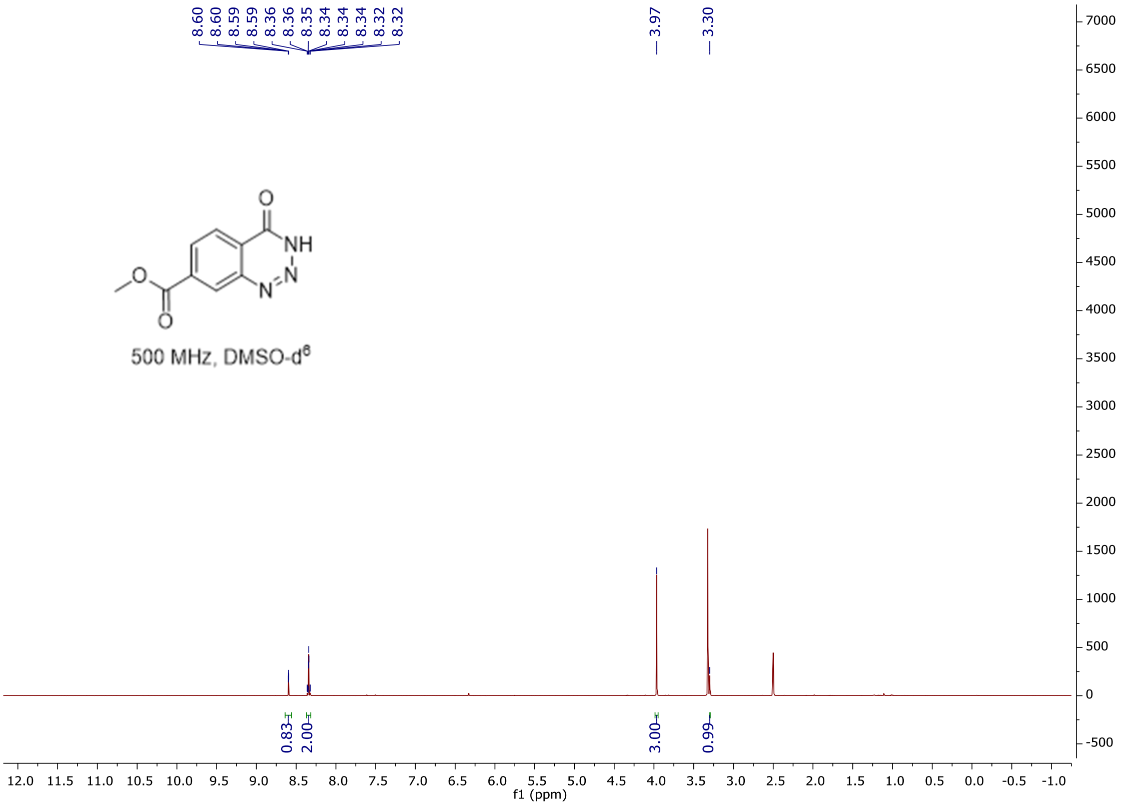

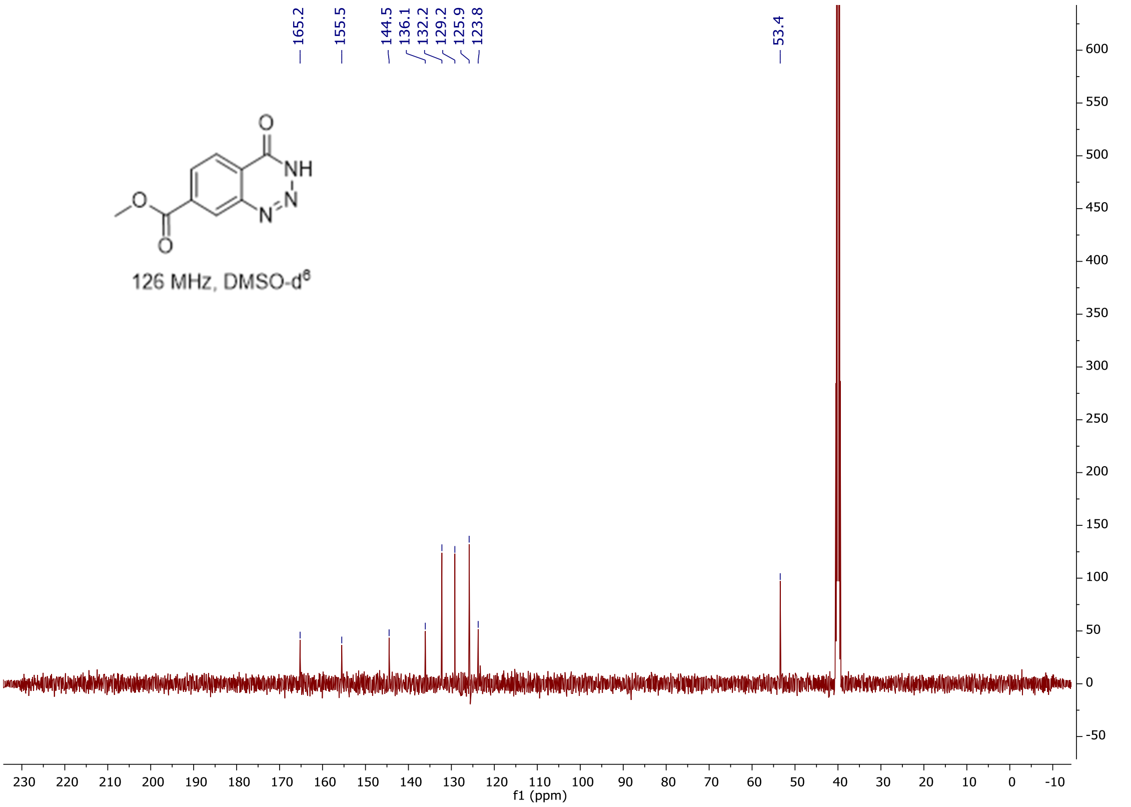

2d: 6-methoxybenzo[*d*][1,2,3]triazin-4(3*H*)-one

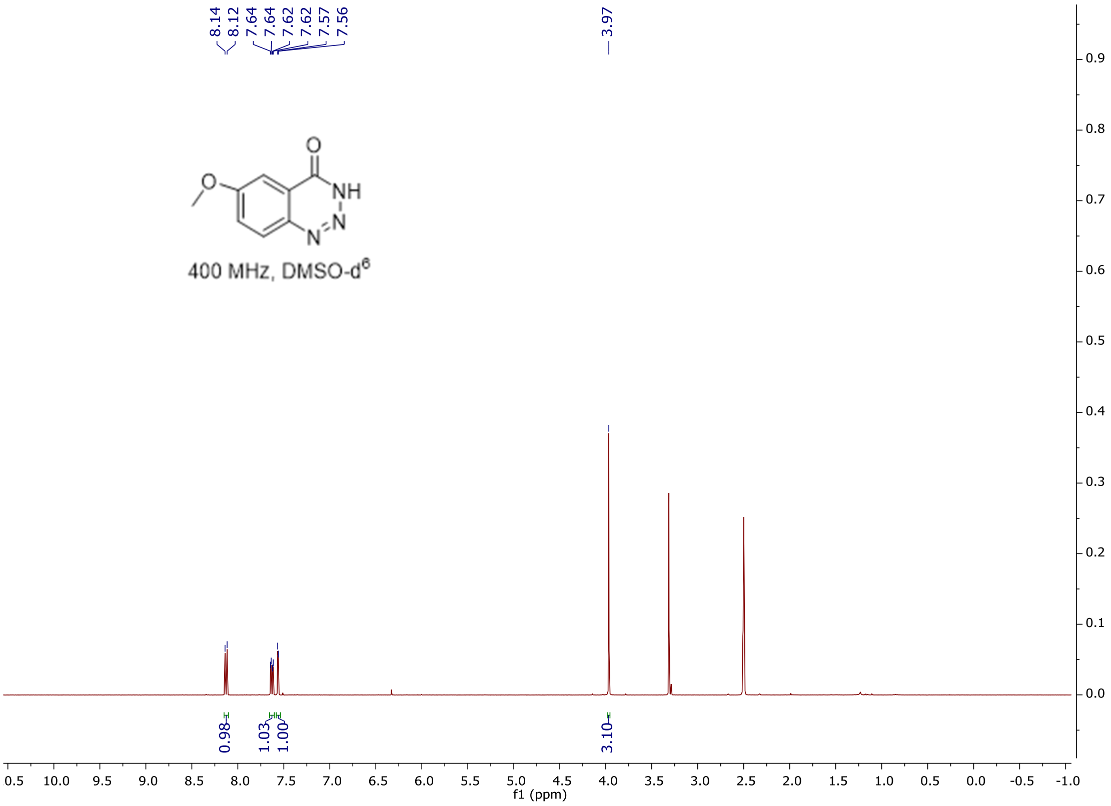

2e: [1,3]dioxolo[4',5':4,5]benzo[1,2-*d*][1,2,3]triazin-4(3*H*)-one

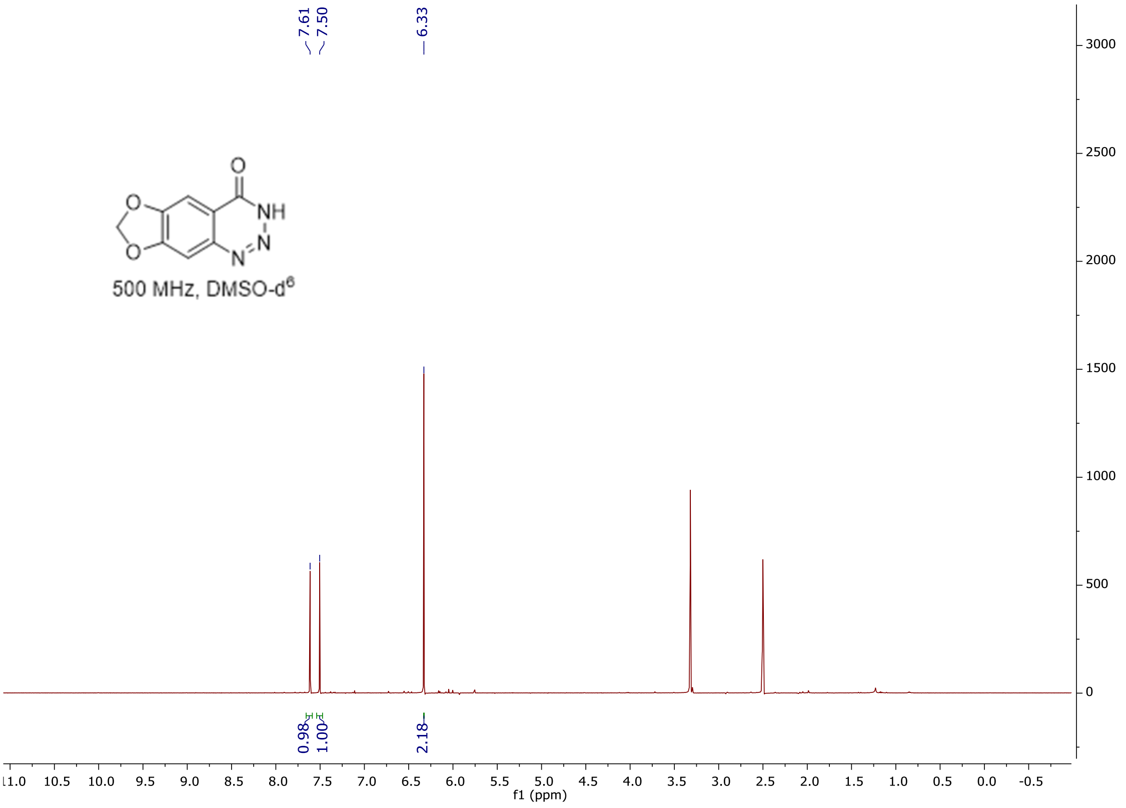

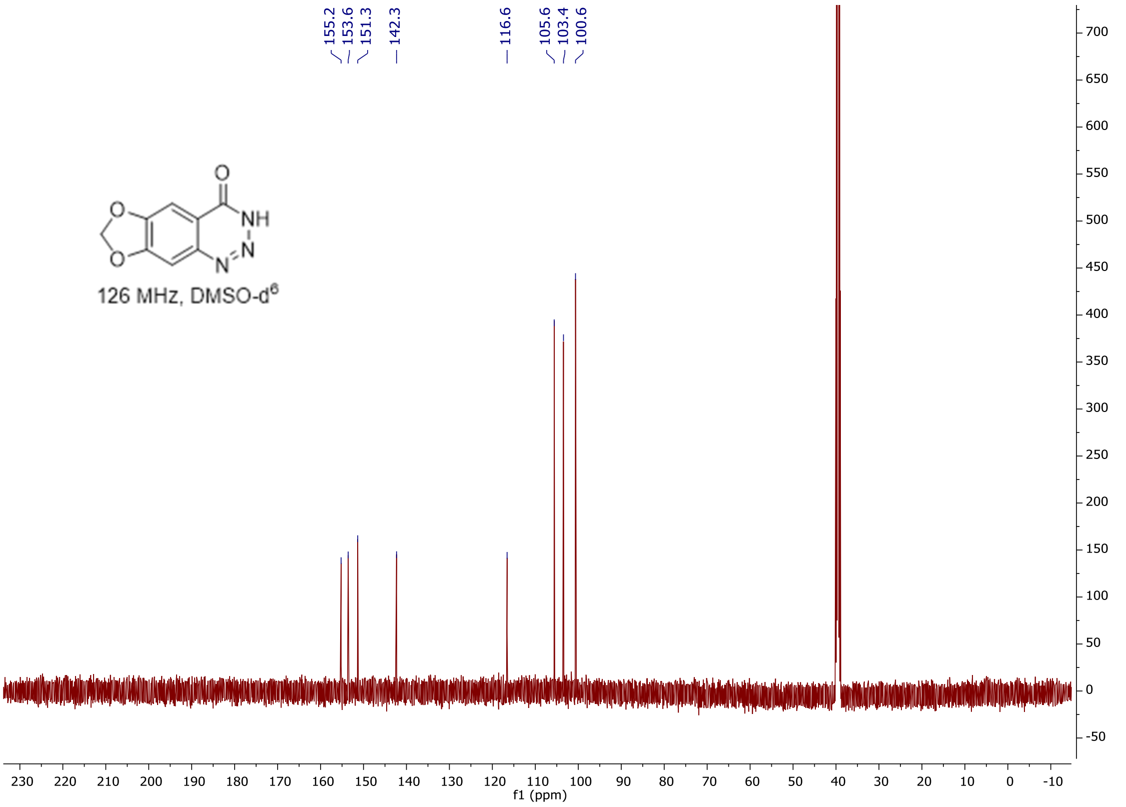

2f: 3-cyclopentylbenzo[*d*][1,2,3]triazin-4(3*H*)-one

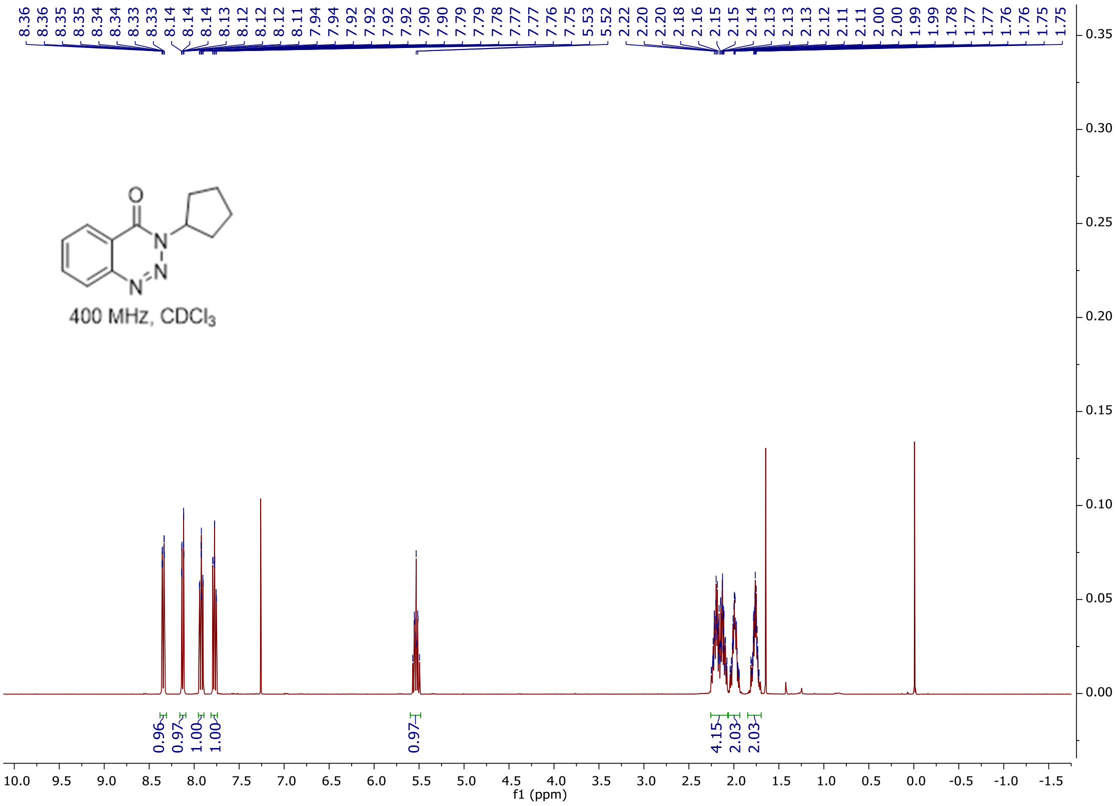

2g: 3-isobutylbenzo[*d*][1,2,3]triazin-4(3*H*)-one

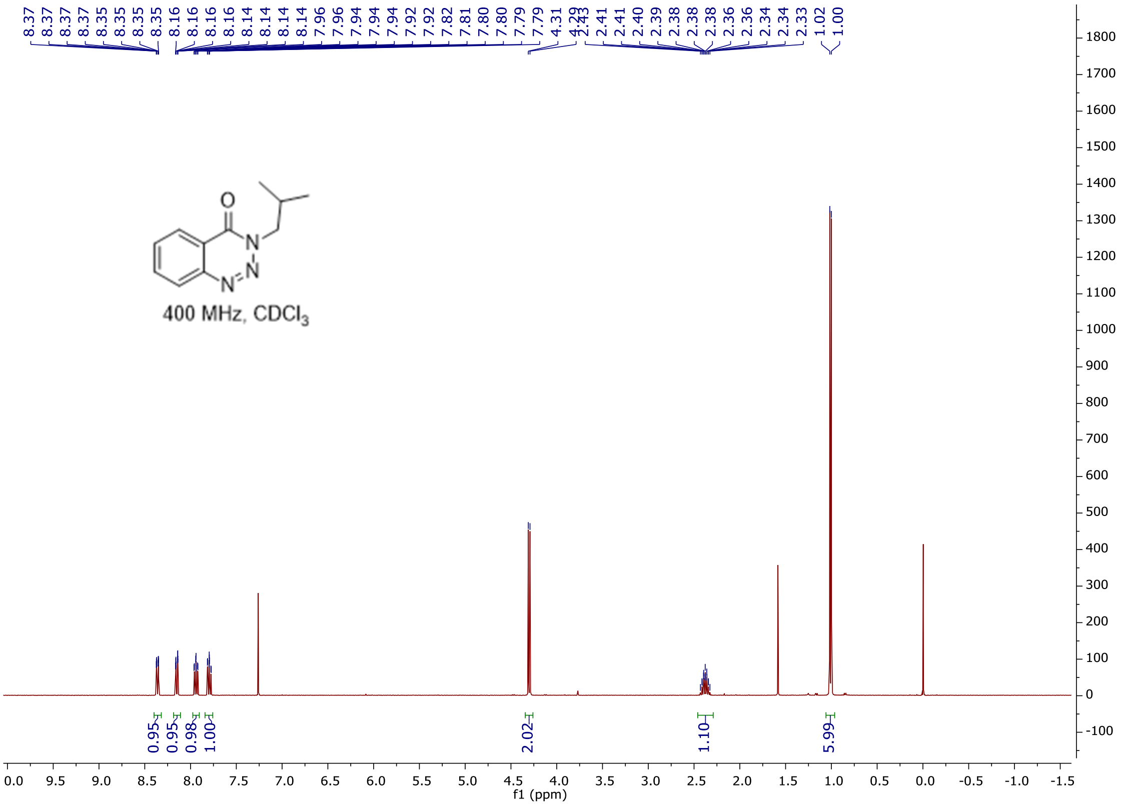

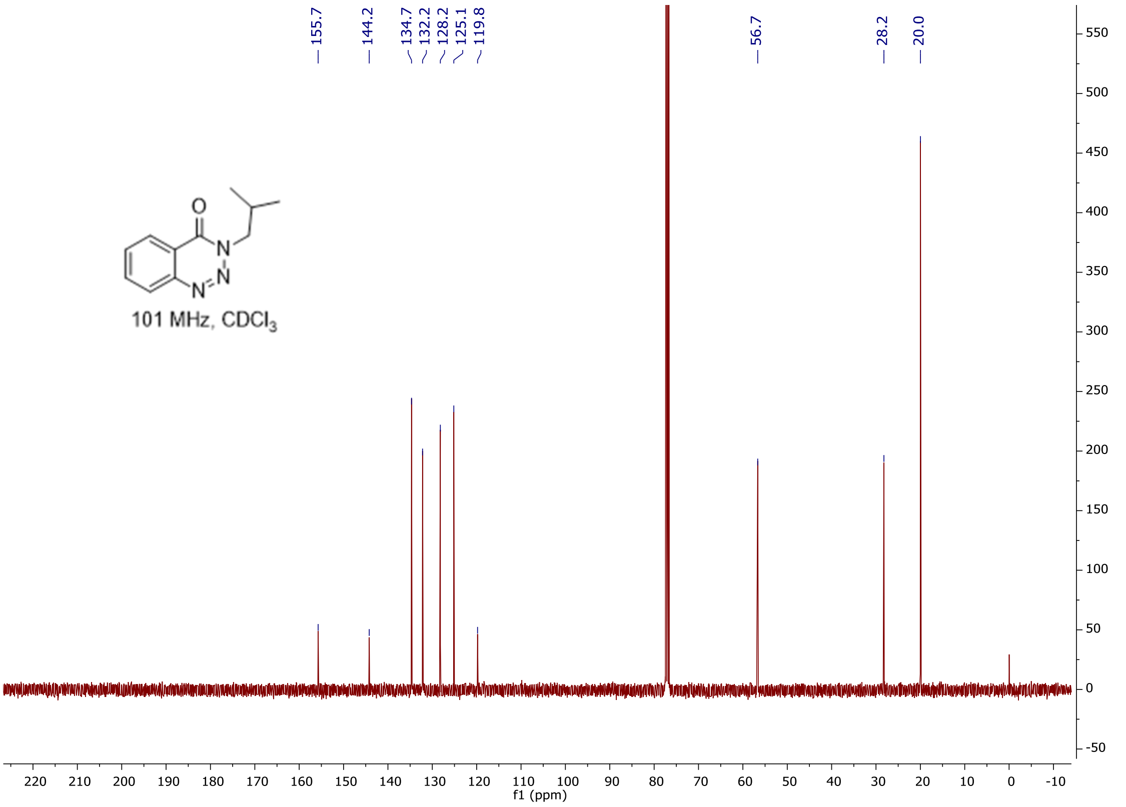

2h: 3-(furan-2-ylmethyl)benzo[*d*][1,2,3]triazin-4(3*H*)-one

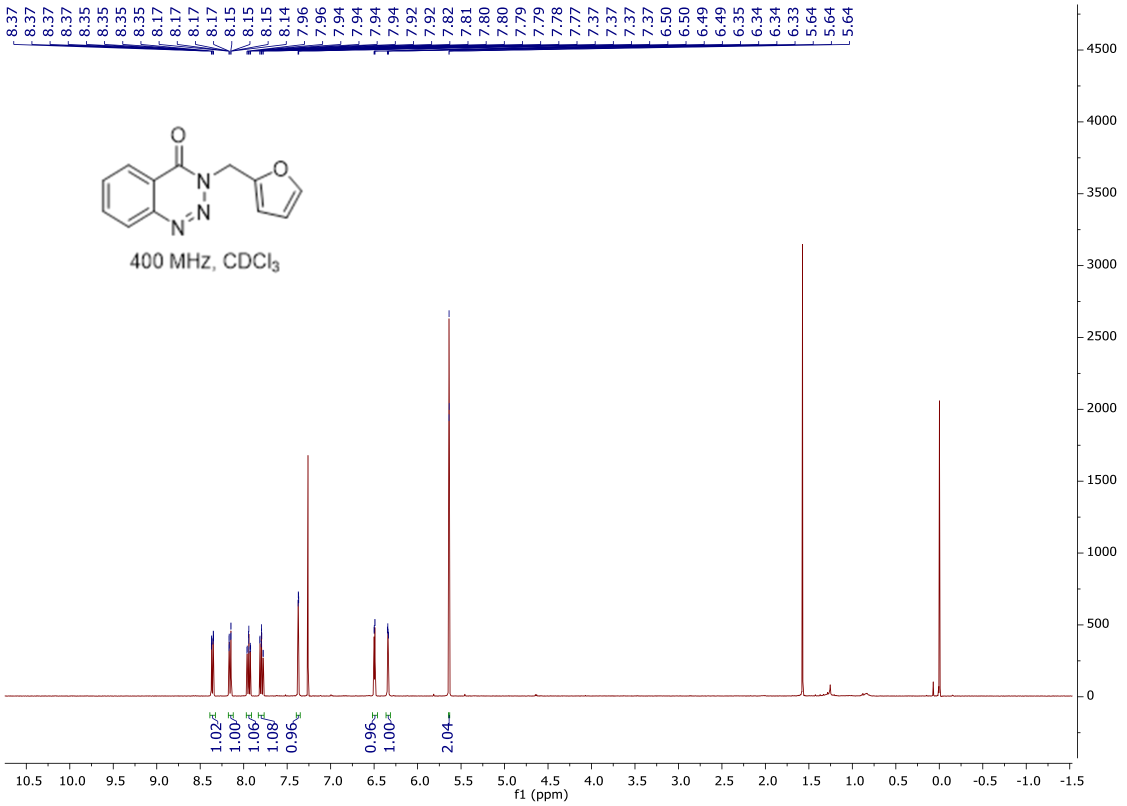

2i: 3-(2-methoxybenzyl)benzo[*d*][1,2,3]triazin-4(3*H*)-one

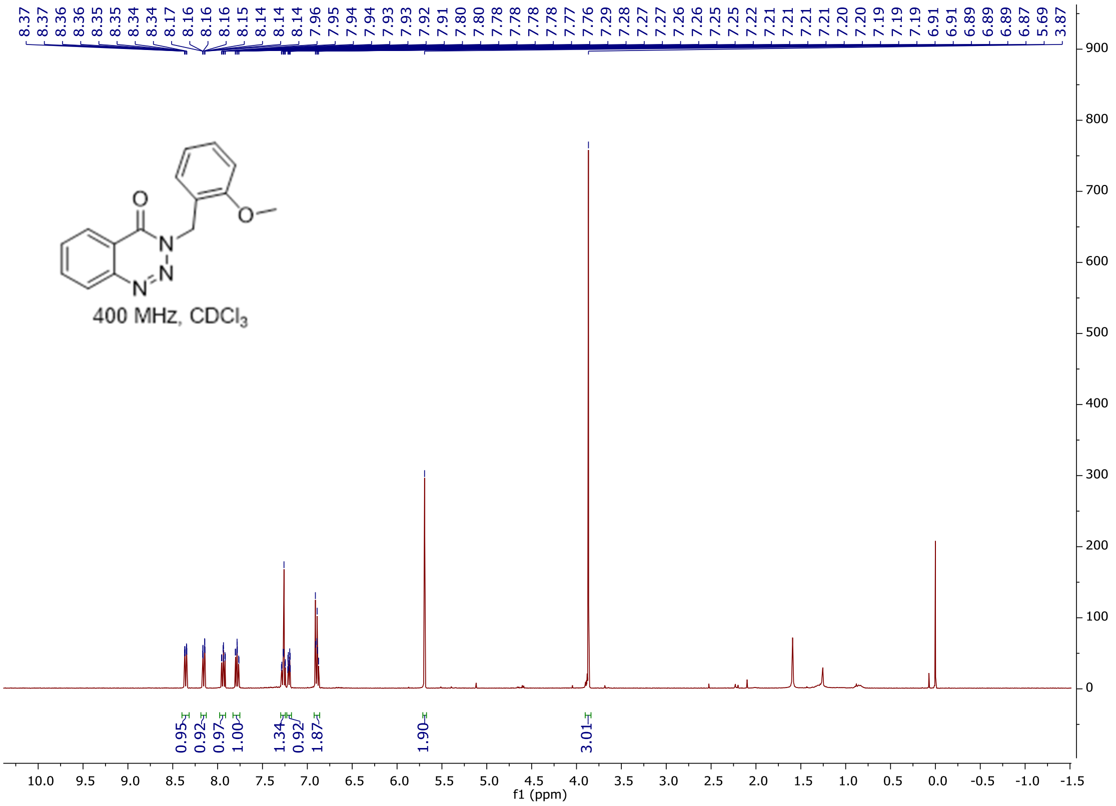

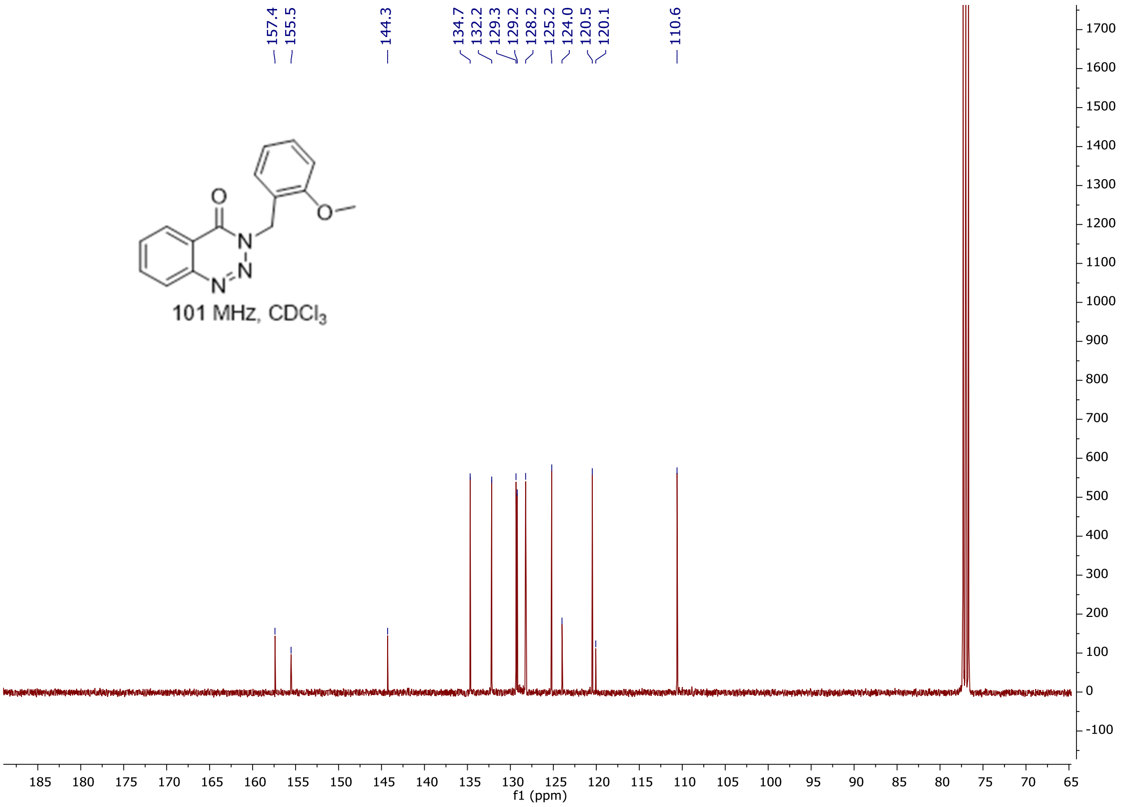

2j: 3-phenylbenzo[*d*][1,2,3]triazin-4(3*H*)-one

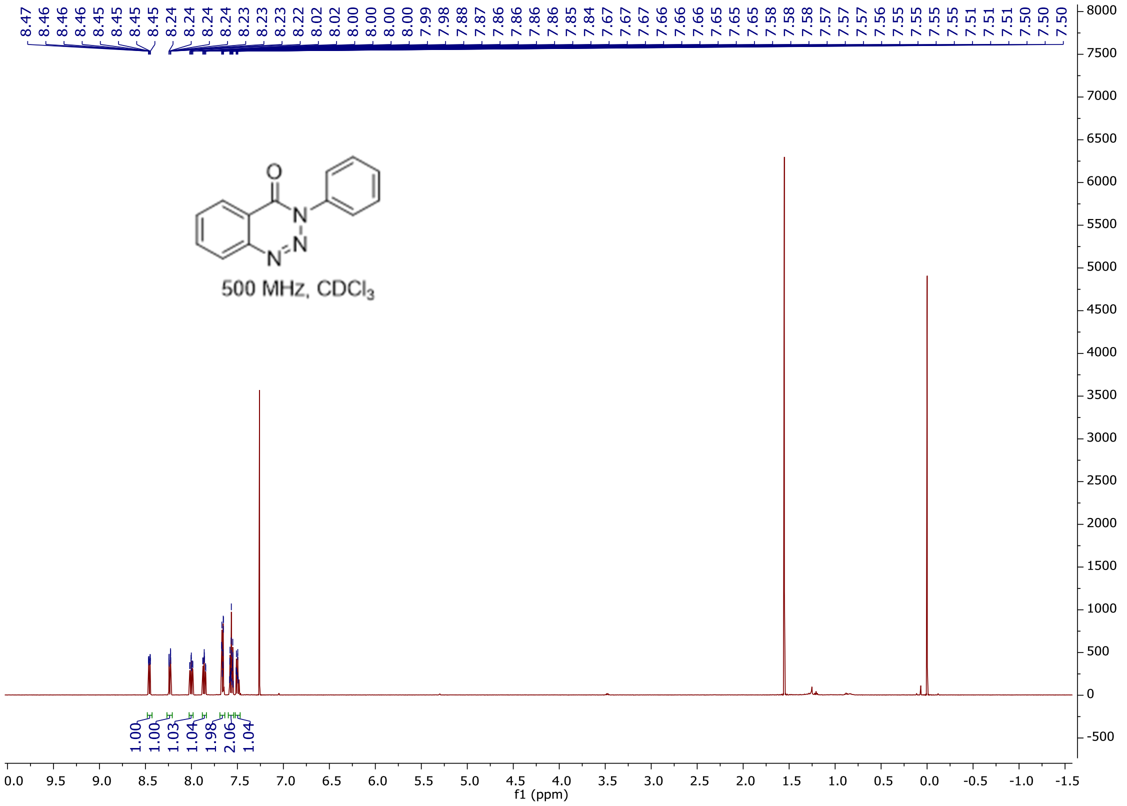

2k: *rac*-methyl 2-(4-oxobenzo[*d*][1,2,3]triazin-3(4*H*)-yl)-3-phenylpropanoate

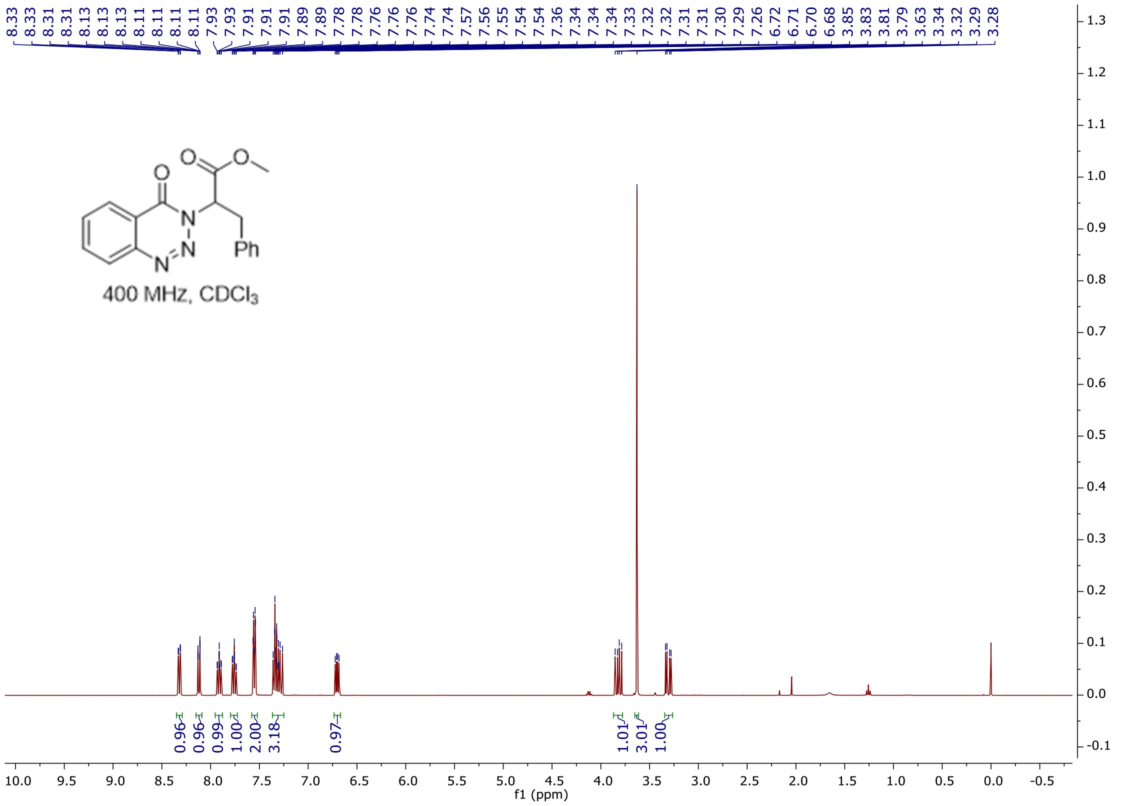

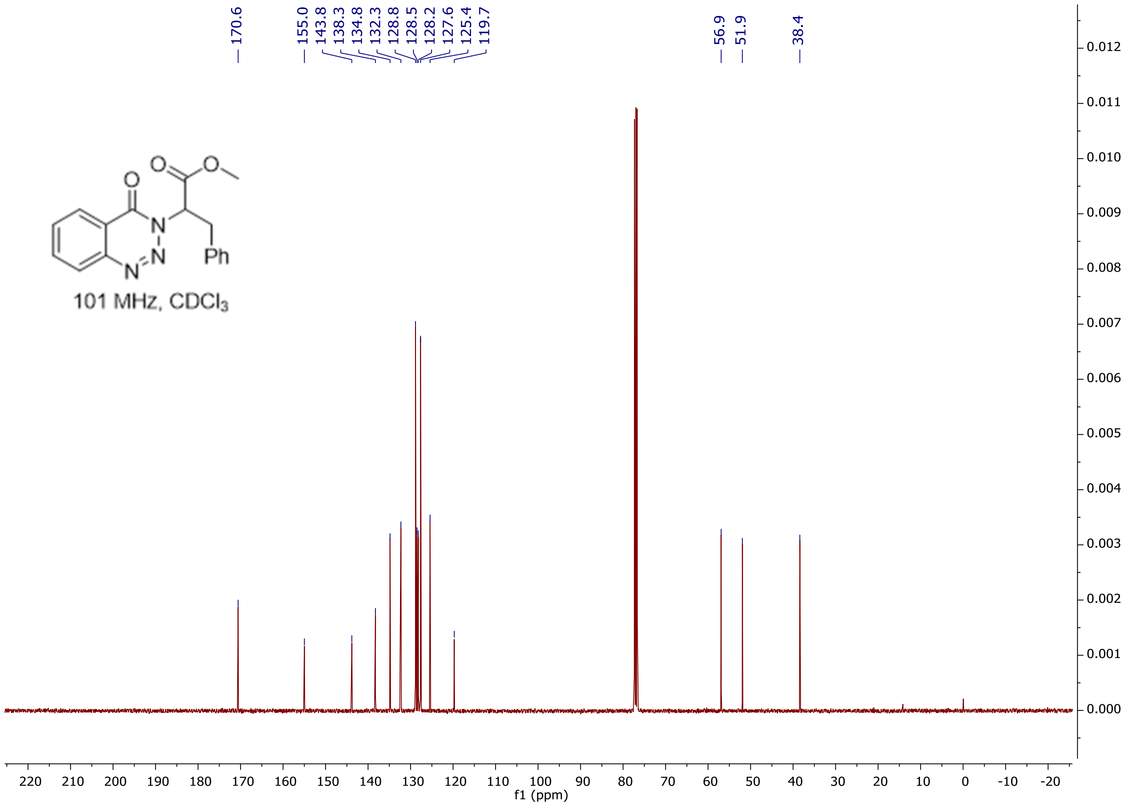

2l: methyl 3-cyclopentyl-4-oxo-3,4-dihydrobenzo[*d*][1,2,3]triazine-7-carboxylate

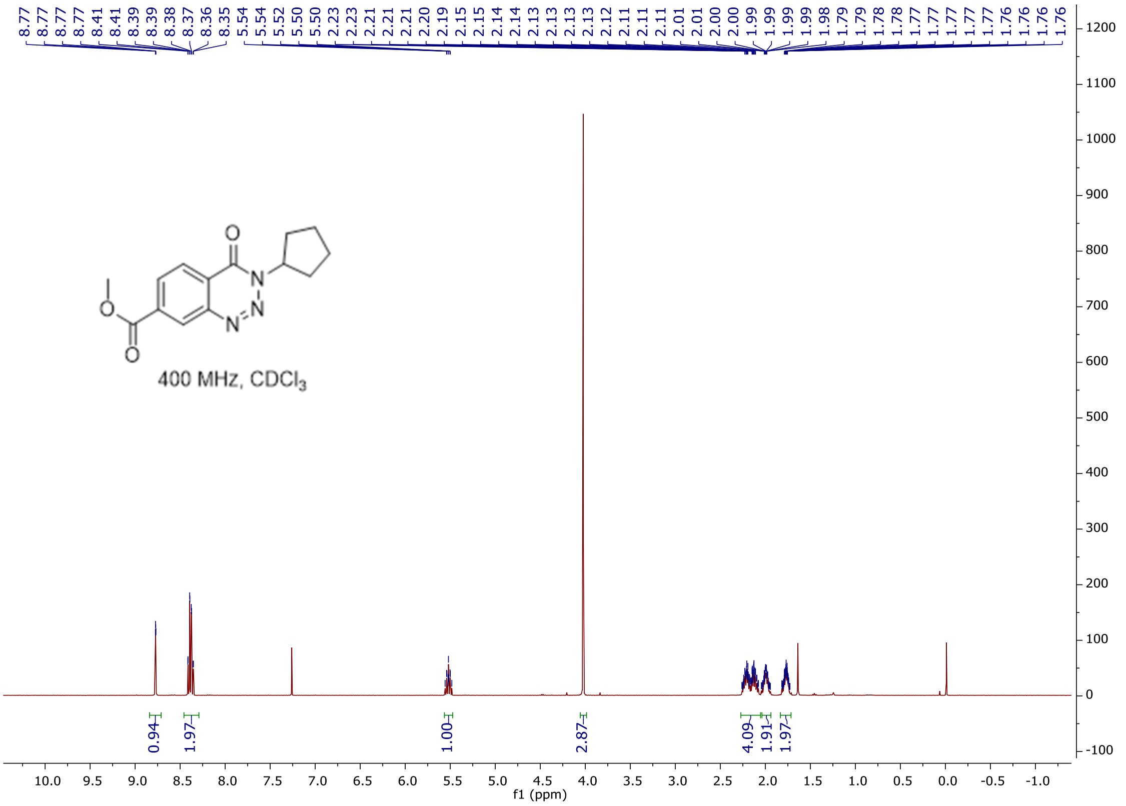

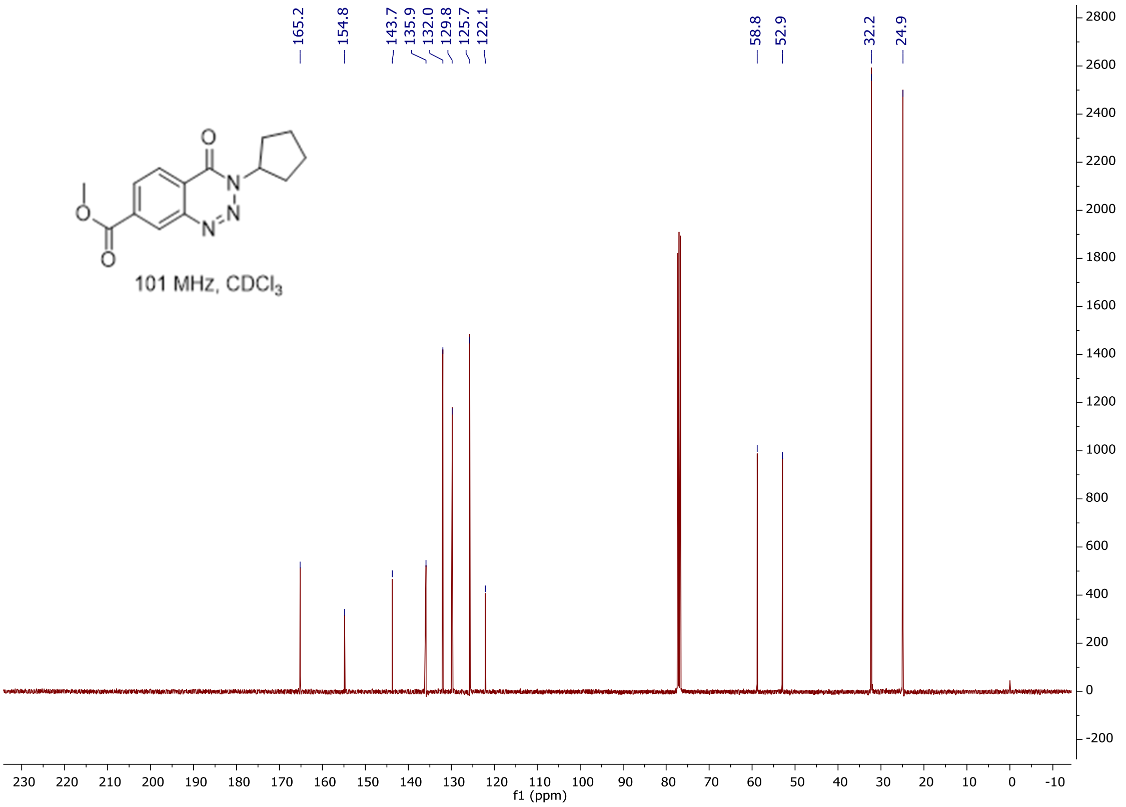

2m: 3-isobutyl-[1,3]dioxolo[4',5':4,5]benzo[1,2-*d*][1,2,3]triazin-4(3*H*)-one

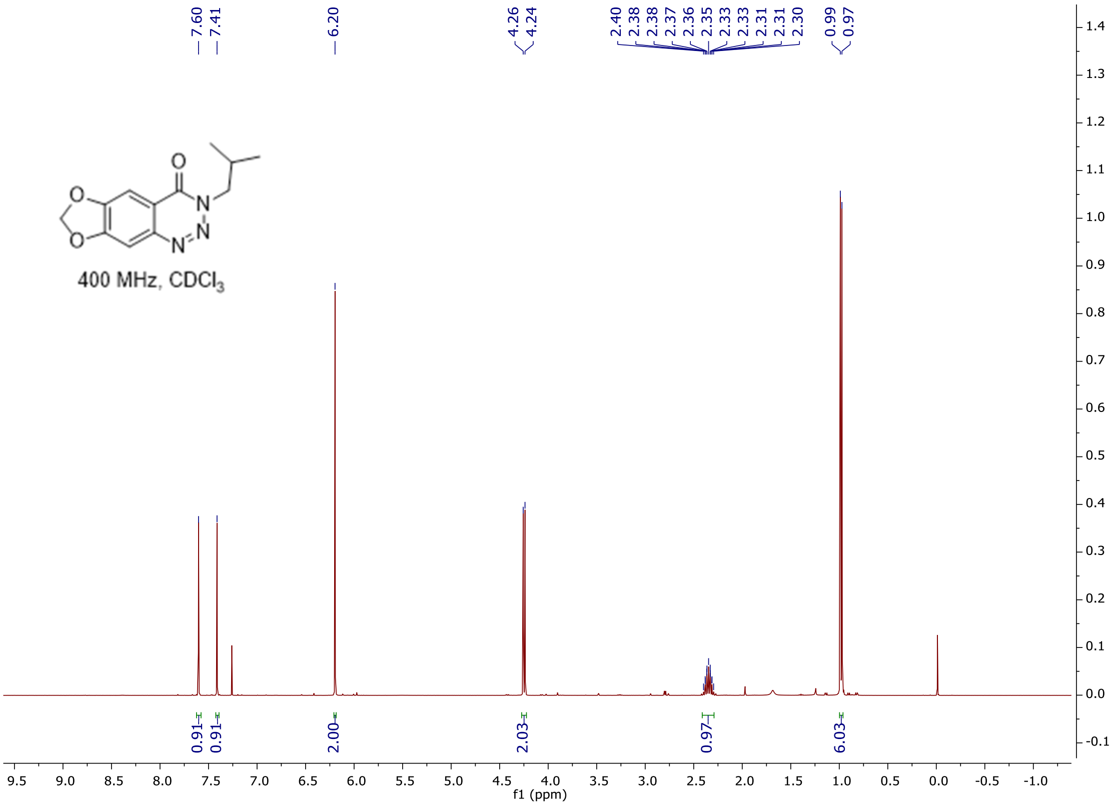

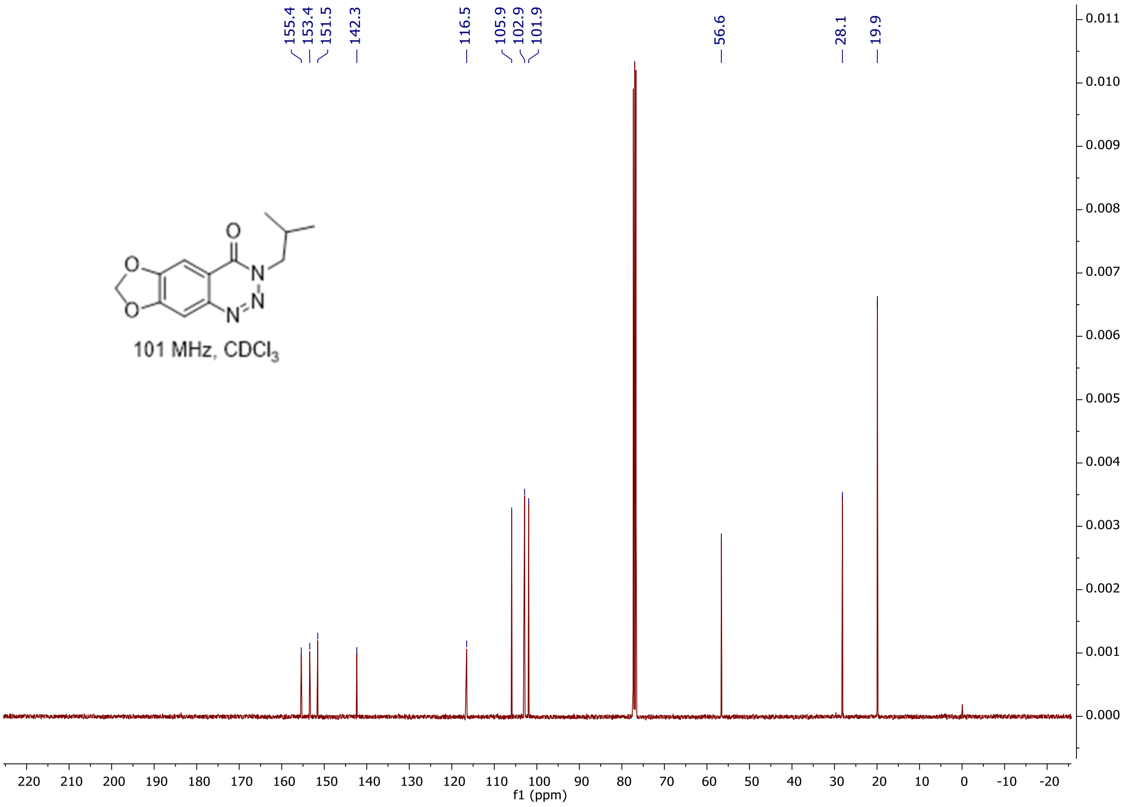

2n: 3-benzyl-6-fluorobenzo[*d*][1,2,3]triazin-4(3*H*)-one

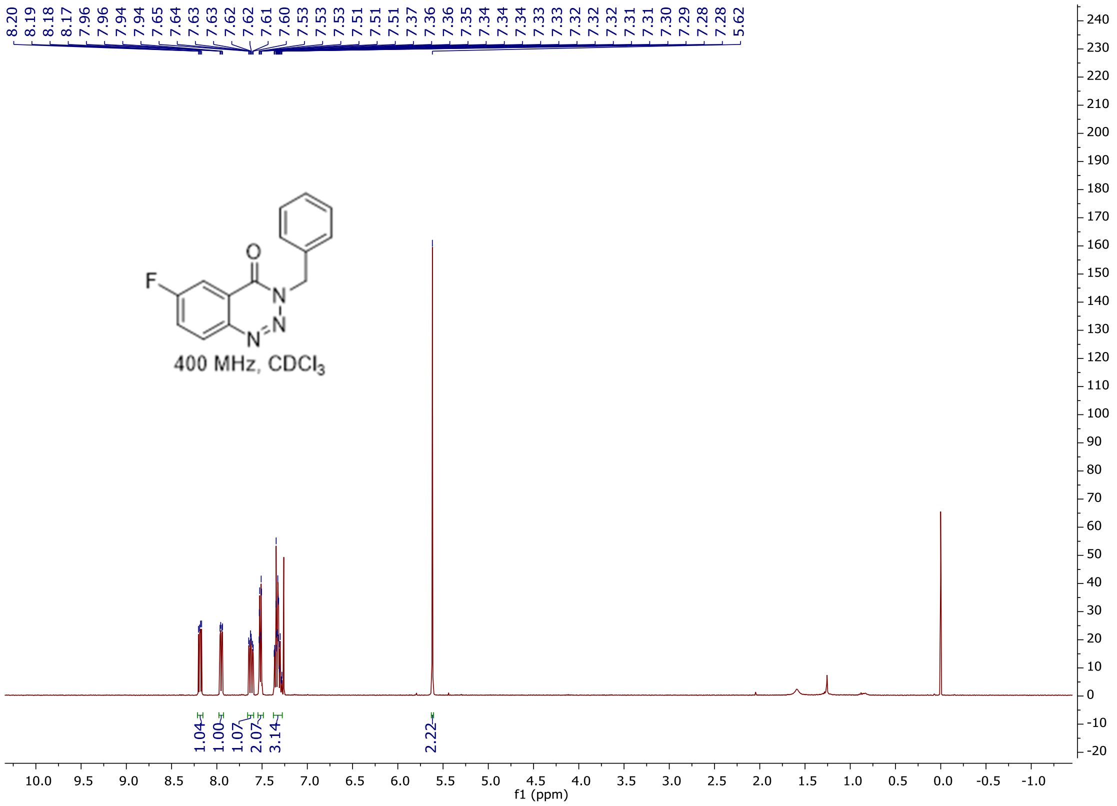

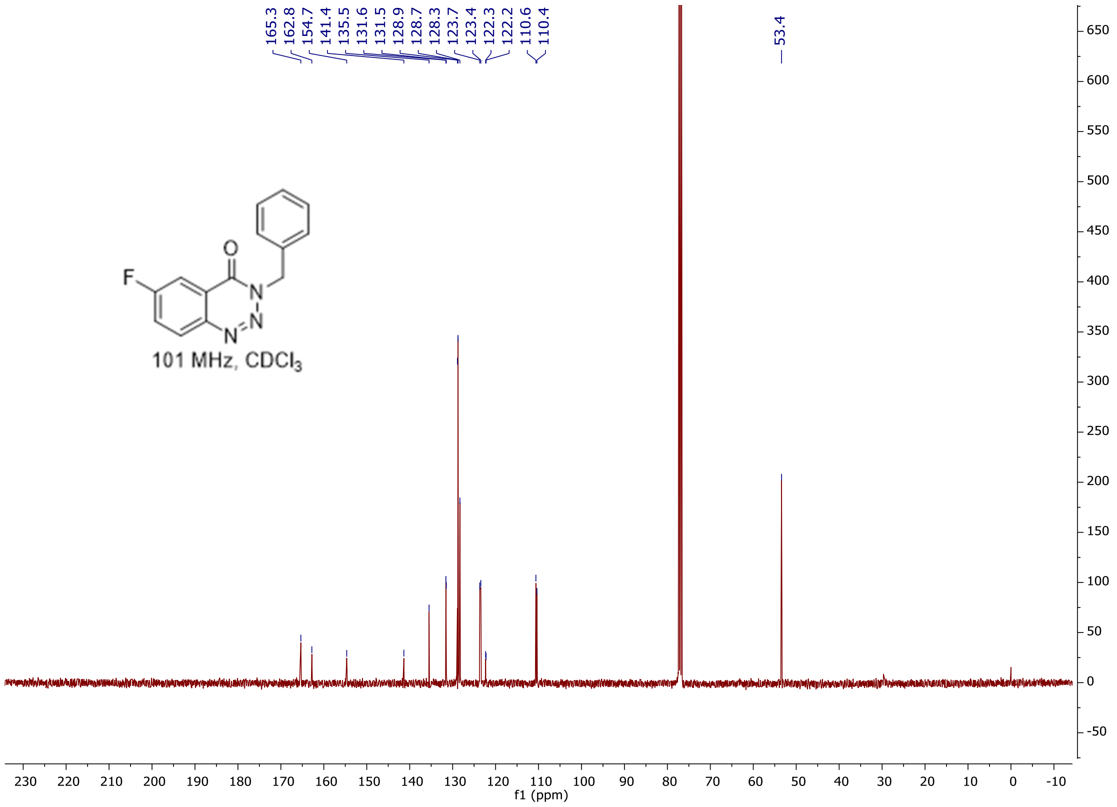

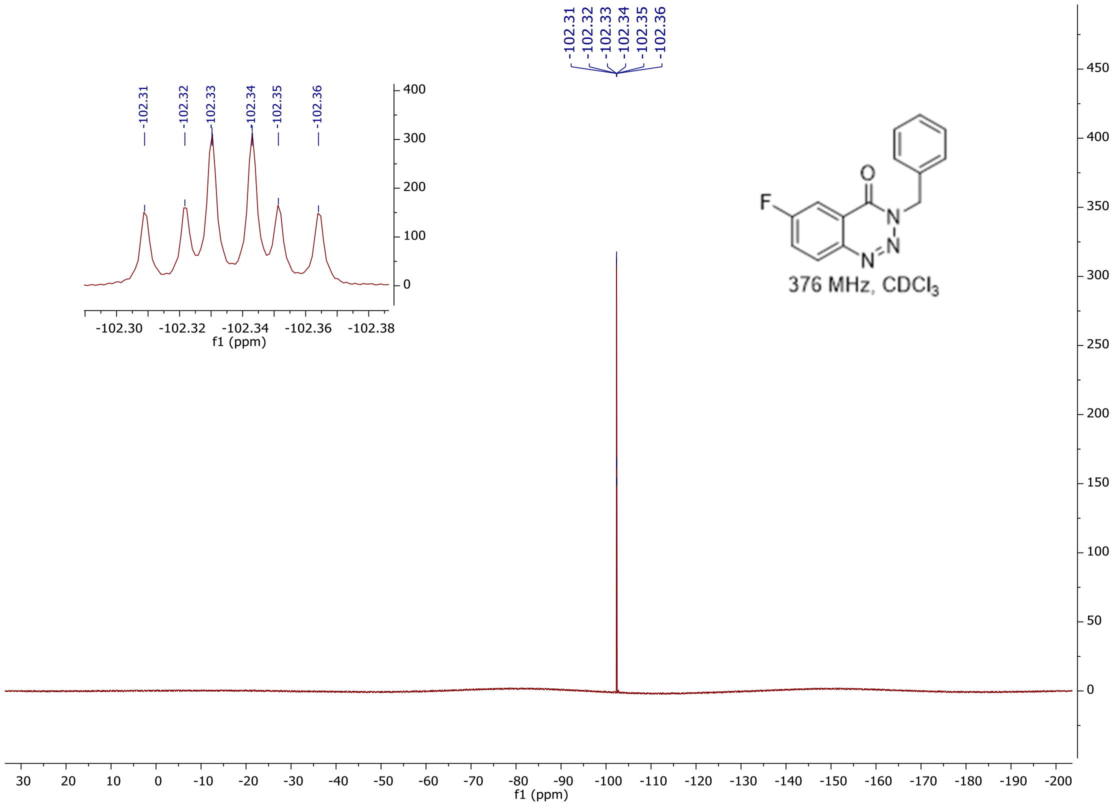

-102.31
-102.32
-102.33
-102.34
-102.35
-102.36
376 MHz, CDCl3
f1 (ppm)

2o: methyl 3-(4-isopropylphenyl)-4-oxo-3,4-dihydrobenzo[*d*][1,2,3]triazine-7-carboxylate

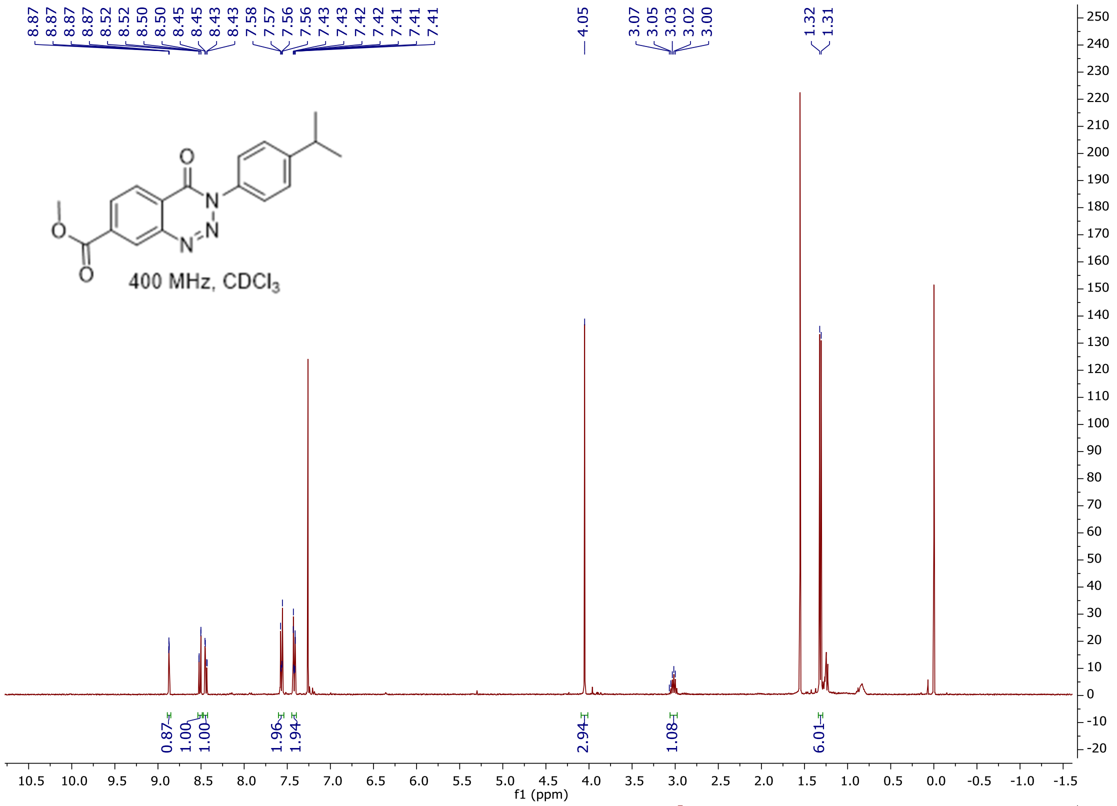

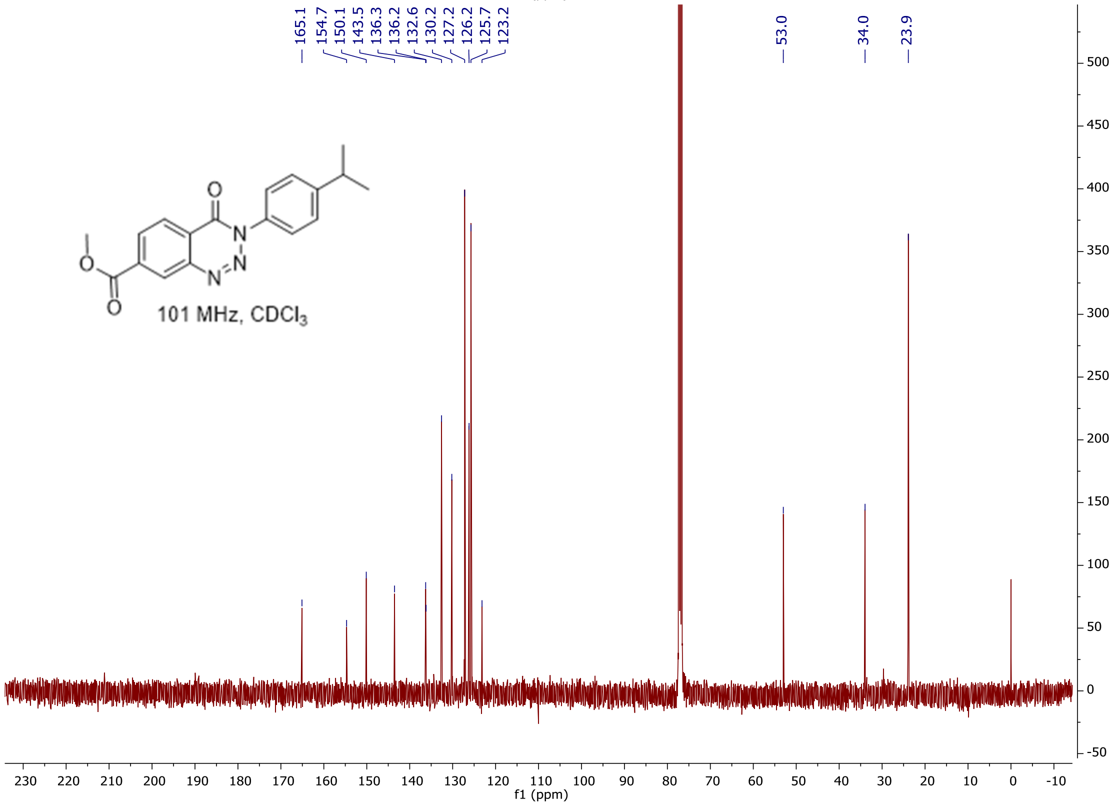

2p: 3-(4-fluorophenyl)-[1,3]dioxolo[4',5':4,5]benzo[1,2-*d*][1,2,3]triazin-4(3*H*)-one

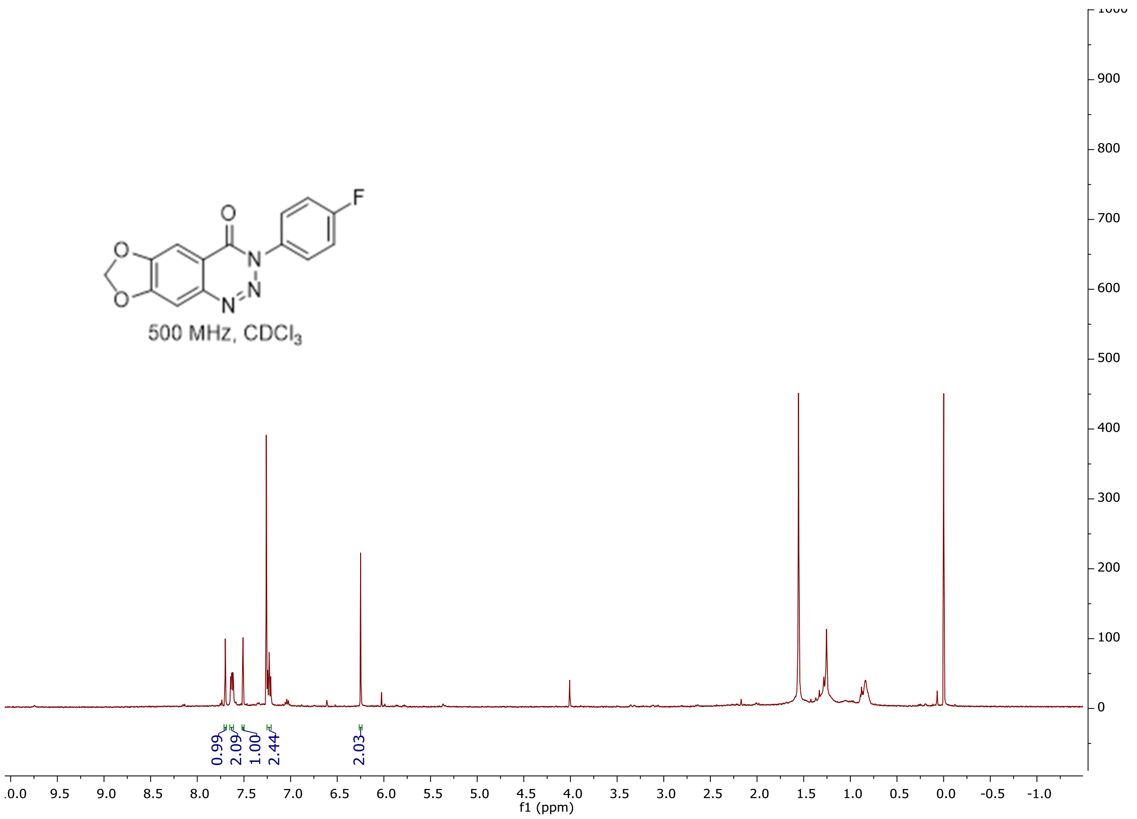

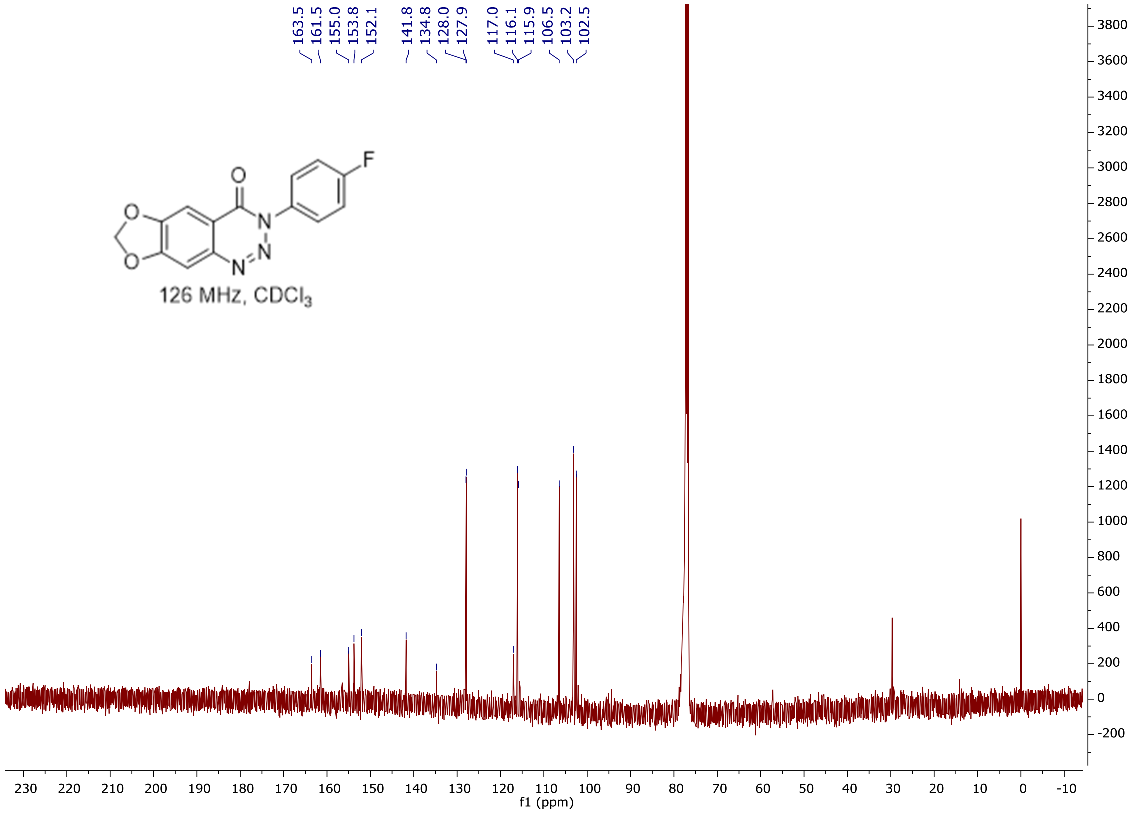

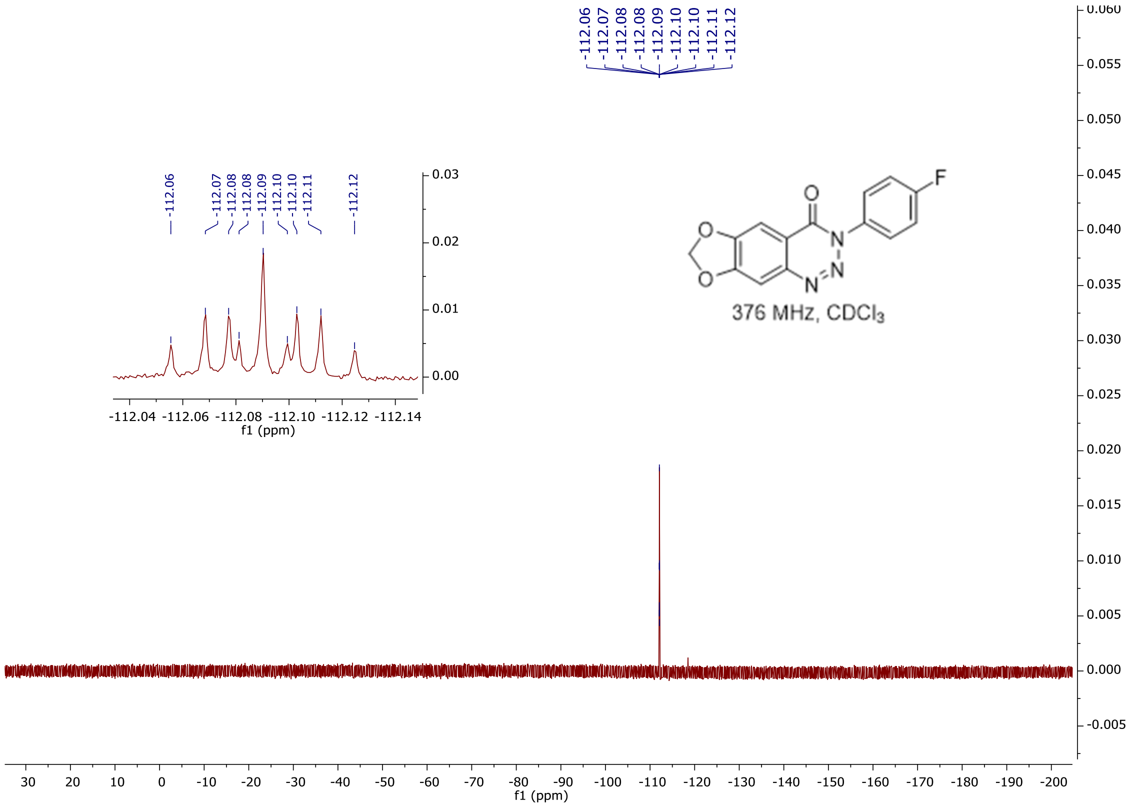
-112.06
-112.07
-112.08
-112.08
-112.09
-112.10
-112.10
-112.11
-112.12
376 MHz, CDCl3
f1 (ppm)
f1 (ppm)

NMR copies of other compounds (3-4)

3: *N*-ethylbenzamide

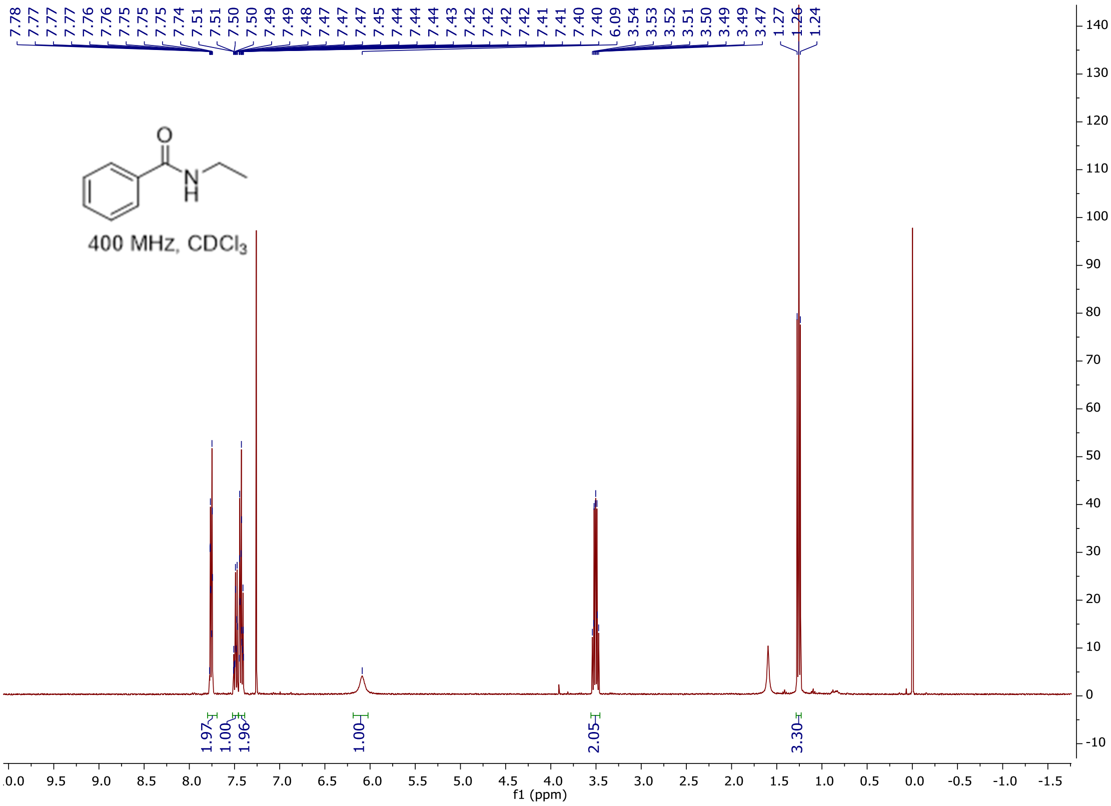

4: benzo[*c*]cinnoline

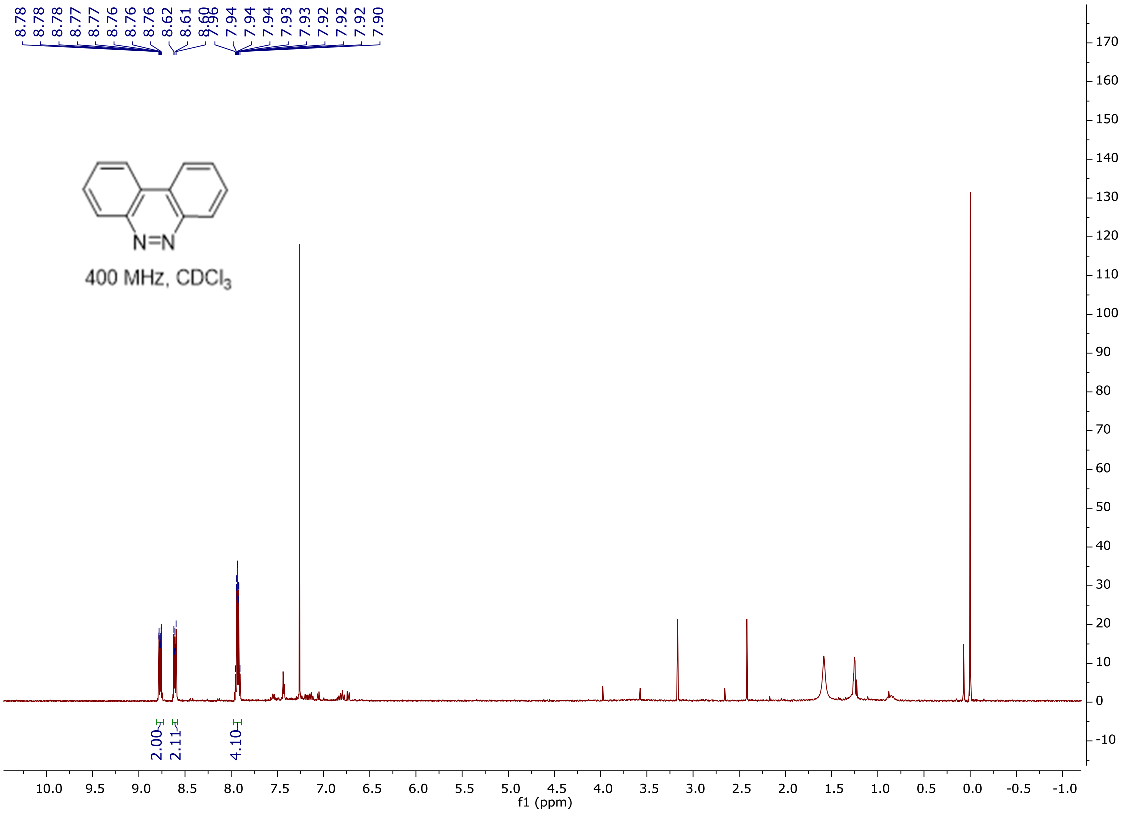

Supplement: Supplementary file 1 — ol4c00248_si_001.pdf [file ol4c00248_si_001.pdf]
